# Erythropoietin alleviate obstructive renal fibrosis by regulating immunity and inflammation through miR-21-5p/SPRY1/ERK1/2/NF-κB pathway inhibition

**DOI:** 10.3389/fmolb.2026.1795772

**Published:** 2026-03-02

**Authors:** Erpeng Liu, Xiao Sun, Qilong Liu, Dongyi Jin, Guihong Li, Huayan Zhao, Hao Sun, Yuming Du

**Affiliations:** 1 Intensive Care Unit, First Affiliated Hospital of Zhengzhou University, Zhengzhou, Henan, China; 2 Henan Academy of Innovations in Medical Science, Zhengzhou, Henan, China; 3 Neurosurgery, First Affiliated Hospital of Zhengzhou University, Zhengzhou, Henan, China; 4 Anyang Center for Disease Control and Prevention, Anyang, China

**Keywords:** erythropoietin (EPO), immunity, inflammation, MiR-21-5p, renal fibrosis, Spry1/ERK/NF-κB, urinary tract obstruction

## Abstract

The important role of erythropoietin (EPO) in the treatment of renal fibrosis induced by urinary tract obstruction has been documented in numerous studies; however, its underlying molecular mechanisms are not yet fully understood, particularly its role in regulating immunity and inflammation. Previous studies have revealed that miR-21 can influence the progression of organ fibrosis by regulating inflammation via activation of the SPRY1/ERK/NF-κB pathway. Additionally, several studies have shown that EPO can exert therapeutic effect by regulating microRNA expression. However, the effect of EPO on miR-21, the NF-κB system (which is associated with innate immunity and inflammation), and specific signaling pathways in the context of obstructive renal fibrosis has rarely been reported. In the present study, we employed a mouse model of unilateral ureteral obstruction (UUO) in which the left ureters were ligated and treated the mice with low-dose rhEPO (100 U/kg) for 7 days, and validated the possible signaling pathway through vitro experiments using HK-2 cells. We found that low-dose rhEPO treatment alleviated the fibrosis and inflammation of obstructive kidneys in mice and the upregulation of miR-21-5p and activation of SPRY1/ERK/NF-κB pathway could be reversed by rhEPO treatment *in vivo* and vitro studies. To the best of our knowledge, this is the first demonstration that EPO exerts anti-fibrotic effect in obstructive renal fibrosis by regulating immunity and inflammation through miR-21-5p/SPRY1/ERK/NF-kB axis.

## 1 Introduction

Renal fibrosis represents the common final outcome of various chronic kidney diseases (CKD) (Liu, 2011). As a chronic inflammatory disorder, CKD is not only associated with diabetes (Peng et al., 2021)^,^ hypertension (Li et al., 2020), and urinary tract obstruction (Gasparitsch et al., 2013), but also particularly closely linked to immunity (Braga et al., 2012) and inflammation (Hassan et al., 2021)-two key factors that strongly drive the migration and activation of leukocytes, as well as the accumulation of excess interstitial fluid (W et al., 2020). These pathological changes ultimately lead to extracellular matrix (ECM) deposition, which is the hallmark pathological feature of renal fibrosis. In urology, urinary tract obstruction is the most frequent reason of renal fibrosis, which damages the normal construction of renal tubules and glomeruli, and ultimately impairs renal function. In clinical practice, the preferred treatment for urinary tract obstruction is to release the obstruction via surgery as soon as possible to protect renal function, especially for acute obstruction. However, this approach is ineffective for chronic urinary obstruction with severe renal fibrosis, and for patients with bilateral lesions, they have to undergo hemodialysis or kidney transplantation to maintain their lives, which imposes a heavy financial burden on the family. Therefore, continuous efforts and time investment are still needed to find breakthrough treatments for obstructive renal fibrosis.

MicroRNAs participate in diverse biological processes by binding to specific sites on target genes to regulate mRNA degradation and protein synthesis, and have long been a research focus in organ fibrosis. miR-21 regulates multiple immunological and developmental processes (Kumarswamy et al., 2011), and is one of the most extensively studied molecules in renal fibrosis research. Studies have demonstrated that inhibition of miR-21 can attenuate renal fibrosis (Zhong et al., 2011; Za et al., 2011), which constitutes the actual working mechanism of certain therapeutic strategies (Wang et al., 2020a; Liu et al., 2020). SPRY1 is a direct target of miR-21 that inhibits ERK activation, and NF-κB is deeply involved in innate immune responses and the regulation of adaptive immunity. The ERK/NF-κB signaling pathway participates in tissue damage and repair processes by regulating the expression of inflammation-related and immune-related genes (Song et al., 2025; Du et al., 2021; Zheng et al., 2021; Choi et al., 2013) and plays a central role in the pathogenesis of various inflammatory diseases. Thus, miR-21 can promote immune responses and inflammation by activating the ERK/NF-κB signaling pathway through targeting SPRY1, thereby exacerbating tissue fibrosis. This mechanism has been validated in pulmonary fibrosis (Sun et al., 2017) and hepatic fibrosis (Ning et al., 2017), suggesting that this pathway may serve as a promising therapeutic target. Therefore, exploring the role of the miR-21/SPRY1/ERK/NF-κB axis, immunity and inflammation in obstructive renal fibrosis is of great significance.

EPO is a glycoprotein hormone primarily produced by renal peritubular cells. Notably, renal fibrosis is associated with impaired renal capacity to generate EPO (Han et al., 2023), though the specific mechanism remains elusive. Studies have demonstrated that EPO can attenuate carbon tetrachloride-induced hepatic fibrosis in mice (Park et al., 2012), diabetic cardiac fibrosis in rats (Lu et al., 2012) and renal fibrosis induced by unilateral ureteral obstruction (UUO) in rats (Lee et al., 2020). Clinical studies have also reported that early EPO administration for anemia in patients with renal failure slows the decline in renal function (Gouva et al., 2004). Therefore, EPO may serve as an effective anti-fibrotic agent and a promising therapeutic strategy for renal fibrosis, but its therapeutic mechanism requires further investigation. Anti-inflammation (Zhang et al., 2020) and immunomodulation (Sarrabayrouse et al., 2024; Chiu et al., 2025) are crucial non-hematopoietic effects of EPO. Published studies have confirmed that EPO inhibits the development of gut microbiota dysbiosis (Sarrabayrouse et al., 2024), autoimmune kidney disease (Donadei et al., 2019) and the tumor microenvironment (Chiu et al., 2025) by regulating immune response. Previous study (Yuen et al., 2017) confirmed that EPO-cyclosporine combination therapy can reduce cerebral infarct size in rats with acute ischemic stroke by suppressing the expression of microRNAs, mitogen-activated protein kinase (MAPK) family signaling, and inflammatory responses. EPO inhibits inflammation and collagen synthesis through the NADPH/ERK1/2/NF-κB signaling pathway, thereby delaying the progression of myocardial fibrosis (Han et al., 2023; Jun et al., 2014; Wang et al., 2016). Another study revealed that EPO exerts a therapeutic effect in glioblastoma by inhibiting microRNA-451 expression (Alural et al., 2017). However, the effect of EPO on the miR-21 expression, inflammation, immunity and its downstream pathways in obstructive renal fibrosis is rarely reported.

In the present study, we performed the UUO models in mice and treated the mice with low-dose rhEPO (100 U/kg) for 7 days. This treatment regimen was based on the experience reported in previous studies (Moriyama et al., 2010; Olgun et al., 2013; Park et al., 2007)and was also intended to minimize the side effects of chronic EPO therapy. Furthermore, we validated the possible signaling pathway through vitro experiments using HK-2 cells. We revealed that low-dose rhEPO treatment alleviated the fibrosis and inflammation of obstructive kidneys in mice by inhibiting the expression of miR-21-5p and the activation of SPRY1/ERK/NF-κB pathway. These findings might help to further understand of the role of EPO in obstructive renal fibrosis.

## 2 Materials and methods

### 2.1 Reagents and antibodies

Recombinant Human Erythropoietin for Injection (rhEPO, S19991025, CHO Cell) was purchased from Shanghai Chemo Wanbang Biophama Co., Ltd. Recombinant Human Transforming Growth Factor β1 (rhTGF-β1, HY-P7118, CHO Cell) was purchased from MedChemExpress. Primary antibodies against α-SMA, collagen I, myeloperoxidase (MPO) and β-actin were purchased from Abcam (Cambridge, United States). Primary antibodies against SPRY1, ERK1/2, p-ERK1/2, NF-κB and p-NF-κB were purchased from Cell Signaling Technology (Massachusetts, United States). Primary antibodies against IL-6 and TNF-α were purchased from Proteintech (Wuhan, China). Horseradish peroxidase (HRP)-labeled goat anti-rabbit secondary antibody was purchased from Sangon Biotech (Shanghai, China). Other reagents are described below.

### 2.2 Animals

We purchased 24 8-week-old male C57BL/6 mice from the experimental animal center of the Medical College of Zhengzhou University, and they were housed at a 22 °C constant room temperature and 47% humidity with a 12-h light-dark cycle and free access to standard laboratory chow and tap water. All experimental procedures on mice were performed in accordance with the National Institutes of Health guidelines and were approved by the Ethical Committee, Animal Care and Use Committee of the First Affiliated Hospital of Zhengzhou University (2020-KY-273).

### 2.3 Mouse model of UUO and experimental groups

We used a mouse UUO model with 7-day ureteral obstruction. All mice used in the present study were randomly divided into four groups: the sham group (n = 6), the UUO group (n = 6), the UUO + EPO group (n = 6) and the UUO + saline group (n = 6). We established UUO model according to the accepted procedures depicted in previous studies. Briefly, the mouse was anesthetized with inhaled isoflurane, and the left proximal ureter was exposed. Then ureter was ligated with 6-0 silk thread and severed. Mice in the sham group received the same surgery as mice in the UUO group, except that the ureters were not ligated and severed. For the mice in the UUO + EPO group, 100 U/kg body wt rhEPO was administered intraperitoneally every day after operation, which lasted 7 days. For the mice in the UUO + saline group, saline was administered intraperitoneally in a manner identical to the rhEPO treatment. All mice in the four groups were sacrificed at the 7th day after operation. The left kidney specimens were collected, and inferior vena cava blood samples were collected for renal function evaluation including blood urea nitrogen (BUN) and serum creatinine (SCr). Part of the kidney tissue was frozen in liquid nitrogen for total RNA and protein extraction, and the remainder was fixed with 4% paraformaldehyde.

### 2.4 Histopathological evaluation

The kidney specimens were fixed with 4% paraformaldehyde and embedded in paraffin. The rotary microtome (Leica, Heidelberg, Germany) was used to cut the specimens into 4-μm sections and subjected to hematoxylin and eosin (HE) staining and Masson’s trichrome. Then the sections were examined and taken pictures by Leica DM4B microscope equipped with Leica X software. At high magnification (×200), we selected six non-overlapping regions of the renal cortex in each section for scoring or image analysis. According to the scoring criteria reported in previous study (Chen and Li, 2018), the tubulointerstitial impairment was assessed, which included tubular atrophy, tubular necrosis, lymphocyte infiltration, and interstitial fibrosis. The scores for each criterion were as follows: 0 = none; 1 = mild or <25%, 2 = moderate or 25%–50%, and 3 = severe or >50%. The blue stained area stained by Masson was considered to be collagen deposition and was analyzed using Image-Pro plus 6.0 software and the results were expressed as the means ± SD.

### 2.5 Immunohistochemistry (IHC)

Using a rotary microtome, the paraffin-embedded kidney specimens were cut into 4-μm sections, then deparaffinized with xylene, and rehydrated with graded ethanol (100%, 95%, 85%, and 75%) and distilled water. The sections were incubated with 3% hydrogen peroxide for 10 min to block the activity of endogenous peroxidase, and then microwaved in 0.01 mol/L citrate buffer (pH 6.0) for 25 min for antigen retrieval. We washed Sections 5 min using phosphate buffered saline (PBS), 3 times in total, and then incubated the sections with the primary antibodies, including anti-α-SMA (1:500), anti-collagen I (1:500), anti-TNF-α (1:300), anti-IL-6 (1:200), and anti-MPO (1:1000), overnight at 4 °C. Next day, the sections were washed with PBS, and then incubated with HRP-labeled goat anti-rabbit secondary antibody at room temperature for 1 h. Finally, dehydration, clearing, 3,3′-diaminobenzidine (DAB) staining and neutral resin sealing were performed in sequence. Then the sections were examined and taken pictures by Leica DM4B microscope equipped with Leica X software. At high magnification (×200), we selected six non-overlapping regions of the renal cortex in each section for image analysis. Image-Pro plus 6.0 software was used to evaluate the integral optical density (IOD) of the positive area, and the results were expressed as the means ± SD.

### 2.6 Cell culture

We purchased the human renal proximal tubular epithelial cell line HK-2 from Beina Chuanglian Biotechnology Institute (Beijing, China), and cultured the cells in a humidified atmosphere of 5% CO2 at 37 °C using Dulbecco’s modified Eagle medium/nutrient mixture F-12 (DMEM/F12), which was supplemented with 10% (v/v) fetal bovine serum (FBS), 100 IU/mL penicillin, and 10 mg/mL streptomycin. Cells were seeded in 6-well plate at a density of 1 × 10^5^ cells per well and cultured in 10% FBS complete medium for 24 h. The cells were then cultured in serum-free medium for 24 h, and then treated with rhTGF-β1 at 10 ng/mL in 1% FBS medium for 24 h to induce fibrosis. The concentration and time of rhEPO treatment for HK-2 cells were determined through establishing gradient, the fibrotic HK-2 cells induced by rhTGF-β1 were treated with rhEPO at concentration of 10 U/mL for 0 h, 12 h, 24 h, 48 h respectively, or treated with rhEPO for 24 h at concentration of 0 U/mL, 5 U/mL, 10 U/mL, 20 U/mL respectively.

### 2.7 Quantitative real-time polymerase chain reaction (qRT-PCR)

Trizol (Sangon Biotech, B511311, Shanghai, China) was used to extract total RNA, which was stored at −80 °C and checked the concentration and quality via NanoDrop 2000 UV-Vis spectrophotometer (Thermo Scientific, United States). Then reverse transcription of total RNA was conducted using M-MuLV First-Strand cDNA Synthesis kit (Sangon Biotech, B532435, Shanghai, China). The 2×SG Fast qPCR Master Mix (Low Rox) kit (Sangon Biotech, B639272, Shanghai, China) was used to determine the levels of mRNA through the Applied Biosystems 7,500 Sequence Detection System. Each sample was analyzed in triplicate. The β-actin gene served as a control, and the data were analyzed through the 2^−(ΔΔCt)^ method. To detect the expression level of miR-21-5p, the microRNA First-Strand cDNA Synthesis kit (Sangon Biotech, B532453, Shanghai, China) was used to synthesize cDNA following the manufacturer’s protocol. A MicroRNAs Quantitation PCR Kit (Sangon Biotech, B532461, Shanghai, China) was used to conduct qRT-PCR, and an Applied Biosystems 7,500 Sequence Detection System was applied to detect the levels of miR-21-5p. Each sample was analyzed in triplicate. U6 small nuclear RNA served as a control, and the data were analyzed through the 2^−(ΔΔCt)^ method. The sequences of all primers are listed in Table 1.

**TABLE 1:** Primers used for reverse transcription and real-time PCR.

Primer Name		Sequence
mmu-α-SMA	Sense Anti-sense	CTGTTATAGGTGGTTTCGTGGA GAGCTACGAACTGCCTGAC
mmu-Collagen I	Sense Anti-sense	CTTCACCTACAGCACCCTTGTG GATGACTGTCTTGCCCCAAGTT
mmu-U6	Stem-loop Sense Anti-sense	GTCGTATCCAGTGCAGGGTCCGAGGTATTCGCACTGGATACGACAAAATATG CTCGCTTCGGCAGCACA AACGCTTCACGAATTTGCGT
mmu-miR-21-5p	Stem-loop Sense Anti-sense	GTCGTATCCAGTGCAGGGTCCGAGGTATTCGCACTGGATACGACTCAACA ACACTCCAGCTGGGTAGCTTATCAGACTGA TGGTGTCGTGGAGTCG
hsa-U6	Stem-loop Sense Anti-sense	CGAGCACAGAATCGCTTCACGAATTTGCGTGTCAT CGAGCACAGAATCGCTTCA CTCGCTTCGGCAGCACATAT
hsa-miR-21-5p	Stem-loop Sense Anti-sense	GTCGTATCCAGTGCAGGGTCCGAGGTATTCGCACTGGATACGACACAGCCG CGGCGCAACACCAGTCGATG AGTGCAGGGTCCGAGGTATT
Mmu-β-actin	Sense Anti-sense	AGAGGGAAATCGTGCGTGAC CAATAGTGATGACCTGGCCGT

### 2.8 Western blotting (WB) assay

According to the manufacturer’s instructions, the total protein was isolated from kidney tissues and HK-2 cells using efficient radioimmunoprecipitation assay (RIPA) tissue/cell lysis buffer (Solarbio, R0010, Beijing, China). Enhanced BCA Protein Assay Kit (Beyotime, P0009, Shanghai, China) was used to detect the concentration of total protein. The protein sample was separated by sodium dodecyl sulfate-polyacrylamide gel electrophoresis, loading amount was 30 µg per well. In transfer solution, the protein was transferred from the resolving gel to the PVDF membrane by electrophoresis. The membrane was blocked with 5% nonfat milk on a shaker for 2 h and then incubated with primary antibodies overnight at 4 °C. Primary antibodies included anti-collagen I (1:1000), anti-α-SMA (1:1000), anti-Spry1 (1:500), anti-ERK1/2 (1:1000), anti-p-ERK1/2 (1:1000), anti-NF-κB (1:500), anti-p-NF-κB (1:500), anti-IL-6 (1:500), anti-TNF-α (1:500), and anti-β-actin (1:2000). TBST solution was used to wash the membrane three times in 30 min. Then, at room temperature, the membrane was incubated with HRP-labeled goat anti-rabbit secondary antibody on a shaker for 1 h. After washing the membrane three times again in TBST solution, the Omni-ECL™ Femto Light Chemiluminescence kit (EpiZyme, SQ201, Shanghai, China) was used to visualize the bands in the membrane through the Bio-Rad ChemiDoc™ MP Imaging System. ImageJ software was used to quantify the densities of the bands. The levels of β-actin served as controls, and the results were expressed as the means ± SD.

### 2.9 Cell transfection

The pGV514-miR-21-5p-mimics plasmid for overexpressing miR-21-5p and the corresponding control plasmid were constructed and synthesized by GeneChem (Shanghai, China). The transfection was conducted using lipofectamine 3,000 reagent (Invitrogen) according to the manufacturer’s instructions. The cells were seeded in 6-well plate at a density of 1 × 10^5^ cells per well and transfected at 60%-70% confluence, and then harvested after 48 h of transfection for subsequent experiments.

### 2.10 Immunofluorescence

Glass coverslips were placed in wells of 6-well plate in advance and HK-2 cells were seeded at 1 × 10^5^ per well. Then the cells adhered to the coverslips, which were taken out and washed with PBS. 4% formaldehyde solution was used to fix the cells for 30 min, and then the cells were permeabilized with 0.2% Triton X-100/PBS for 15 min. Thereafter, we blocked the cells with 2% bovine serum albumin for 30 min and then incubated the cells in primary antibodies overnight at 4 °C. Next day, the cells were washed with PBS, followed by incubation with FITC-/TRITC-conjugated secondary antibodies for 1 h at room temperature, and then DAPI staining was performed after washing with PBS. At last, the coverslips were checked and taken pictures under fluorescence microscope.

### 2.11 Statistical analysis

GraphPad Prism seven was used to determine statistically significant differences. All data are presented as the means ± SD and were analyzed using one-way analysis of variance with Tukey’s test. *P* < 0.05 was considered statistically significant.

## 3 Results

### 3.1 The rhEPO treatment could significantly alleviate the renal cortex collagen deposition and improve renal function in UUO model of mice

**FIGURE 1:**
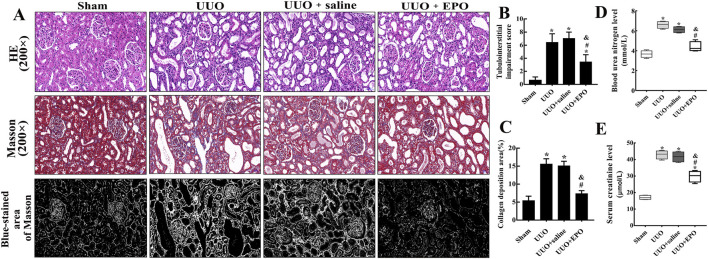
The histopathological change of kidneys and renal function were improved by low dose rhEPO treatment in UUO model of mice. **(A)** Representative images of renal cortex in different groups of mice after HE staining (×200) and Masson’s trichrome staining (×200), scale bars represent 50 μm. The images of Blue-stained area of Masson were transformed from representative images of Masson’s trichrome staining using Image-Pro plus 6.0 software, the white area refers to the collagen deposition area. **(B,C)** Statistical analyses of tubulointerstitial damage scores and the collagen deposition areas in different groups of mice (mean ± SD, n = 6). **(D,E)** Statistical analyses of BUN and SCr levels in different groups of mice (mean ± SD, n = 6). ^*^ *P* < 0.05, compared to the sham group. ^#^ *P* < 0.05, compared to the UUO group. ^&^ *P* < 0.05, compared to the UUO + saline group.

HE staining showed that 7 days after the left ureters of mice were ligated, the proximal renal tubules in the cortex of UUO group were significantly dilated and the tubulointerstitial impairment score was increased obviously according to the score criterions (Figures 1A,B). Due to compensation by the right kidneys, the BUN and SCr levels of the UUO group and the UUO + saline group were roughly within the normal range (Table 2), but compared with Sham group, the upward trend within the normal range was still obvious (Figures 1D,E). The blue-stained area of Masson’s trichrome staining expanded significantly in UUO group (Figure 1C). However, comparing UUO + EPO group with UUO group or UUO + saline group showed that the tubulointerstitial impairment score, the renal function and the blue-stained area of Masson’s trichrome staining were decreased significantly.

**TABLE 2:** BUN and SCr levels in the four groups of mice (mean ± SD, n = 6).

	Sham	UUO	UUO + saline	UUO + EPO
BUN (mmol/L)	3.663 ± 0.3572	6.608 ± 0.3517*	6.128 ± 0.3134*	4.390 ± 0.4956^#&^
SCr (μmol/L)	17.00 ± 1.287	42.91 ± 2.558*	41.61 ± 3.126*	29.81 ± 3.339*^#&^

The normal BUN, level in mice is 3.86–12.41 mmol/L. The normal SCr, level in mice is 10.91–85.09 μmol/L ^*^ *P* < 0.05, compared to the sham group. ^#^ *P* < 0.05, compared to the UUO, group. ^&^ *P* < 0.05, compared to the UUO + saline group.

### 3.2 The upregulation of fibrosis indicators could be reversed by the rhEPO treatment in kidneys of UUO model in mice

**FIGURE 2:**
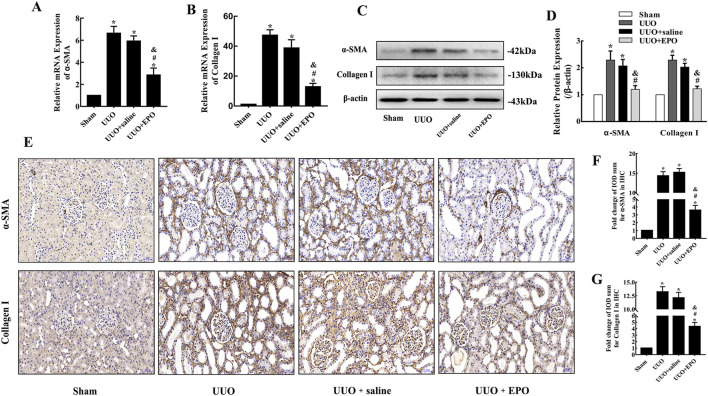
The fibrosis indicators were increased in kidneys of UUO model in mice, and the upregulation could be reversed by rhEPO treatment. **(A,B)** Relative α-SMA and collagen I mRNA expression in kidneys of different groups in mice determined by qRT-PCR (mean ± SD, n = 3). **(C,D)** Representative bands and fold changes in α-SMA and collagen I protein expression in kidneys of different groups in mice determined by Western blotting (mean ± SD, n = 3). **(E–G)** Representative IHC images and fold changes for α-SMA and collagen I in kidneys of different groups in mice (×200), scale bar represents 50 μm (mean ± SD, n = 6). ^*^ *P* < 0.05, compared to the sham group. ^#^ *P* < 0.05, compared to the UUO group. ^&^ *P* < 0.05, compared to the UUO + saline group.

The results of qRT-PCR showed that the expression levels of α-SMA and Collagen Ι were increased significantly after 7-day obstruction in kidneys of UUO group, but the elevations were inhibited by low dose rhEPO treatment in UUO + EPO group (Figures 2A,B). The similar results were also revealed by Western blotting (Figures 2C,D) and immunohistochemical staining (Figures 2E–G).

### 3.3 The rhEPO treatment could reverse the upregulation of miR-21-5p and the activation of SPRY1/ERK/NF-κB signaling pathway in kidneys of UUO model in mice

**FIGURE 3:**
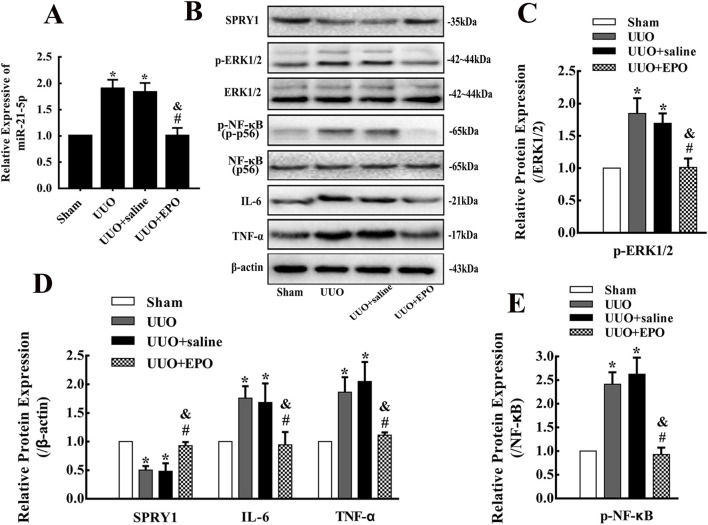
The upregulation of miR-21-5p and the activation of SPRY1/ERK/NF-κB signaling pathway in kidneys of UUO model in mice could be reversed by rhEPO treatment **(A)** miR-21-5p levels in kidneys of different groups in mice determined by qRT-PCR (mean ± SD, n = 3). **(B–E)** Representative bands and fold changes in Spry1, p-ERK1/2, ERK1/2, p-NF-κB, NF-κB, IL-6 and TNF-α protein expression in kidneys of mice in different groups, determined by Western blotting (mean ± SD, n = 3).^*^ *P* < 0.05, compared to the sham group. ^#^ *P* < 0.05, compared to the UUO group. ^&^ *P* < 0.05, compared to the UUO + saline group.

The levels of miR-21-5p were elevated obviously in kidneys of UUO group according to the results of qRT-PCR (Figure 3A). While the protein expression levels of SPRY1 decreased, the protein expression levels of p-ERK1/2, p-NF-κB, IL-6 and TNF-α increased, as shown by Western blotting in UUO group (Figures 3B–E). Impressively, the 7-day low dose rhEPO treatment in UUO + EPO group could reverse the results listed above according to the results of qRT-PCR and Western blotting.

### 3.4 The rhEPO treatment could ameliorate the inflammatory response of the renal cortex in the UUO model of mice

**FIGURE 4:**
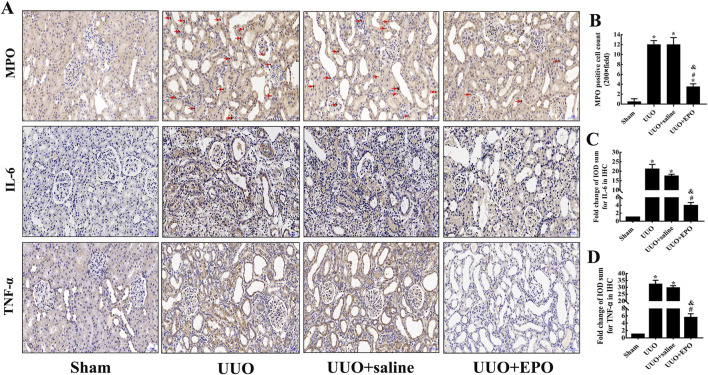
The inflammatory response of the renal cortex in the UUO model of mice was ameliorated by rhEPO treatment. **(A)** Representative IHC images of MPO, IL-6 and TNF-α in kidneys of different groups in mice (×200), scale bar represents 50 μm. **(B)** MPO positive cells count in the renal cortex of different groups in mice (mean ± SD, n = 6). **(C,D)** Fold changes in IL-6 and TNF-α levels in kidneys of different groups in mice (mean ± SD, n = 6). ^*^ *P* < 0.05, compared to the sham group. ^#^ *P* < 0.05, compared to the UUO group. ^&^ *P* < 0.05, compared to the UUO + saline group.

The results of immunohistochemical staining showed that MPO-positive inflammatory cells were increased and the expression of IL-6 and TNF-α was enhanced significantly in the renal cortex after 7-day obstruction in kidneys of UUO group. In the UUO + EPO group, the intervention with 7-day low dose rhEPO treatment in mice could decrease the MPO-positive inflammatory cells and inhibit the expression of IL-6 and TNF-α (Figure 4).

### 3.5 EPO could alleviate the fibrosis of HK-2 cells induced by TGF-β1 through inhibiting the expression of miR-21-5p

**FIGURE 5:**
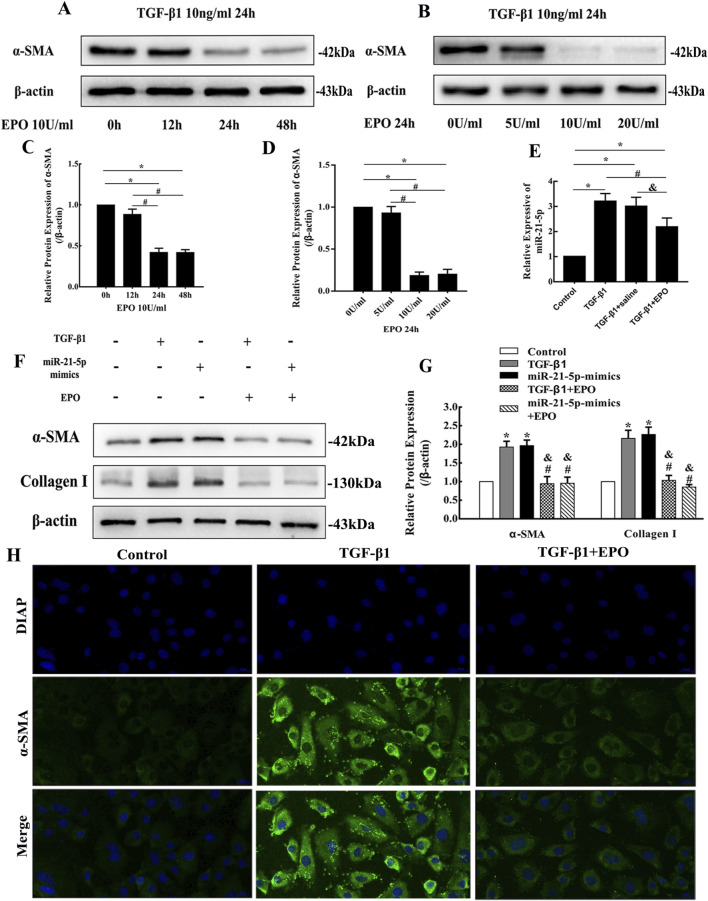
EPO could alleviate the fibrosis of HK-2 cells induced by TGF-β1 through inhibiting the expression of miR-21-5p. **(A–D)** Representative bands and fold changes in α-SMA protein expression in HK-2 cells induced by concentration and time gradient of TGF-β1, determined by Western blotting (mean ± SD, n = 3). ^*^ *P* < 0.05, compared to 0 h or 0 U/mL. ^#^ *P* < 0.05, compared to 12 h or 5 U/mL. **(E)** miR-21-5p levels in HK-2 cells treated with TGF-β1 and rhEPO determined by qRT-PCR (mean ± SD, n = 3). ^*^ *P* < 0.05, compared to the control group. ^#^ *P* < 0.05, compared to the TGF-β1 group. ^&^ *P* < 0.05, compared to the TGF-β1 + saline group. **(F,G)** Representative bands and fold changes in α-SMA and collagen I protein expression in HK-2 cells treated with TGF-β1 or rhEPO or miR-21-5p-mimic, determined by Western blotting (mean ± SD, n = 3). ^*^ *P* < 0.05, compared to the control group. ^#^ *P* < 0.05, compared to the TGF-β1 group. ^&^ *P* < 0.05, compared to the miR-21-5p-mimic group. **(H)** Immunofluorescence staining of α-SMA (×400) in HK-2 cells showing that TGF-β1 increased α-SMA expression, whereas treatment with rhEPO reversed this effect; scale bar represents 20 μm.

**FIGURE 6:**
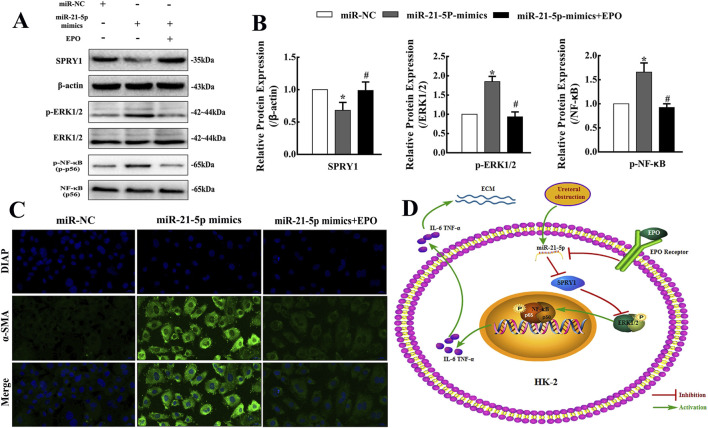
Pro-fibrosis effect of miR-21-5p in HK-2 cells was inhibited by EPO through regulating SPRY1/ERK/NF-kB signaling pathway. **(A,B)** Representative bands and fold changes in Spry1, p-ERK1/2, ERK1/2, p-NF-kB and NF-kB protein expression in HK-2 cells treated with rhEPO or miR-21-5p-mimic, determined by Western blotting (mean ± SD, n = 3). ^*^ *P* < 0.05, compared to the miR-NC group. ^#^ *P* < 0.05, compared to the miR-21-5p-mimic group. **(C)** Immunofluorescence staining of α-SMA (×400) in HK-2 cells showing that miR-21-5p-mimic transfection increased α-SMA expression, whereas treatment with rhEPO reversed this effect; scale bar represents 20 μm. **(D)** Mode pattern of the EPO-miR-21-5p-SPRY1/ERK/NF-kB regulatory network in obstructive renal fibrosis.

The induction of HK-2 cells by rhTGF-β1 at 10 ng/mL for 24 h promoted the expression of α-SMA and Collagen Ι, and resulted in conversion of cell morphology to be spindle-shaped (Figures 5F,H). Results of the time gradient with 10 U/mL rhEPO in treatment of HK-2 cells revealed that the expression of α-SMA in HK-2 cells induced by rhTGF-β1 was decreased significantly after intervention for 24 h and 48 h (Figures 5A,C). And results of the concentration gradient with rhEPO intervention for 24 h showed that the rhEPO concentration at 10 U/mL and 20 U/mL inhibited the expression of α-SMA significantly (Figures 5B,D). So the concentration and time of rhEPO treatment for HK-2 cells were set to 10 U/mL for 24 h in the subsequent experiments. According to the results of Western blotting, the treatment with rhEPO could reverse the upregulation of α-SMA and Collagen Ι causing by rhTGF-β1 induction or transfection with miR-21-5p mimics in HK-2 cells (Figures 5F,G), and immunofluorescence staining showed the consistent results with Western blotting (Figures 5H, 6C). The rhTGF-β1 induction promoted the expression of miR-21-5p, but the upregulation was inhibit after treatment with rhEPO (Figure 5E).

### 3.6 EPO could attenuate pro-fibrosis effect of miR-21-5p via inhibiting SPRY1/ERK/NF-κB signaling pathway in HK-2 cells

The transfection with miR-21-5p mimics in HK-2 cells could increase the expression of α-SMA and Collagen Ι showing by the results of Western blotting and immunofluorescence staining, but the effect could be counteracted by rhEPO (Figures 5F,G, 6C). Moreover, the transfection with miR-21-5p mimics in HK-2 cells increased p-ERK1/2 and p-NF-κB protein levels, but decreased SPRY1 protein levels. However, the treatment with rhEPO reversed the SPRY1/ERK/NF-κB pathway activation, in which the protein level of SPRY1 was increased, but p-ERK1/2 and p-NF-κB were decreased (Figures 6A,B).

Taken together, our results indicate that the low-dose rhEPO treatment has obvious anti-inflammatory and anti-fibrotic effects on obstructive kidneys in mice, which may be achieved by inhibiting miR-21-5p/SPRY1/ERK/NF-κB signaling pathway (Figure 6D).

## 4 Discussion

The anti-fibrotic effect of EPO has been well-documented in numerous other organs, including the liver (Park et al., 2012; Wang et al., 2020b), heart (Lu et al., 2012; Liu et al., 2018), skeletal muscle (Wu et al., 2020). Initially, the rationale for investigating EPO lies in its capacity to exert a prominent protective effect against injuries of various organs and tissues (Park et al., 2012; Lu et al., 2012; Bagnis et al., 2001). In ischemia-reperfusion injury (IRI) of kidney, previous studies have confirmed that EPO can reduce macrophage infiltration, and promote renal tubular repair and regeneration (Han et al., 2023; va et al., 2013; Imamura et al., 2012; Chen et al., 2015), suggesting that its protective effect may be attributed to regulation of immunity. In the present study, 7 days of ureteral obstruction induced left renal fibrosis in mice, low-dose rhEPO treatment significantly reduced the tubulointerstitial injury score and markedly improved renal function, the treatment also attenuated collagen deposition in the renal cortex and suppressed the expression of renal fibrosis markers, which is consistent with the findings of previous studies (Lee et al., 2020; Geng et al., 2015; Acikgoz et al., 2014). Thus, most researchers concur that EPO exerts anti-fibrotic effect and possesses nephroprotective property in renal injury (Lee et al., 2020; Gouva et al., 2004; Zhang et al., 2020; Elbaset et al., 2023).

Inflammation, a specific manifestation of the immune response in local tissues that serves as the body’s defensive mechanism against injury, infection, or other stimuli, persists throughout the development of CKD and plays a promotional role (Morgado-Pascual et al., 2018), tissue damage activates the immune system, followed by the migration of immune or inflammatory cells—a subset of which express MPO, such as neutrophils, monocytes, and macrophages—to the injury site. These cells release inflammatory cytokines, thereby triggering an inflammatory response. These fall within the scope of innate immune responses. In this study, results demonstrated that cytokines including IL-6 and TNF-α were elevated significantly, and the MPO-positive cells infiltrated the renal cortex in mice of UUO group. In contrast, in the UUO + EPO group, 7-day low-dose rhEPO treatment significantly inhibited the expression of IL-6 and TNF-α, and the number of MPO-positive cells was also distinctly reduced. Therefore, EPO can effectively ameliorate the inflammatory and immune response in the renal cortex during obstructive renal fibrosis, which is also consistent with previous reports (Zhang et al., 2020; Elbaset et al., 2023; Oh et al., 2025). However, the underlying mechanisms of its immune regulation remain highly complex and incompletely understood.

The prominent pathogenic role of innate immunity in CKD has been well-established in various experimental models recently (Foresto-Neto et al., 2020; Zambom et al., 2019; Faustino et al., 2018; Albino et al., 2021). In principle, activation of innate immunity induced by membrane debris and biomolecules released following renal IRI or toxic cell damage should be self-limited and thus cease once tubular regeneration is completed. While if innate immunity activation persists, the resulting inflammation could lead to continuing renal damage. Accumulating evidence (Foresto-Neto et al., 2020; Faustino et al., 2018; Albino et al., 2021) has demonstrated that chronic activation of NF-κB system—which regulates a multitude of genes critical to innate immunity and inflammation (Ma et al., 2011)—is the key to sustaining the insidious process of renal inflammation that ultimately progresses to CKD by driving a complex transition to renal fibrosis. Our results revealed that the expression level of p-NF-κB was increased in kidneys of UUO group, indicating that the NF-κB system was activated and that innate immunity is involved in inflammatory process of obstructive renal fibrosis. Likewise the low dose rhEPO treatment in the UUO + EPO group could reduce the p-NF-κB expression, suggesting that EPO may exert anti-inflammatory and anti-fibrotic effect by regulating NF-κB system and innate immunity. However, the specific signaling pathways involved warrant further in-depth investigation. Meanwhile, studies have confirmed that EPO can physiologically regulate the differentiation of IL-17-producing CD4^+^ T cells (Th17 cells), thereby inhibiting the secretion of IL-17 and the recruitment of neutrophils and serving as a barrier against the development of autoimmune kidney disease (Donadei et al., 2019). So the role of adaptive immunity cannot be neglected and merits further exploration in subsequent researches.

Mo et al. (2020) reported that miR-21 was upregulated in Nano-Ni-induced pulmonary fibrosis, and knocking out miR-21 ameliorated pulmonary inflammation significantly. Nurrahmah et al. (2021) also revealed that Retinoic acid abrogates LPS-induced inflammatory response via negative regulation of miR-21 and NF-κB signaling pathway. Our results revealed that miR-21-5p was significantly elevated and SPRY1/ERK/NF-κB pathway was activated in kidney tissue of mice in UUO group. SPRY1, a direct target of miR-21, inhibits the ERK/NF-κB pathway, which is an inflammation-related signaling pathways (White et al., 2020). Moreover, the low dose rhEPO treatment in the UUO + EPO group could decrease the miR-21-5p expression and reverse the activation of SPRY1/ERK/NF-κB signaling pathway. Therefore, we propose that EPO maybe inhibit the inflammatory and immune response of obstructive kidneys by negatively regulating miR-21-5p and its downstream SPRY1/ERK/NF-κB pathways, thereby inhibiting renal fibrosis of mice.

In summary, our research showed that 7-day low dose rhEPO treatment could significantly alleviate cortical inflammation and fibrosis in the obstructive kidneys of mice. Additionally, both *in vivo* and *in vitro* studies demonstrated that rhEPO treatment could reverse the upregulation of miR-21-5p and the activation of the SPRY1/ERK/NF-κB pathway. These results indicate that EPO probably exerts anti-inflammatory and anti-fibrotic effects in obstructive renal fibrosis through miR-21-5p/SPRY1/ERK/NF-kB axis. Our conclusion helps to understand the underlying mechanisms of EPO in the renal fibrosis treatment.

However, our study still has limitations. In clinical practice, EPO therapy is associated with certain side effects, such as hypertension and thrombosis (Möllmann, 1992), which have limited its translational application to a certain extent. Existing studies have confirmed that non-hematopoietic EPO analogs can minimize the hematopoietic-related side effects of natural EPO to the greatest extent and fully exert its anti-inflammatory effects (Sugimoto and Goda, 2025) as well as tissue protective effects against hypoxic stress (Cho et al., 2022), which points out the direction for subsequent translational development. As the most commonly used animal model in renal fibrosis research, the UUO model is characterized by stability, rapidity, and maturity. However, it still has certain limitations and cannot fully simulate the disease progression of CKD, which is not conducive to the in-depth exploration of the disease mechanism. Therefore, it is still necessary to explore and establish more suitable animal models for renal fibrosis research. Although we have revealed that EPO can reduce the expression of miR-21-5p, which is in line with the findings of a previous study (Chen et al., 2015), there is a lack of functional gain/loss experiments of miR-21-5p in the primary disease model, and we have not yet confirmed whether EPO regulates miR-21 directly. We believe that the anti-fibrotic effect of EPO is closely associated with innate immunity, while it remains unclear which specific immune cells it acts upon. In our ongoing follow-up studies, we will continue this line of research, further explore the specific manner by which EPO affects miR-21 expression, and investigate the in-depth anti-fibrotic immune mechanism of EPO in obstructive renal fibrosis.

## Data Availability

The original contributions presented in the study are included in the article/Supplementary Material, further inquiries can be directed to the corresponding author.
