# Supplementary figures and images for: Erythropoietin alleviate obstructive renal fibrosis by regulating immunity and inflammation through miR-21-5p/SPRY1/ERK1/2/NF-κB pathway inhibition
Source: Front Mol Biosci. 2026 Mar 2;13:1795772. doi: 10.3389/fmolb.2026.1795772 (PMC12989493; doi:10.3389/fmolb.2026.1795772)

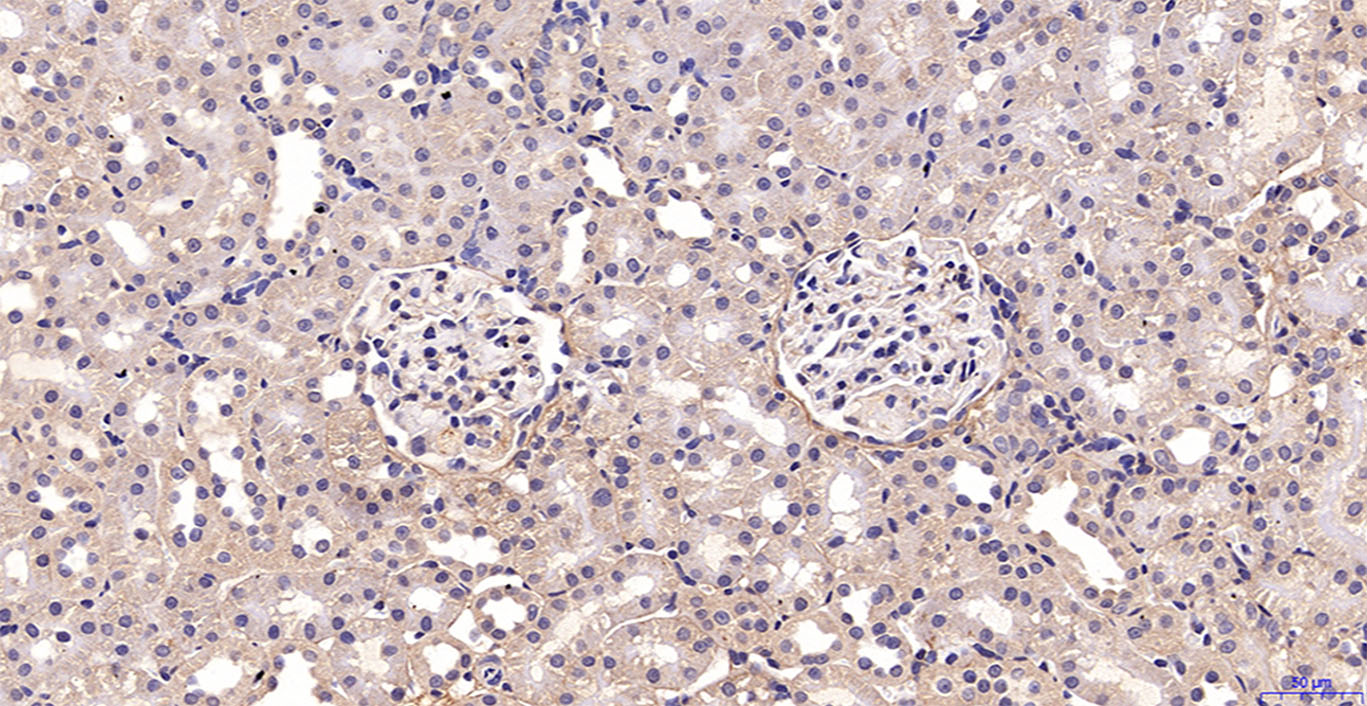

Supplement: Supplementary file 1 [file DataSheet3.zip › Supplementary Figure 2 E/SHAM Collagen I.jpg]

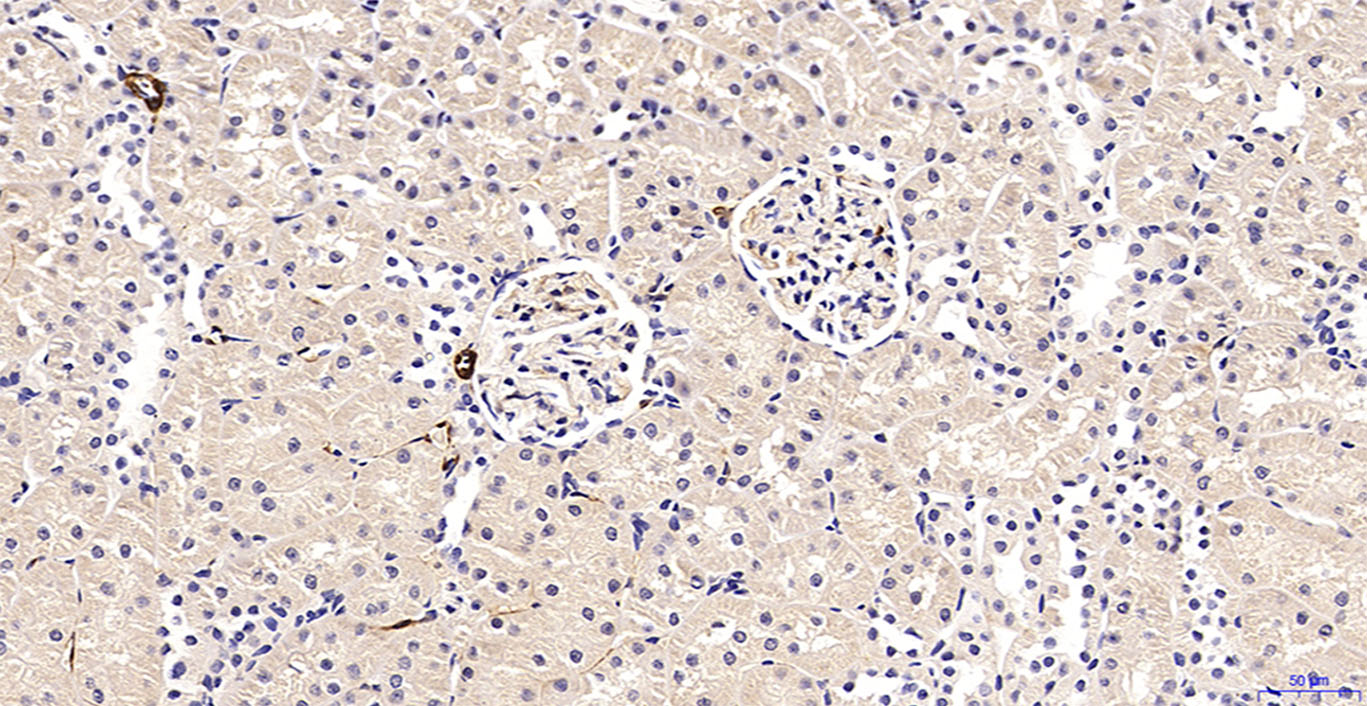

Supplement: Supplementary file 1 [file DataSheet3.zip › Supplementary Figure 2 E/SHAM α-SMA.jpg]

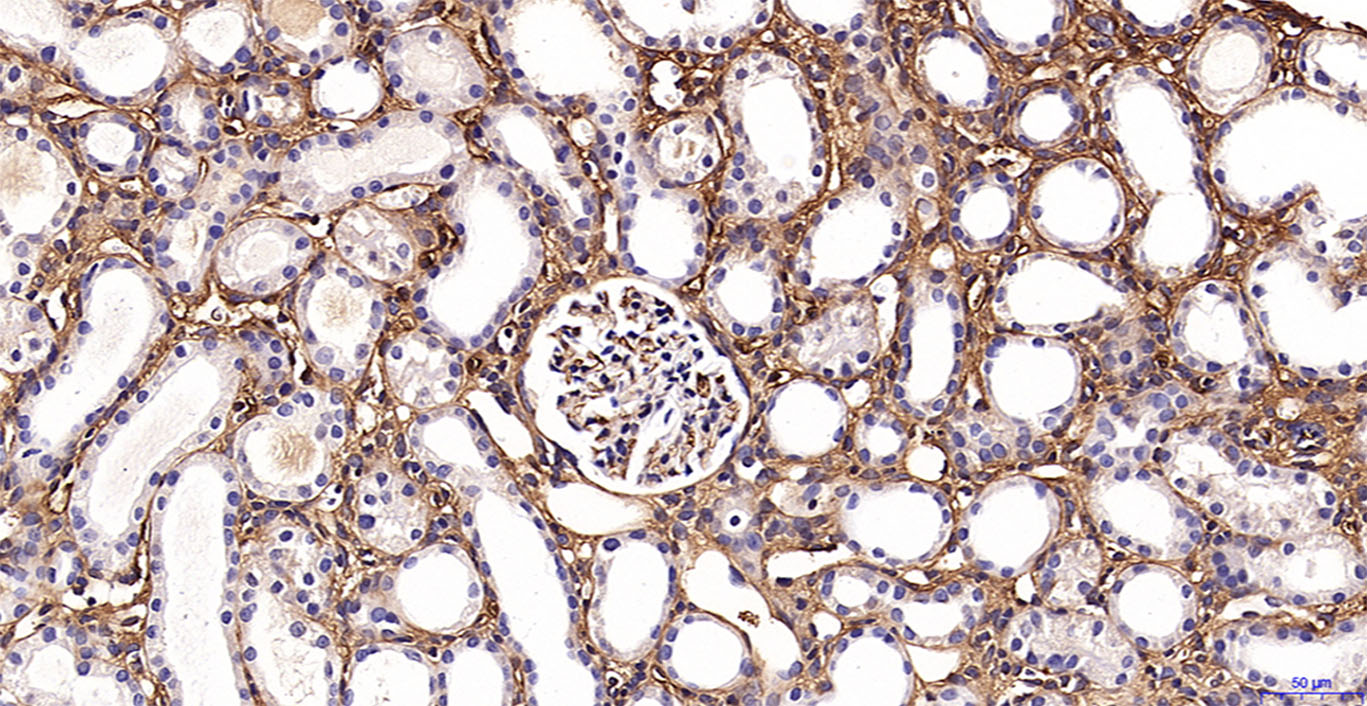

Supplement: Supplementary file 1 [file DataSheet3.zip › Supplementary Figure 2 E/UUO Collagen I.jpg]

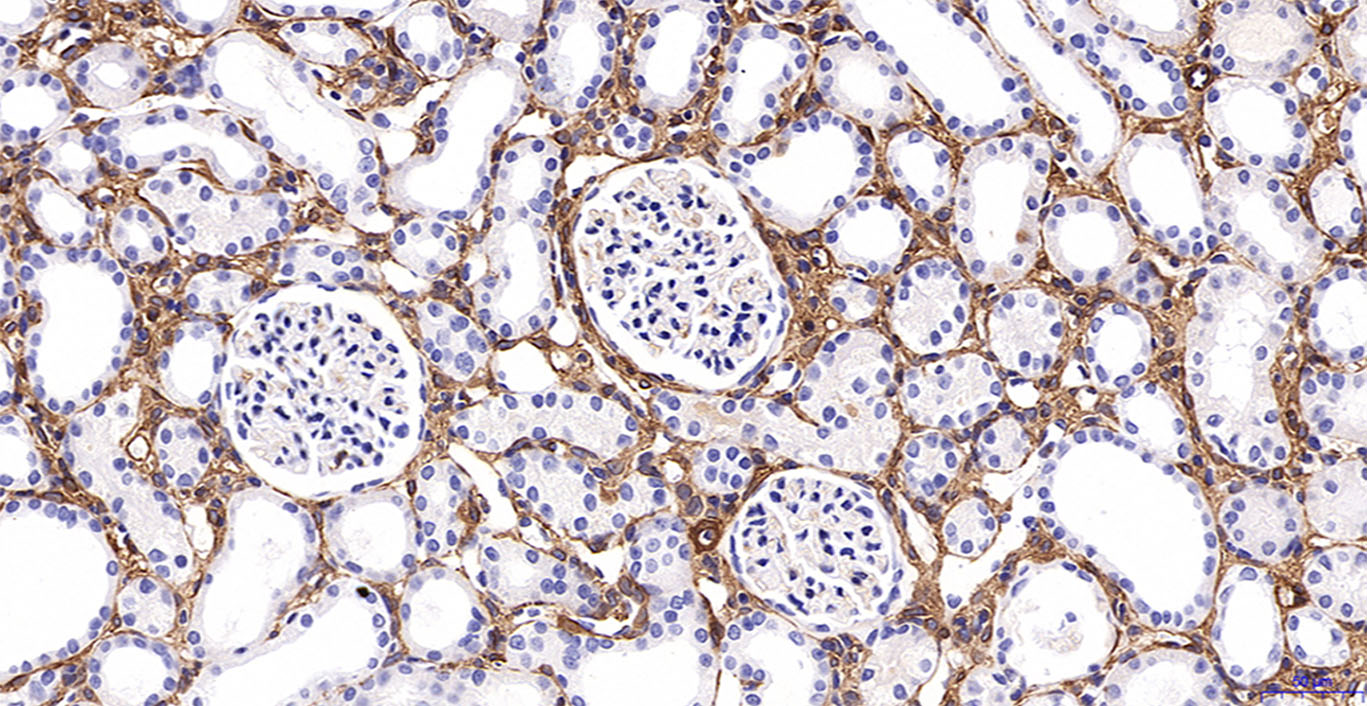

Supplement: Supplementary file 1 [file DataSheet3.zip › Supplementary Figure 2 E/UUO α-SMA.jpg]

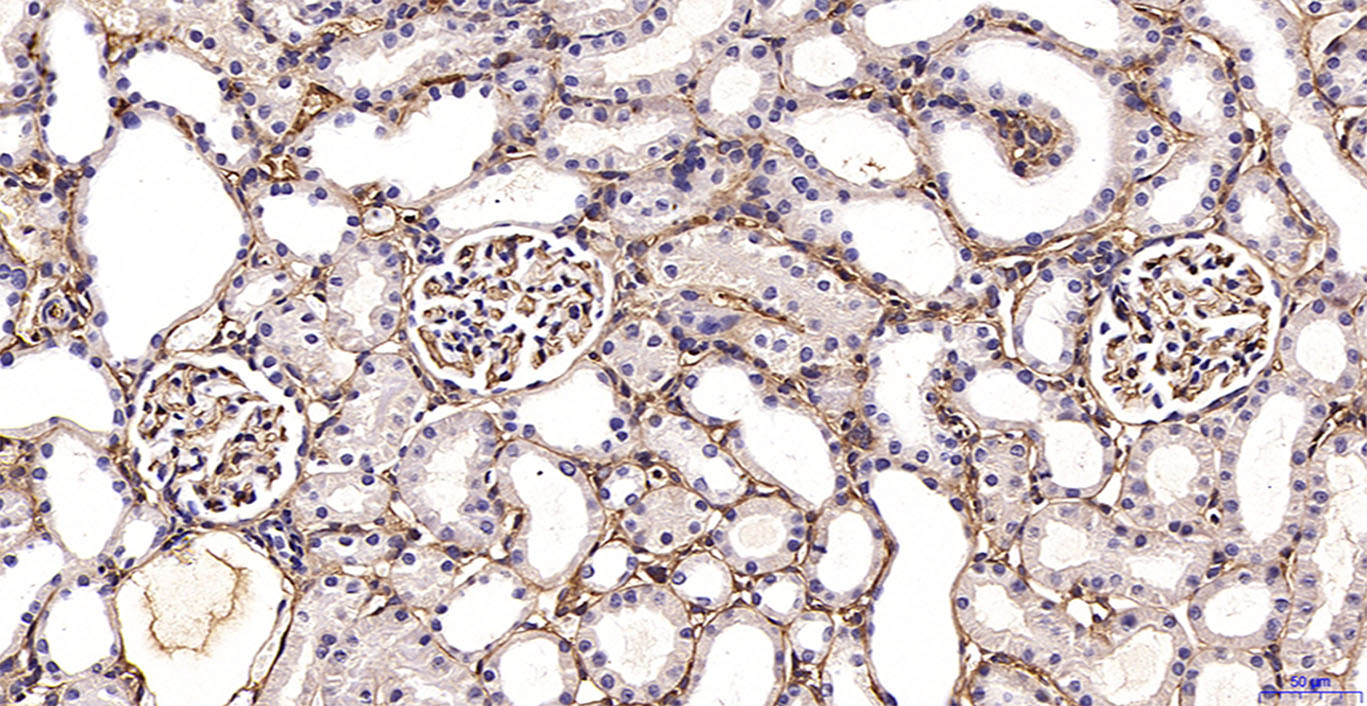

Supplement: Supplementary file 1 [file DataSheet3.zip › Supplementary Figure 2 E/UUO+EPO Collagen I.jpg]

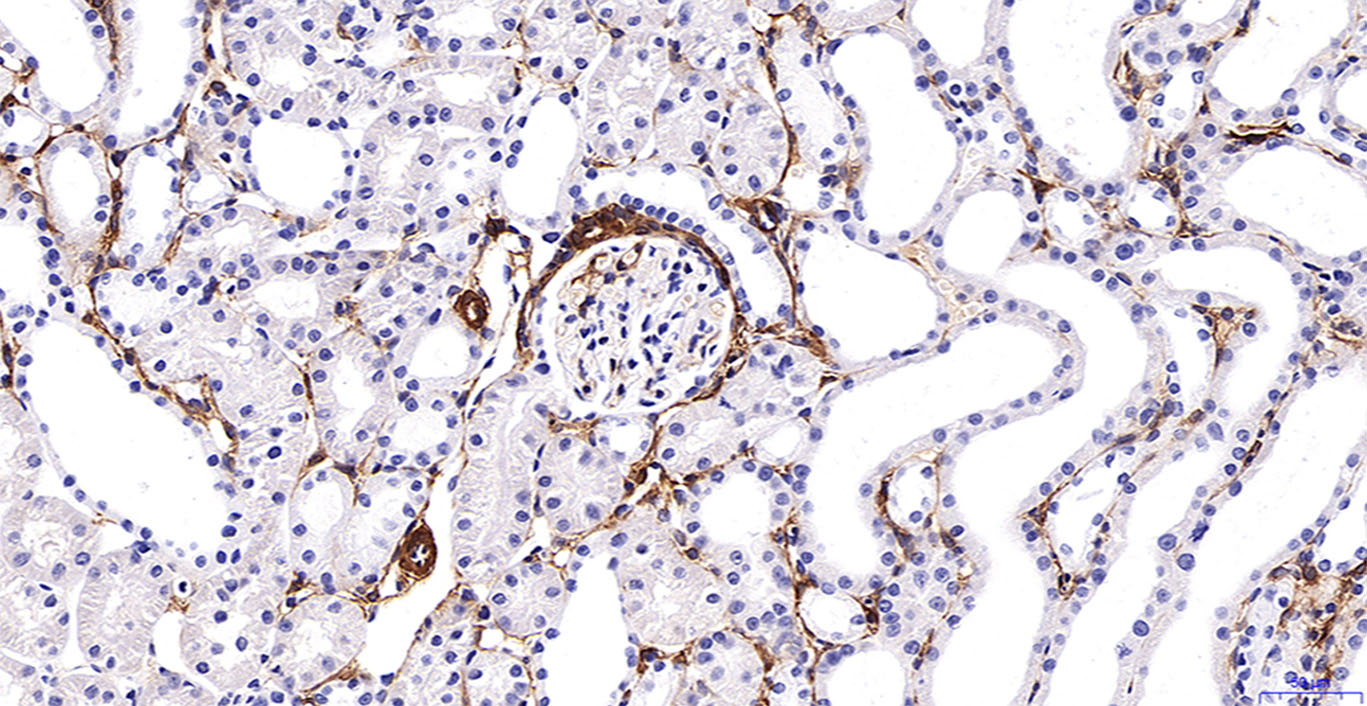

Supplement: Supplementary file 1 [file DataSheet3.zip › Supplementary Figure 2 E/UUO+EPO α-SMA.jpg]

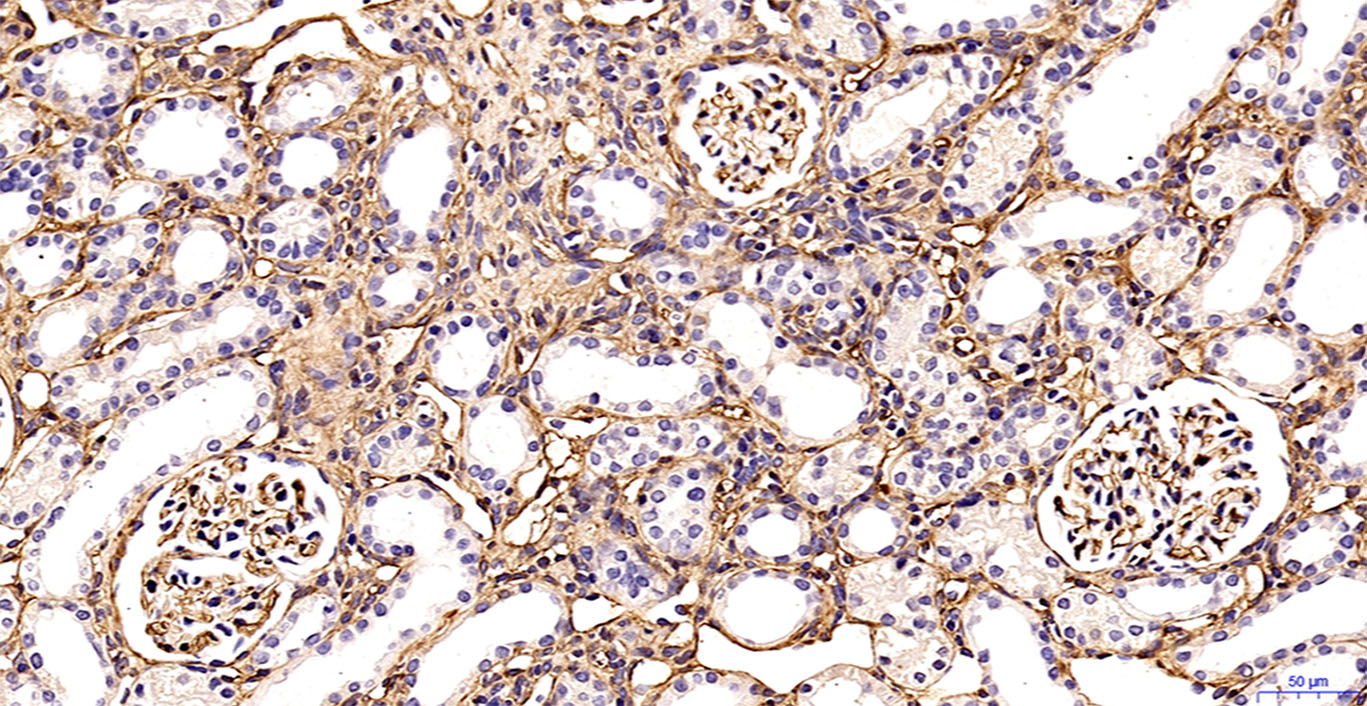

Supplement: Supplementary file 1 [file DataSheet3.zip › Supplementary Figure 2 E/UUO+saline Collagen I.jpg]

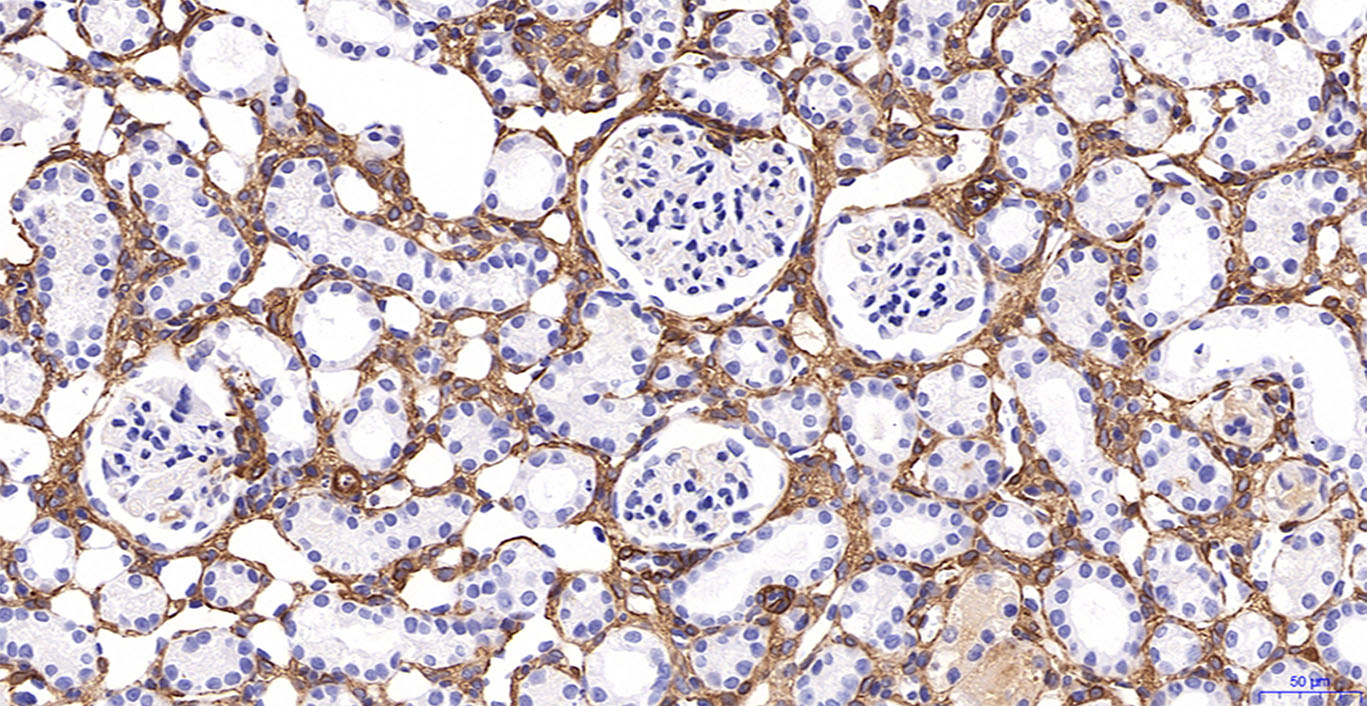

Supplement: Supplementary file 1 [file DataSheet3.zip › Supplementary Figure 2 E/UUO+saline α-SMA.jpg]

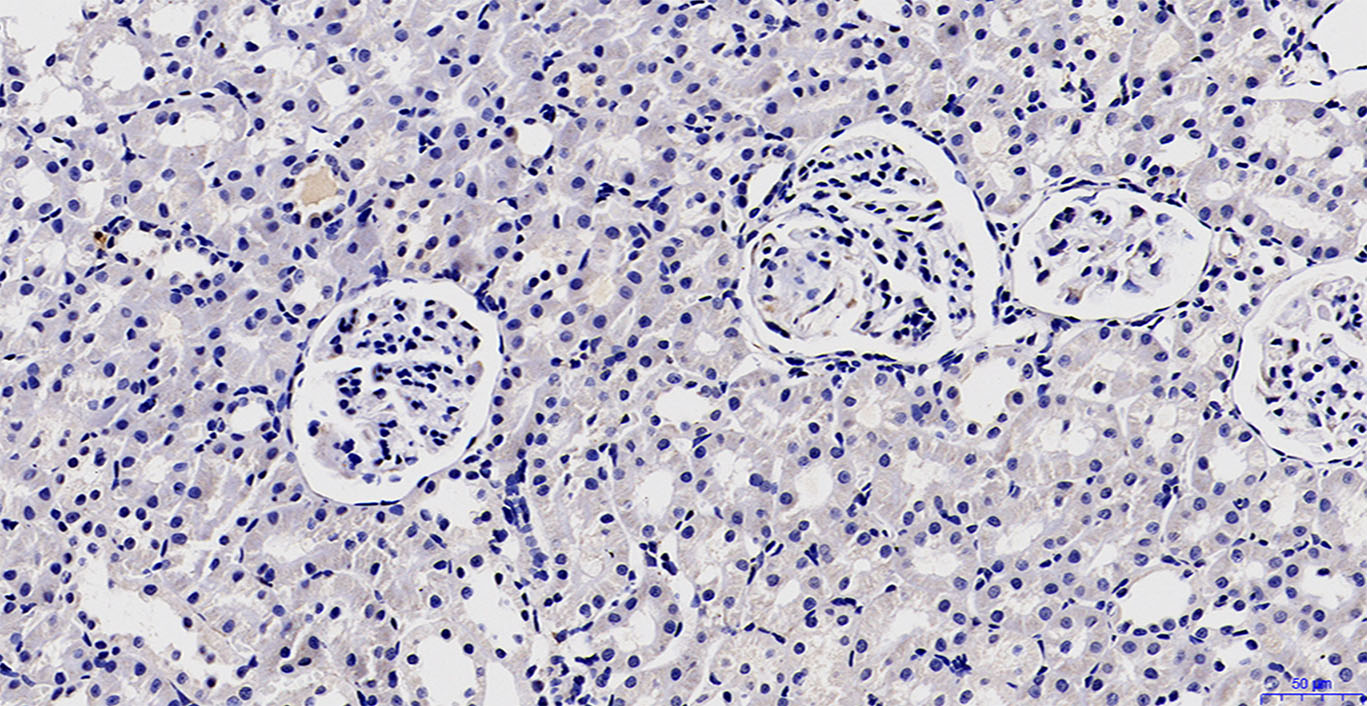

Supplement: Supplementary file 2 [file DataSheet4.zip › Supplementary Figure 4 A/SHAM IL-6.jpg]

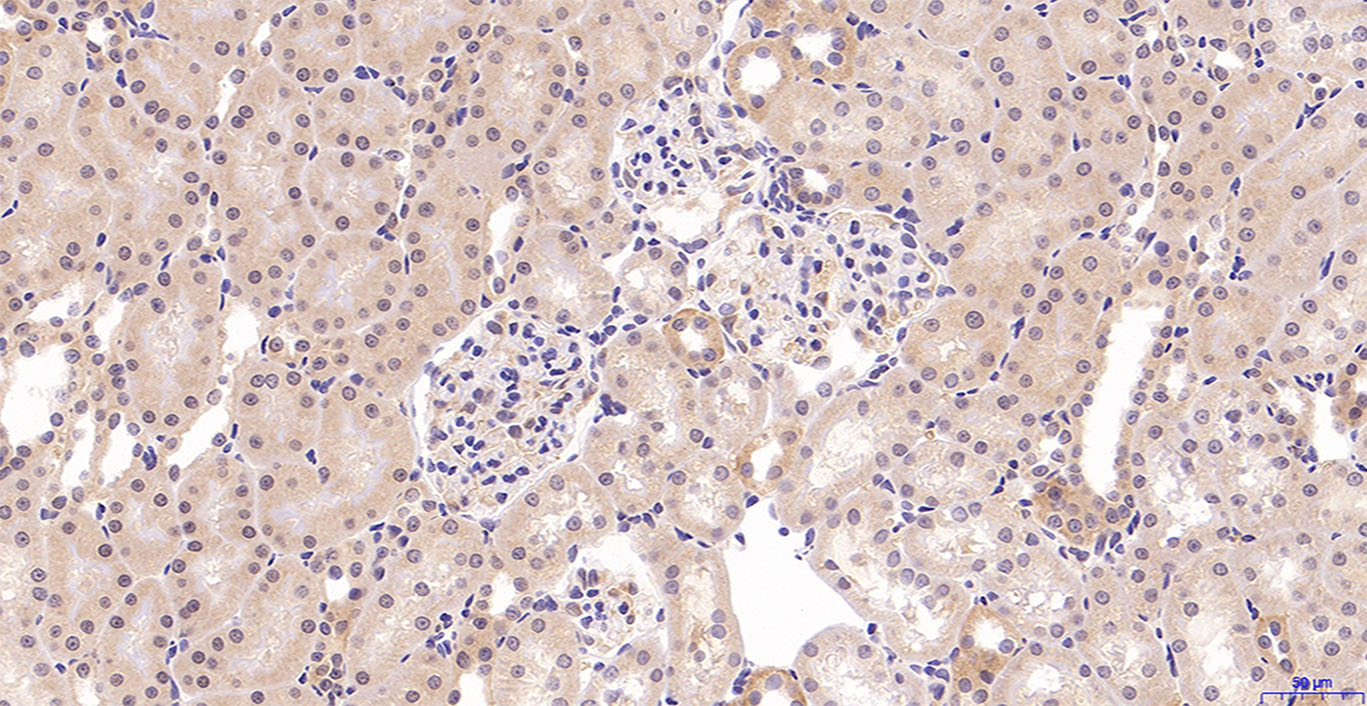

Supplement: Supplementary file 2 [file DataSheet4.zip › Supplementary Figure 4 A/SHAM MPO.jpg]

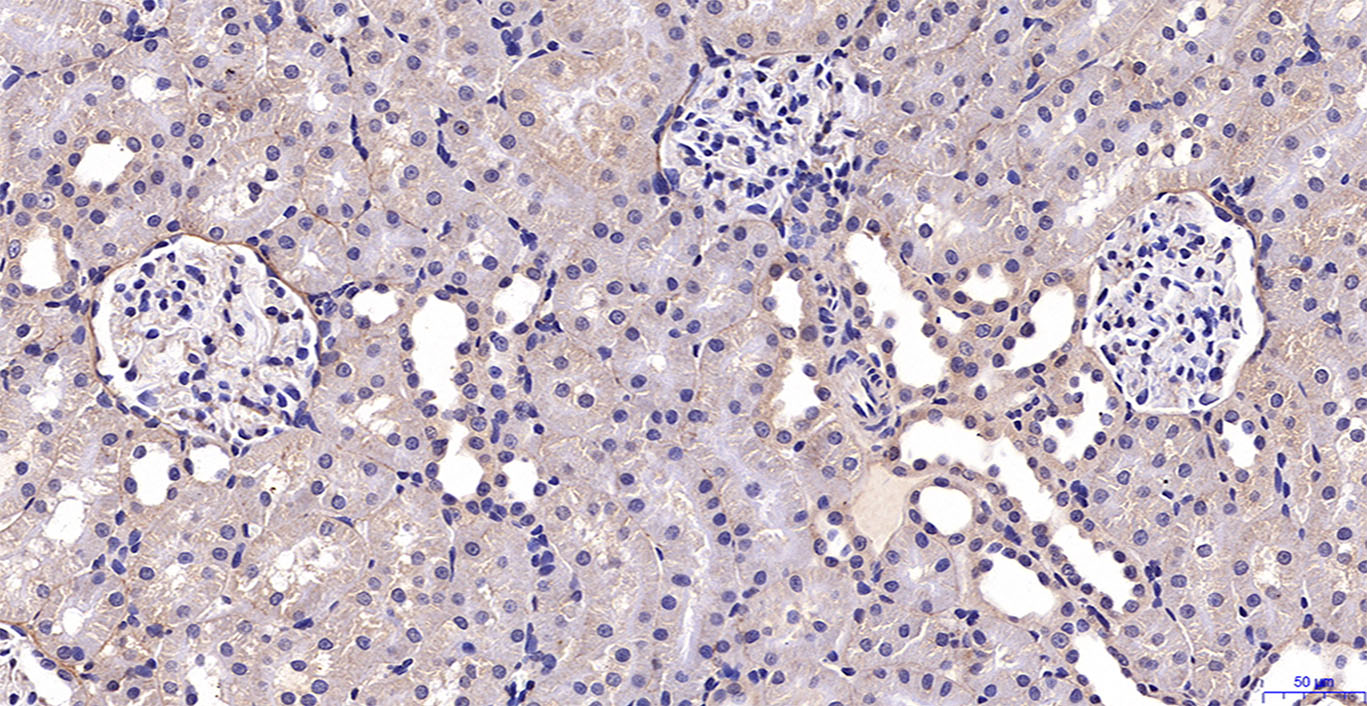

Supplement: Supplementary file 2 [file DataSheet4.zip › Supplementary Figure 4 A/SHAM TNF-α.jpg]

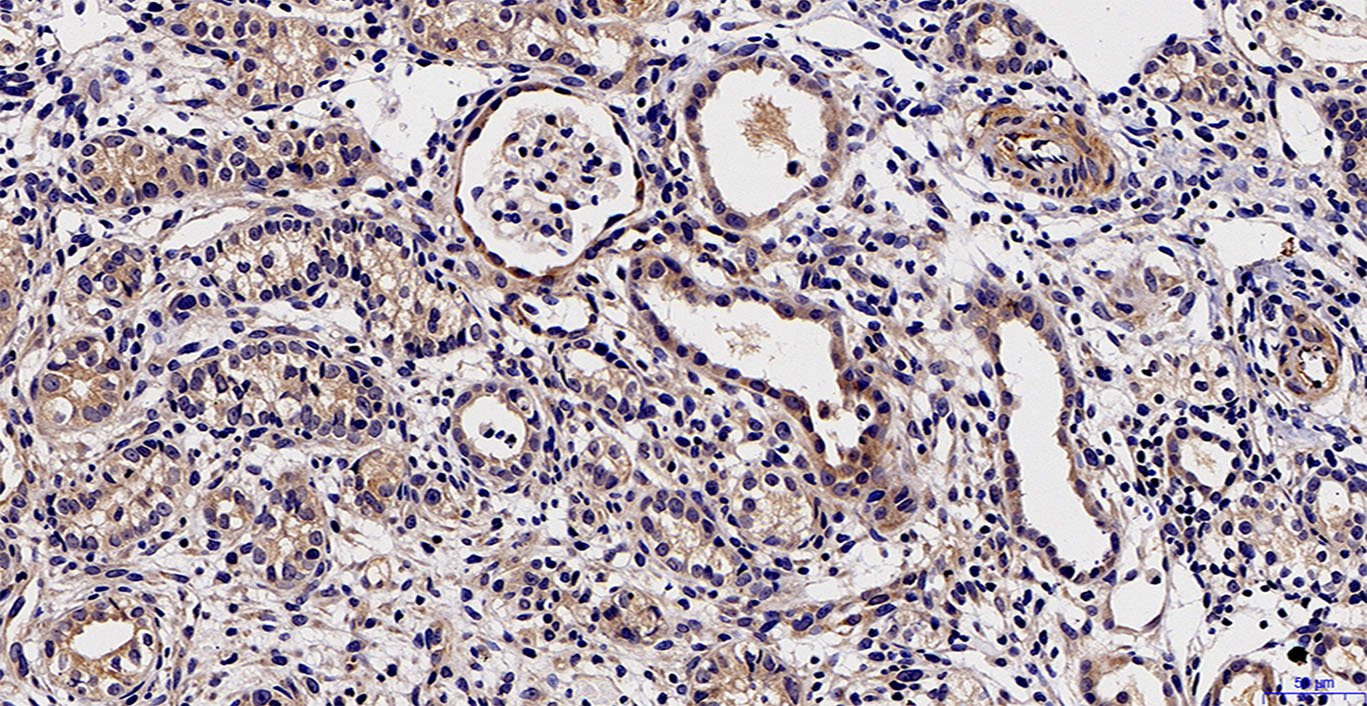

Supplement: Supplementary file 2 [file DataSheet4.zip › Supplementary Figure 4 A/UUO IL-6.jpg]

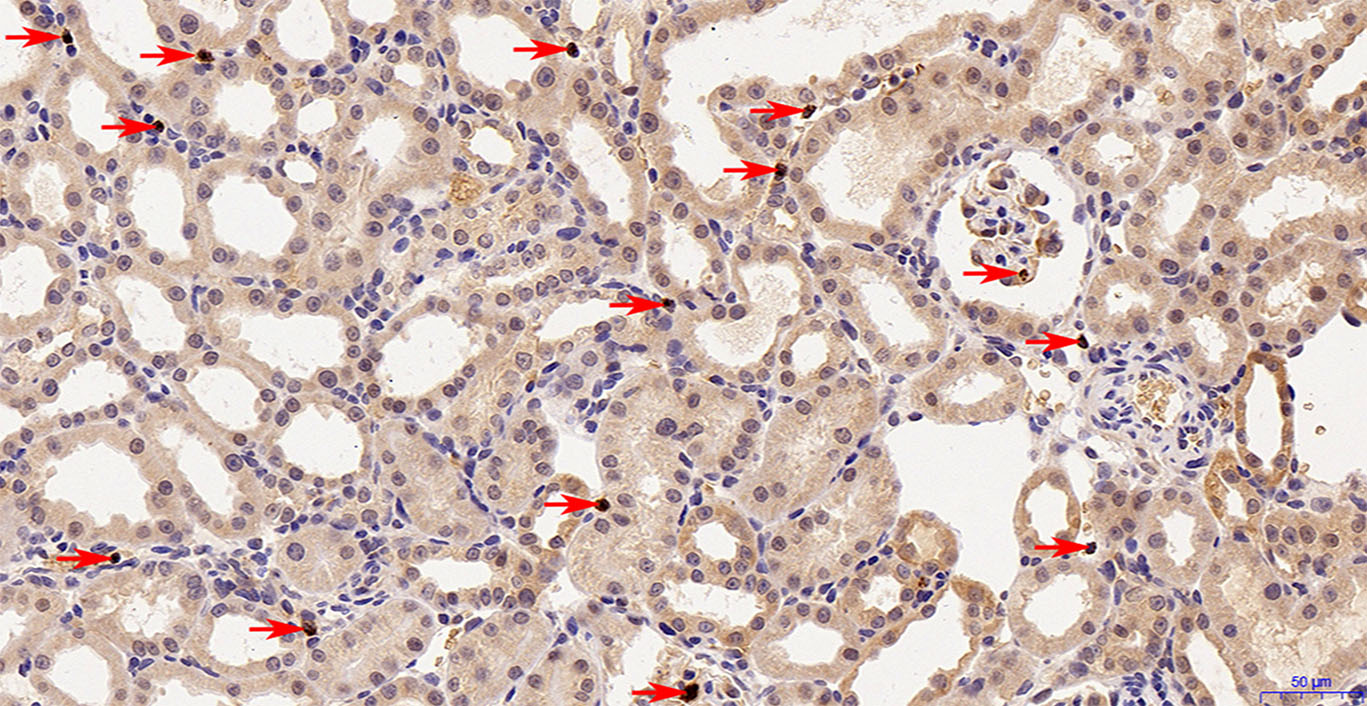

Supplement: Supplementary file 2 [file DataSheet4.zip › Supplementary Figure 4 A/UUO MPO.jpg]

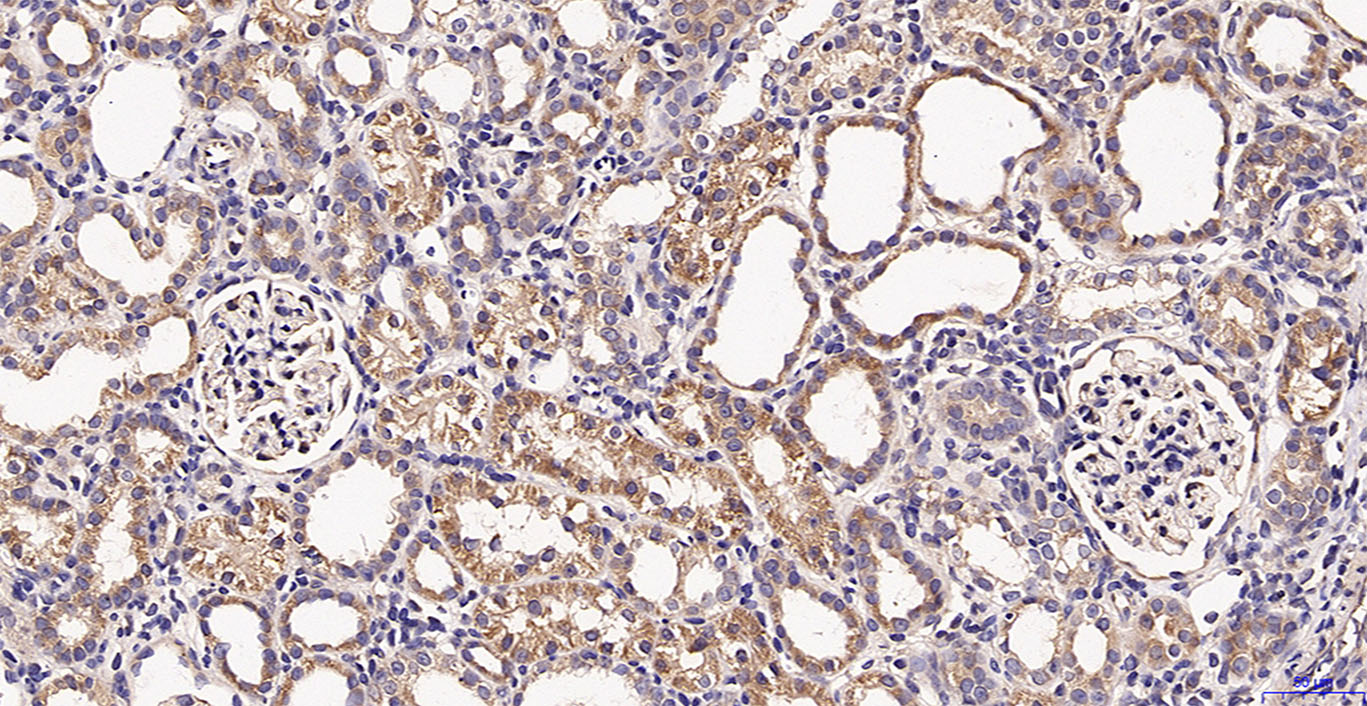

Supplement: Supplementary file 2 [file DataSheet4.zip › Supplementary Figure 4 A/UUO TNF-α.jpg]

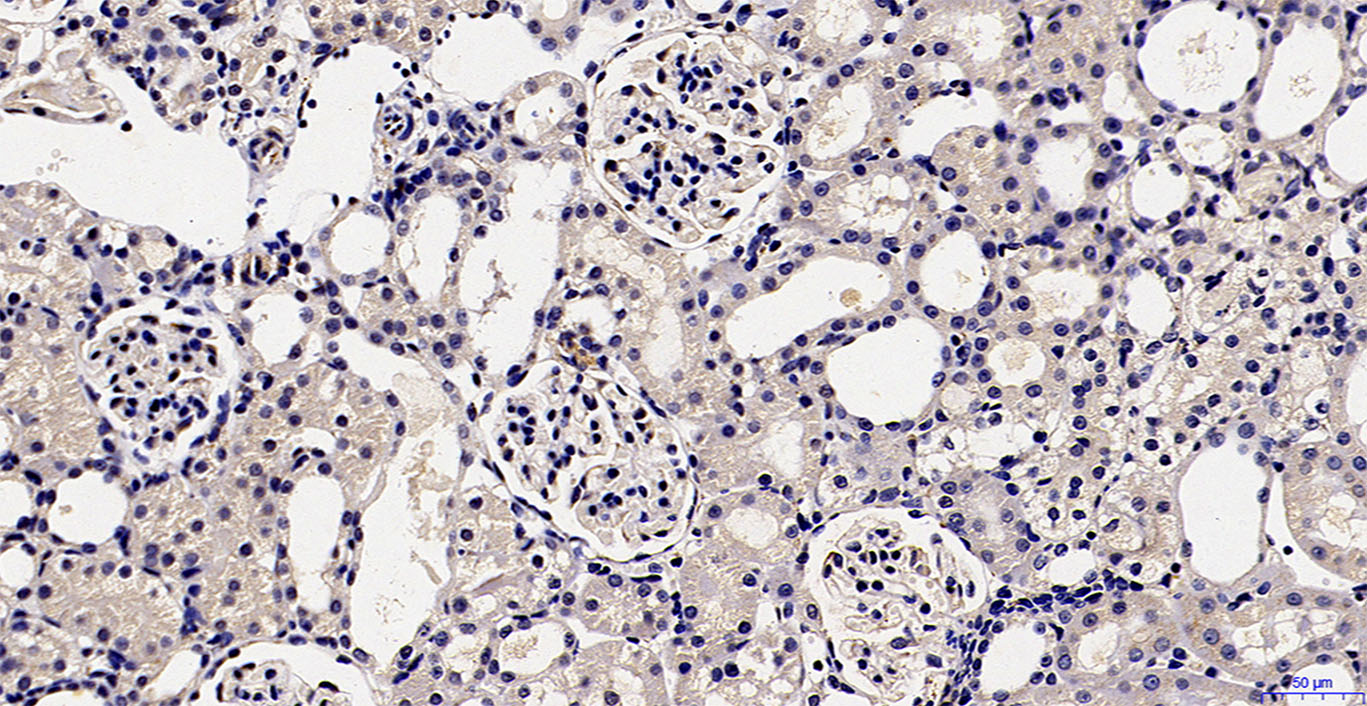

Supplement: Supplementary file 2 [file DataSheet4.zip › Supplementary Figure 4 A/UUO+EPO IL-6.jpg]

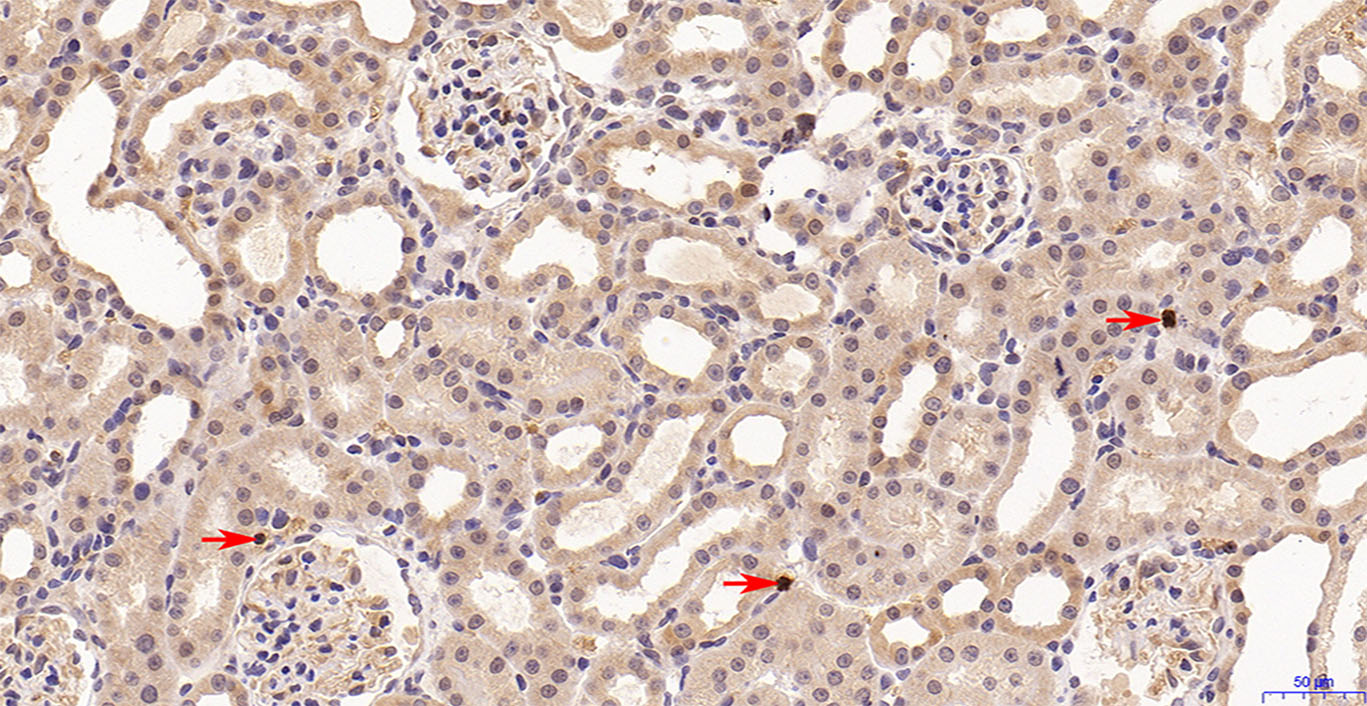

Supplement: Supplementary file 2 [file DataSheet4.zip › Supplementary Figure 4 A/UUO+EPO MPO.jpg]

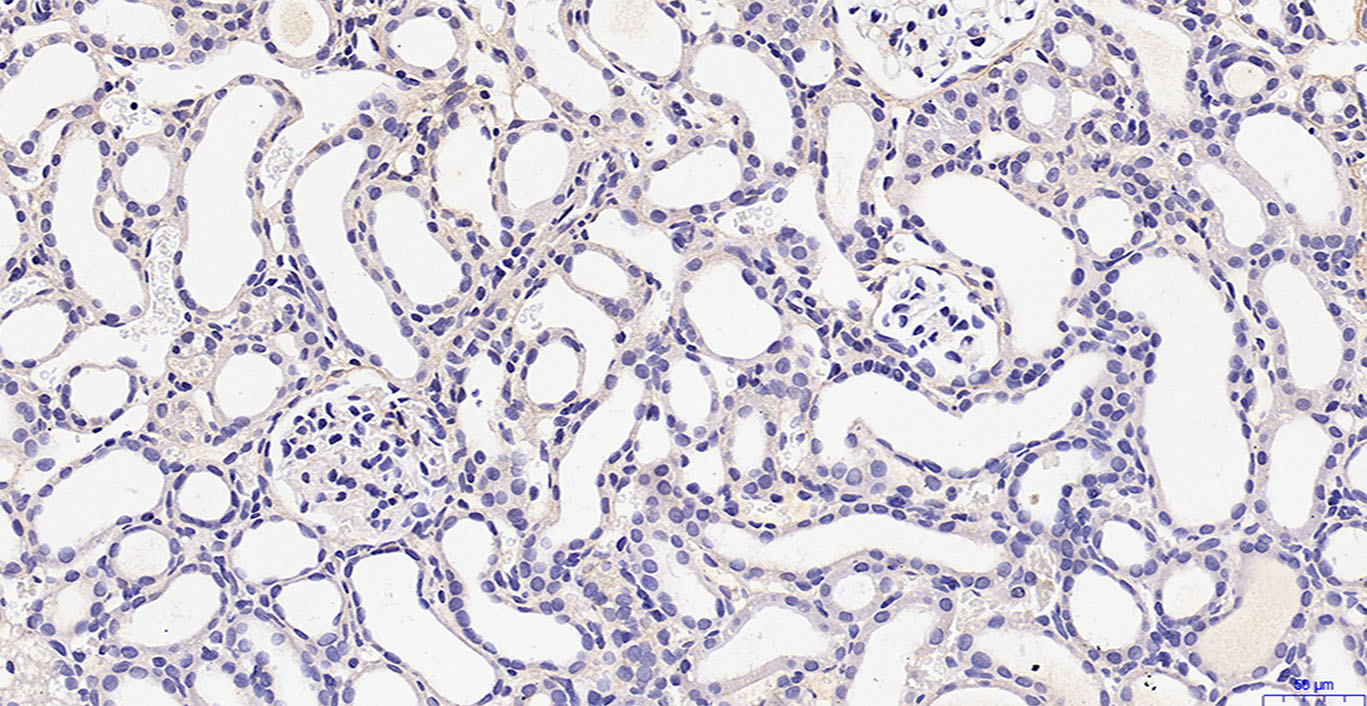

Supplement: Supplementary file 2 [file DataSheet4.zip › Supplementary Figure 4 A/UUO+EPO TNF-α.jpg]

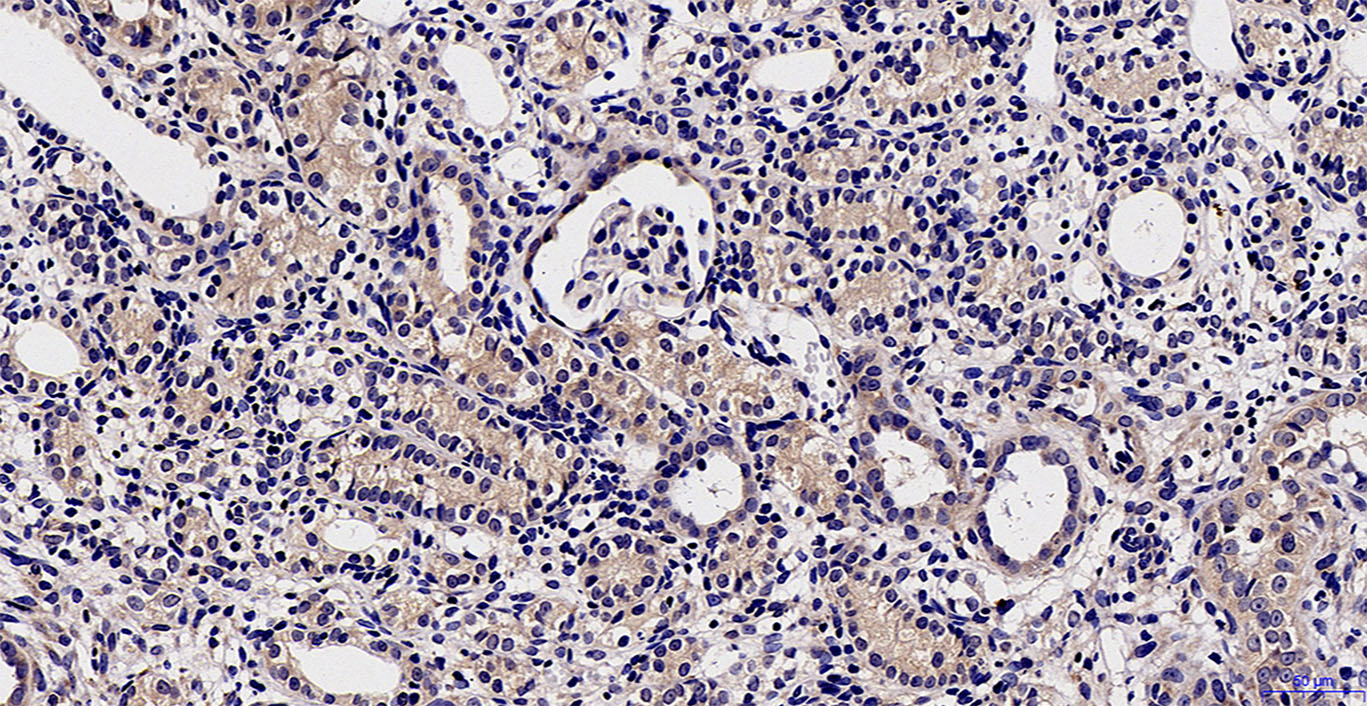

Supplement: Supplementary file 2 [file DataSheet4.zip › Supplementary Figure 4 A/UUO+saline IL-6.jpg]

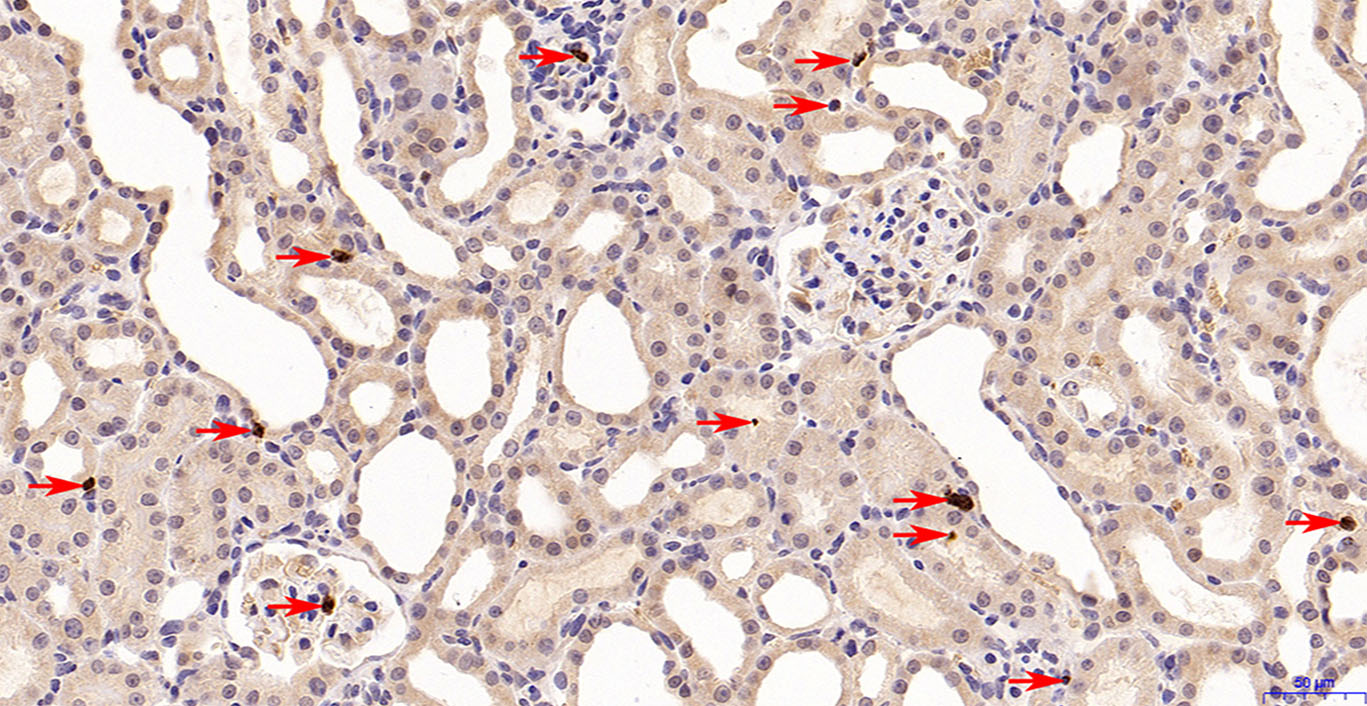

Supplement: Supplementary file 2 [file DataSheet4.zip › Supplementary Figure 4 A/UUO+saline MPO.jpg]

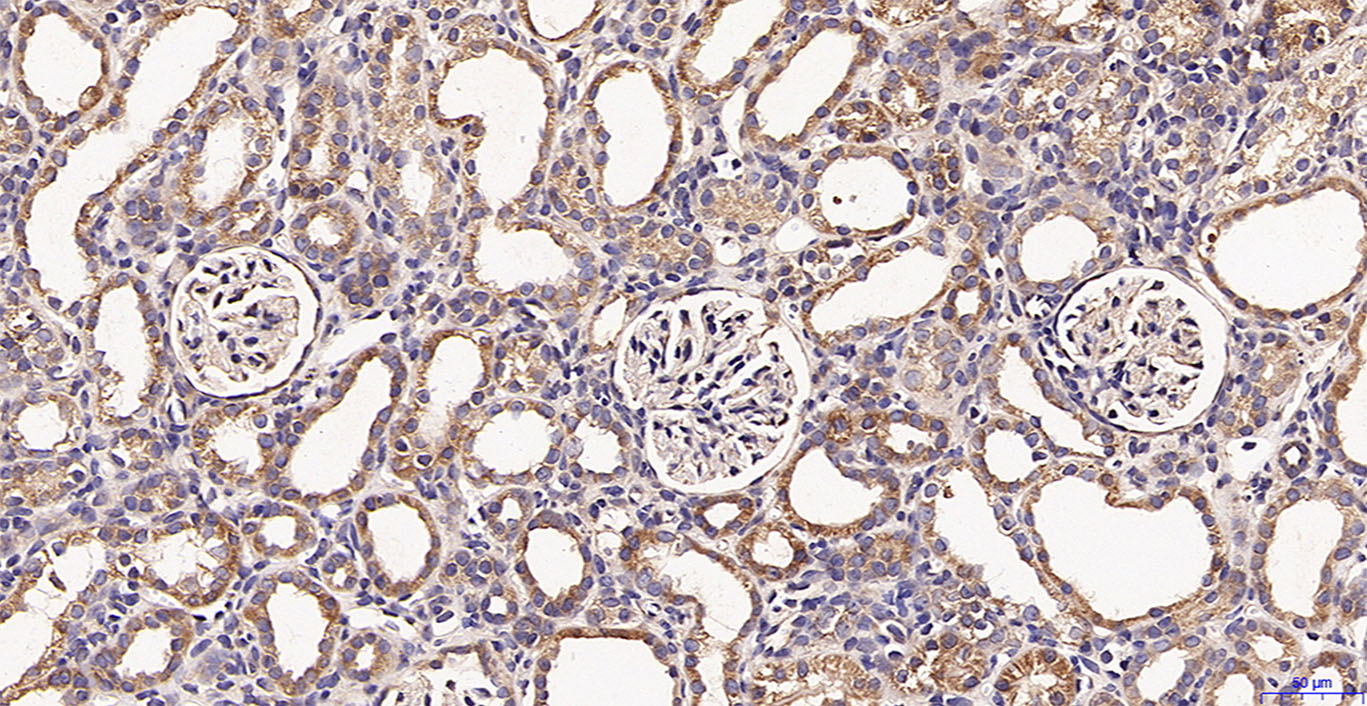

Supplement: Supplementary file 2 [file DataSheet4.zip › Supplementary Figure 4 A/UUO+saline TNF-α.jpg]

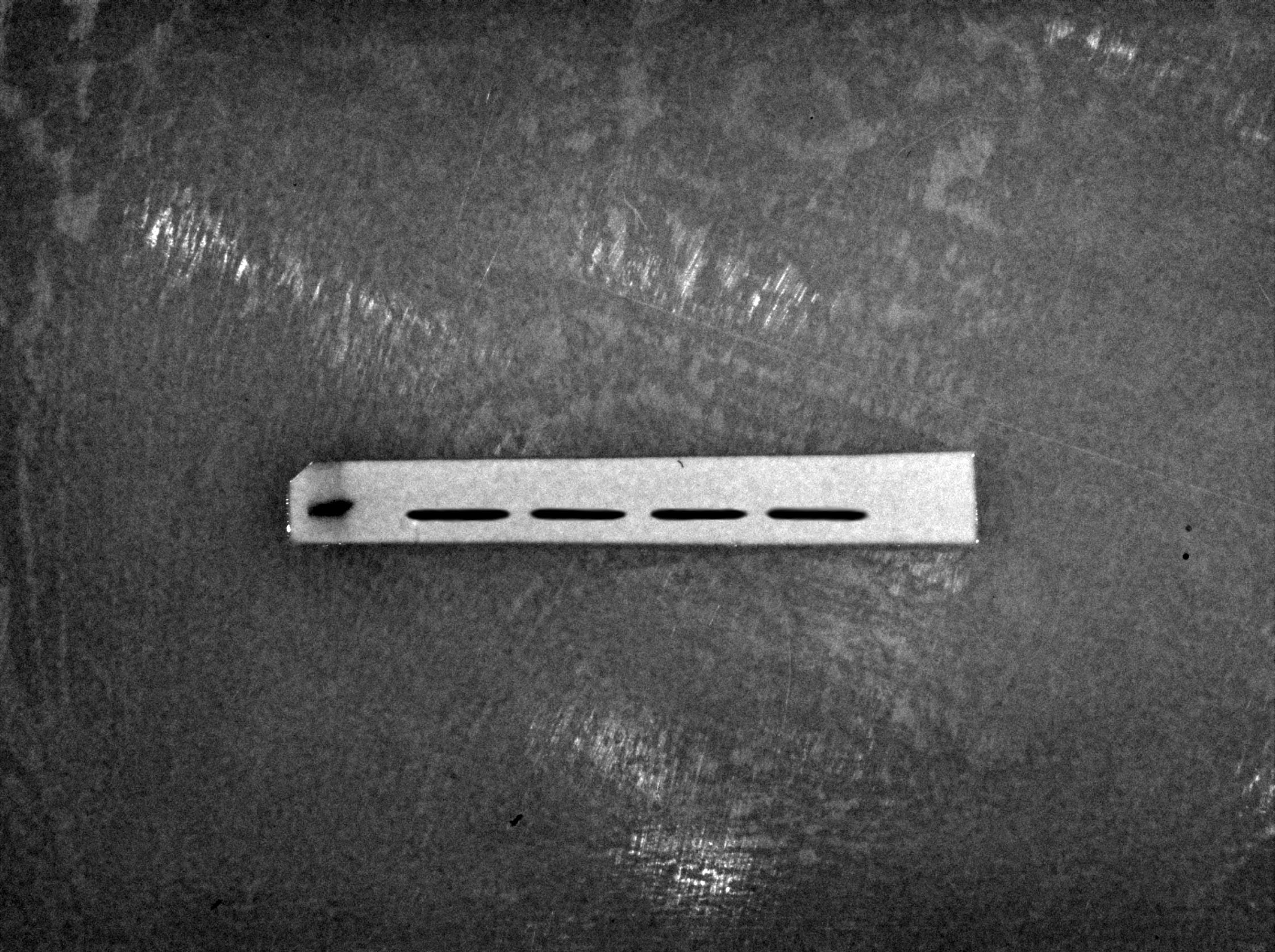

Supplement: Supplementary file 3 [file DataSheet1.zip › WB Supplementary/Supplementary Fig-2 C actin.jpg]

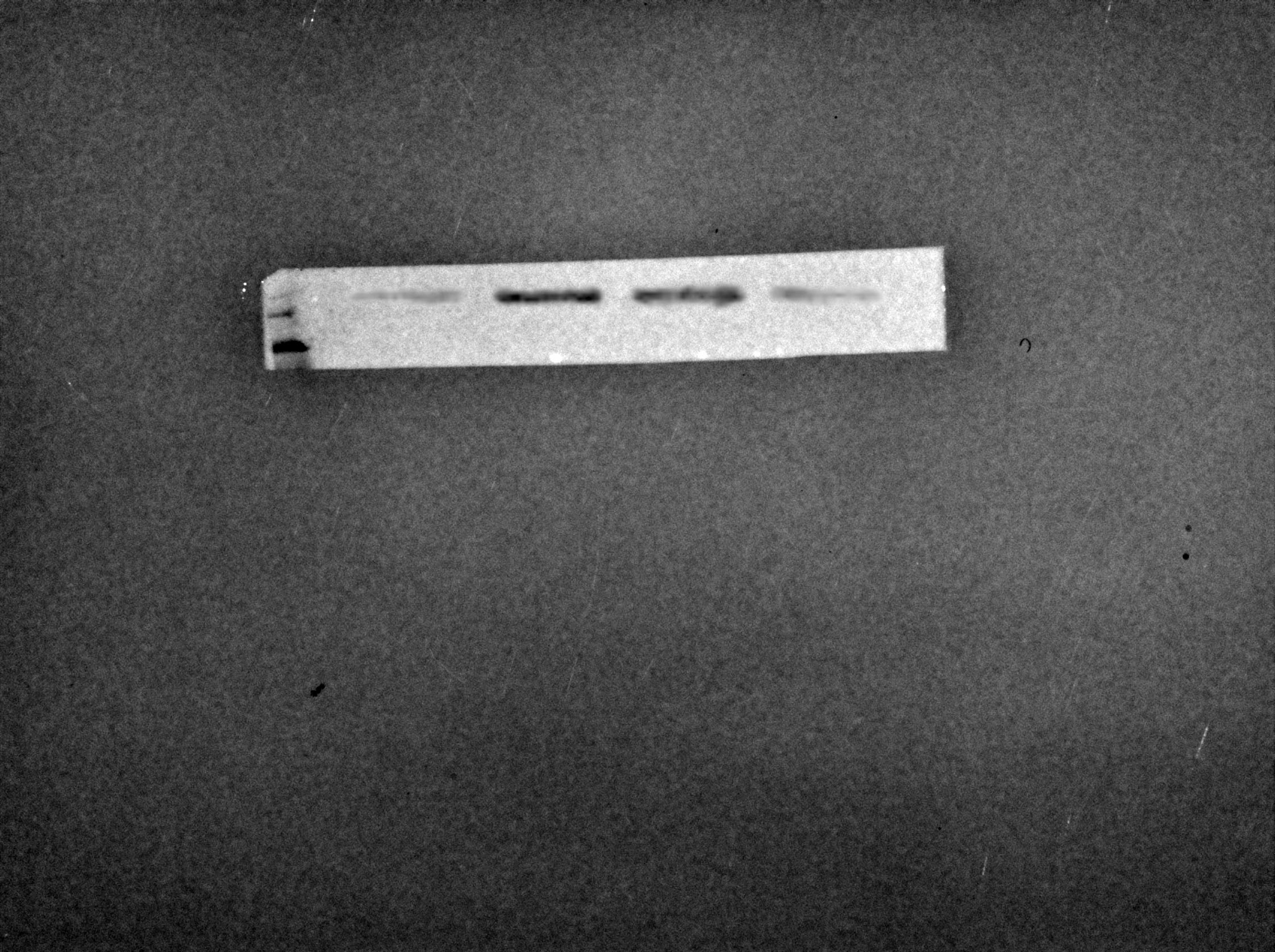

Supplement: Supplementary file 3 [file DataSheet1.zip › WB Supplementary/Supplementary Fig-2 C collagen I.jpg]

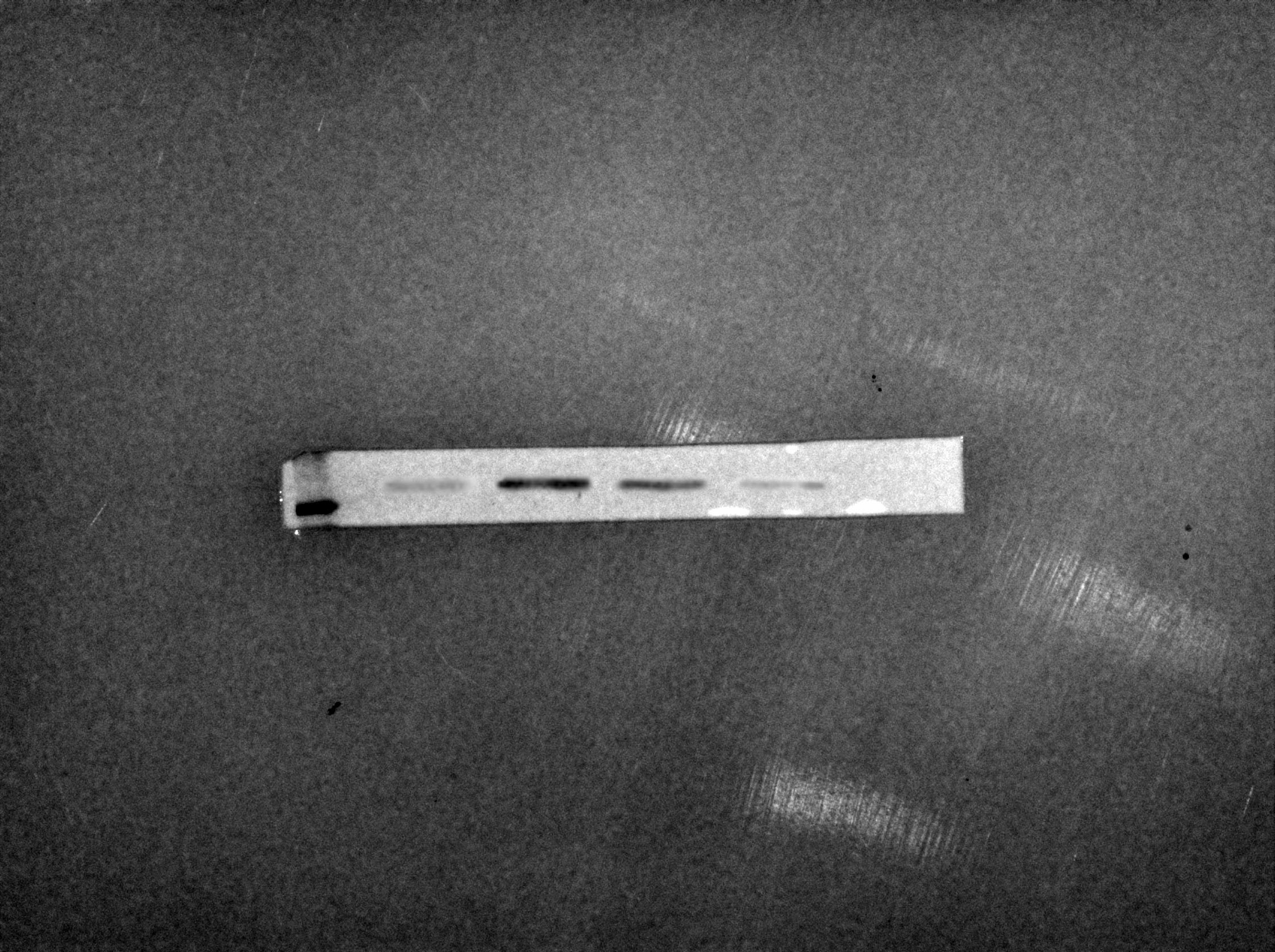

Supplement: Supplementary file 3 [file DataSheet1.zip › WB Supplementary/Supplementary Fig-2 C α-SMA.jpg]

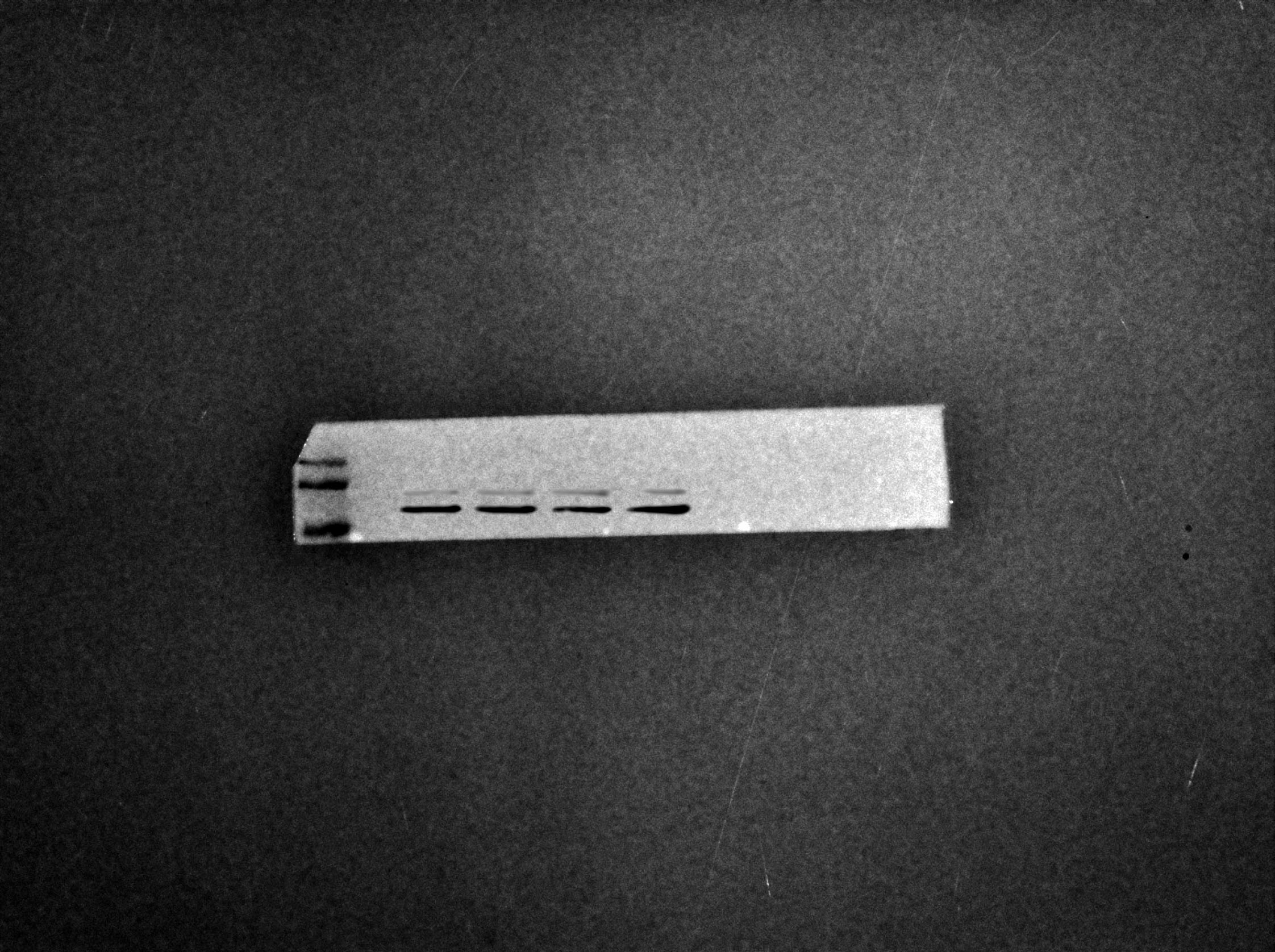

Supplement: Supplementary file 3 [file DataSheet1.zip › WB Supplementary/Supplementary Fig-3 B ERK.jpg]

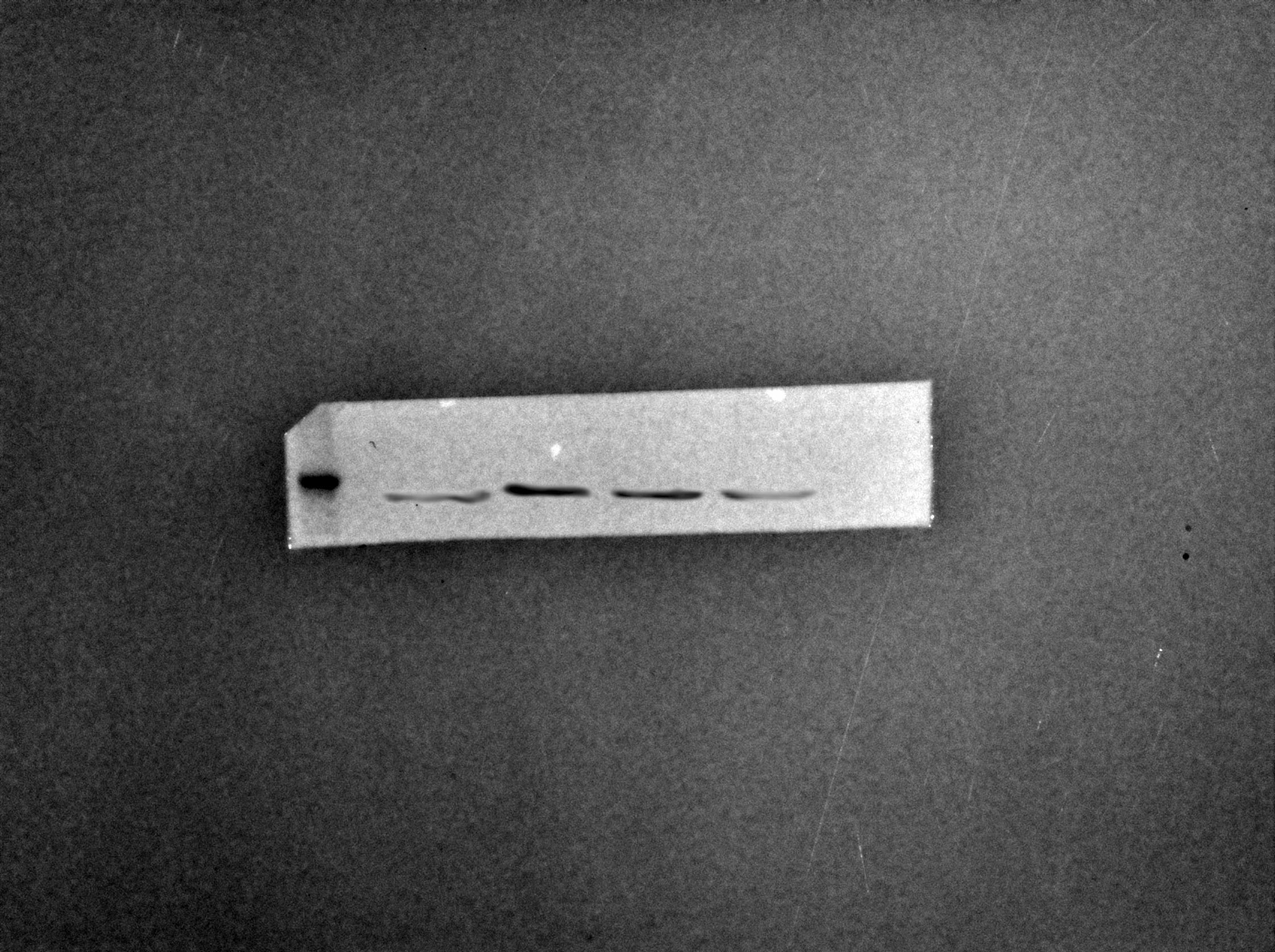

Supplement: Supplementary file 3 [file DataSheet1.zip › WB Supplementary/Supplementary Fig-3 B IL-6.jpg]

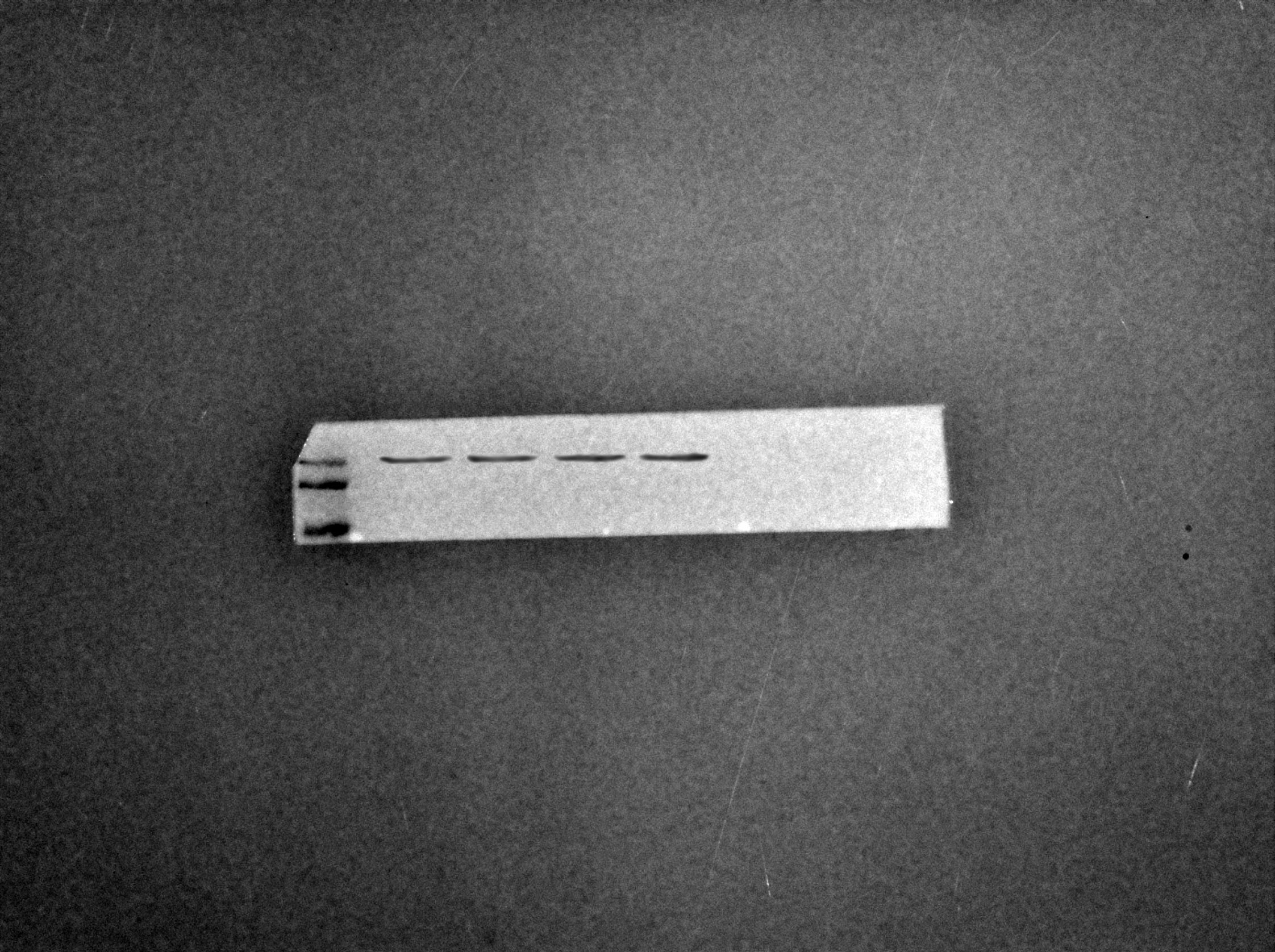

Supplement: Supplementary file 3 [file DataSheet1.zip › WB Supplementary/Supplementary Fig-3 B NF-κB.jpg]

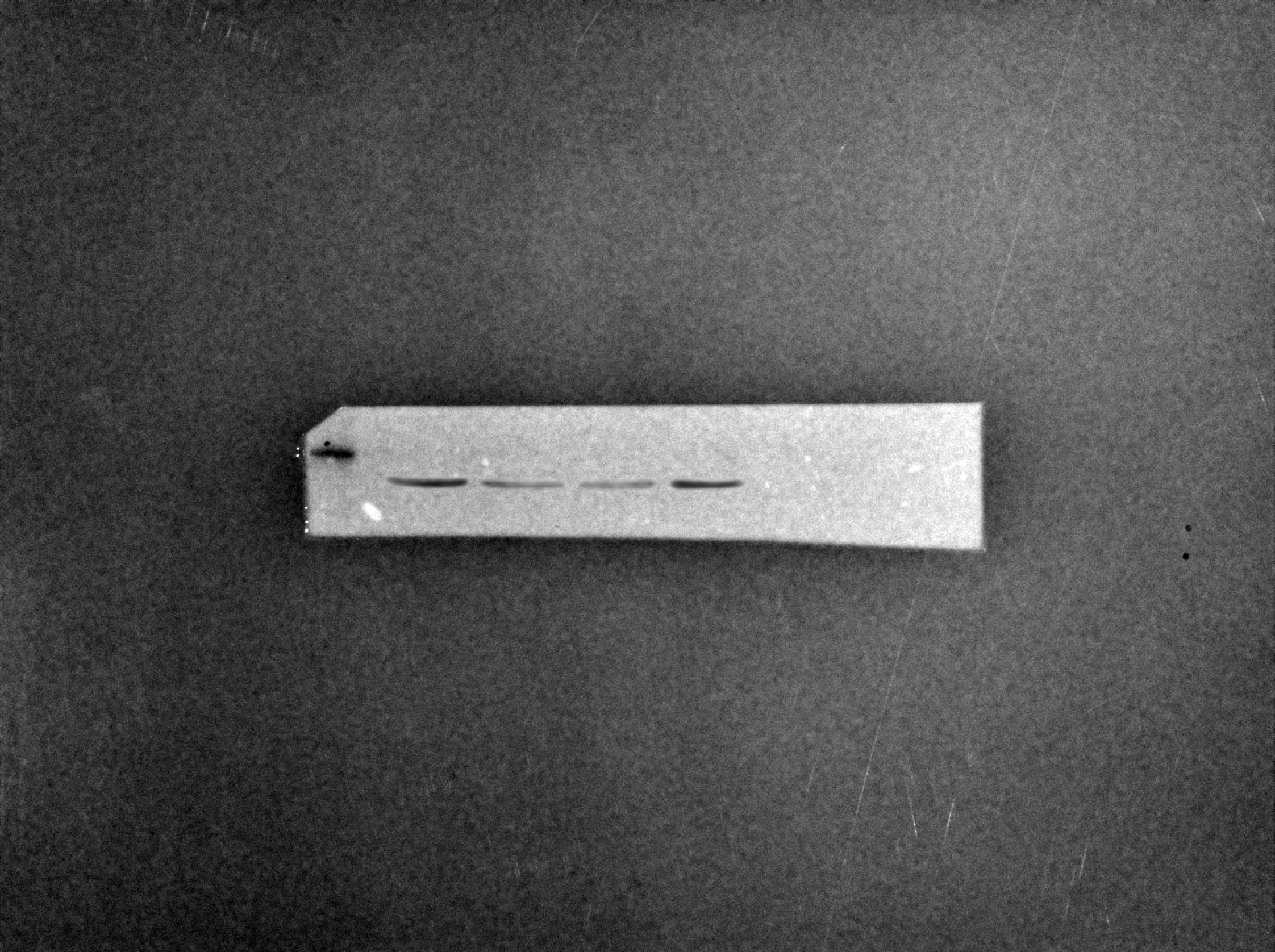

Supplement: Supplementary file 3 [file DataSheet1.zip › WB Supplementary/Supplementary Fig-3 B SPRY1.jpg]

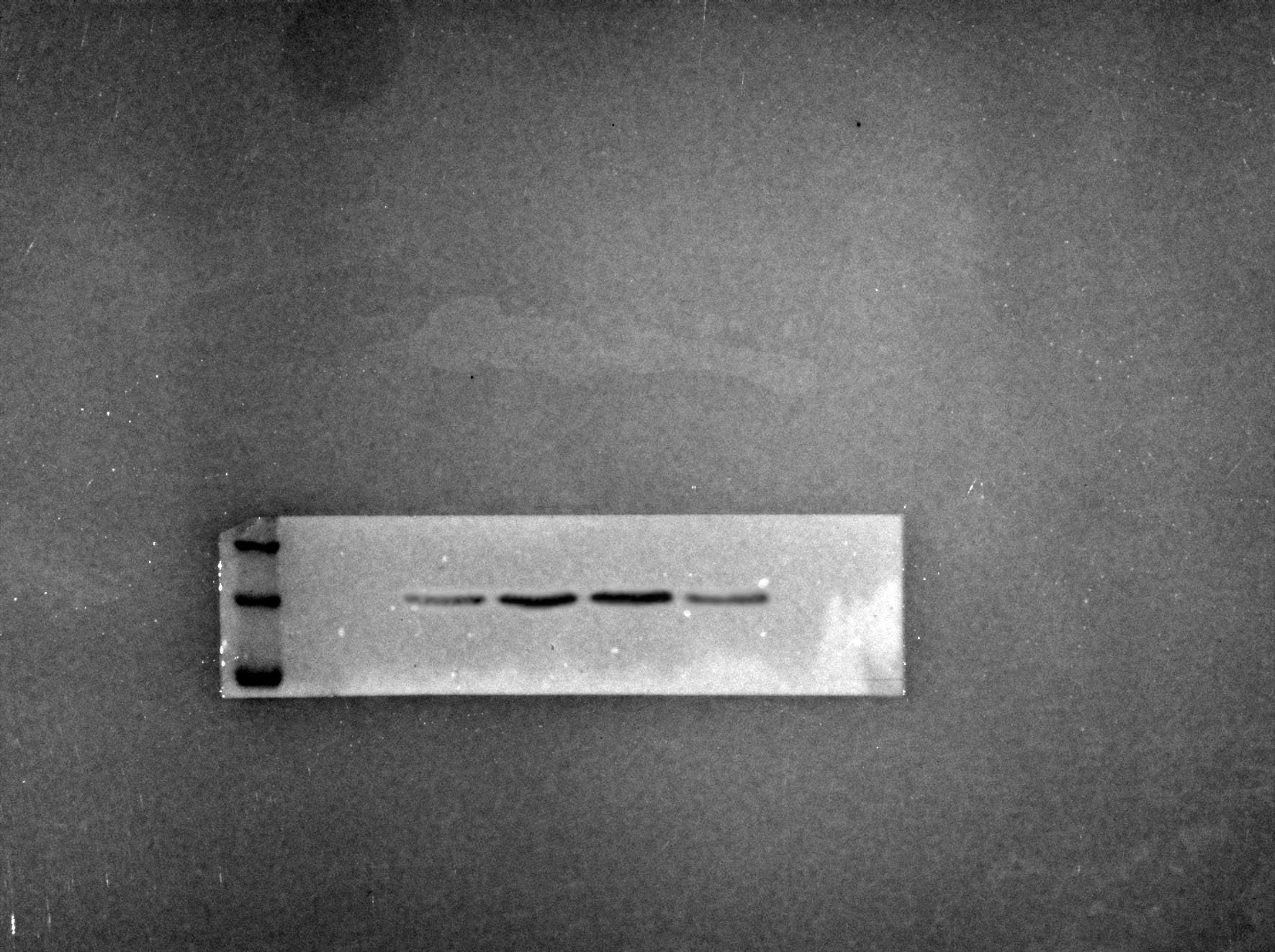

Supplement: Supplementary file 3 [file DataSheet1.zip › WB Supplementary/Supplementary Fig-3 B TNF-α.jpg]

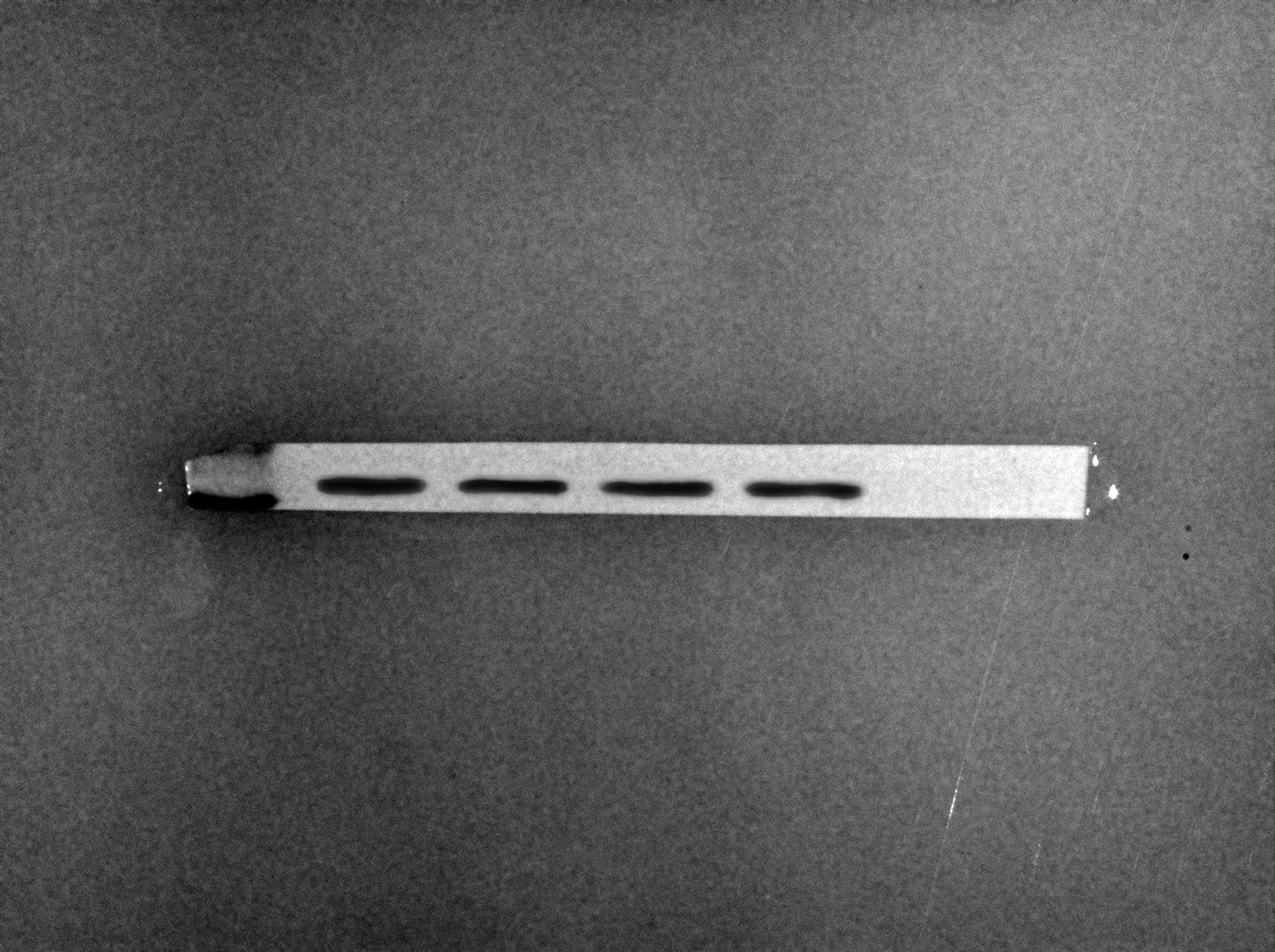

Supplement: Supplementary file 3 [file DataSheet1.zip › WB Supplementary/Supplementary Fig-3 B actin.jpg]

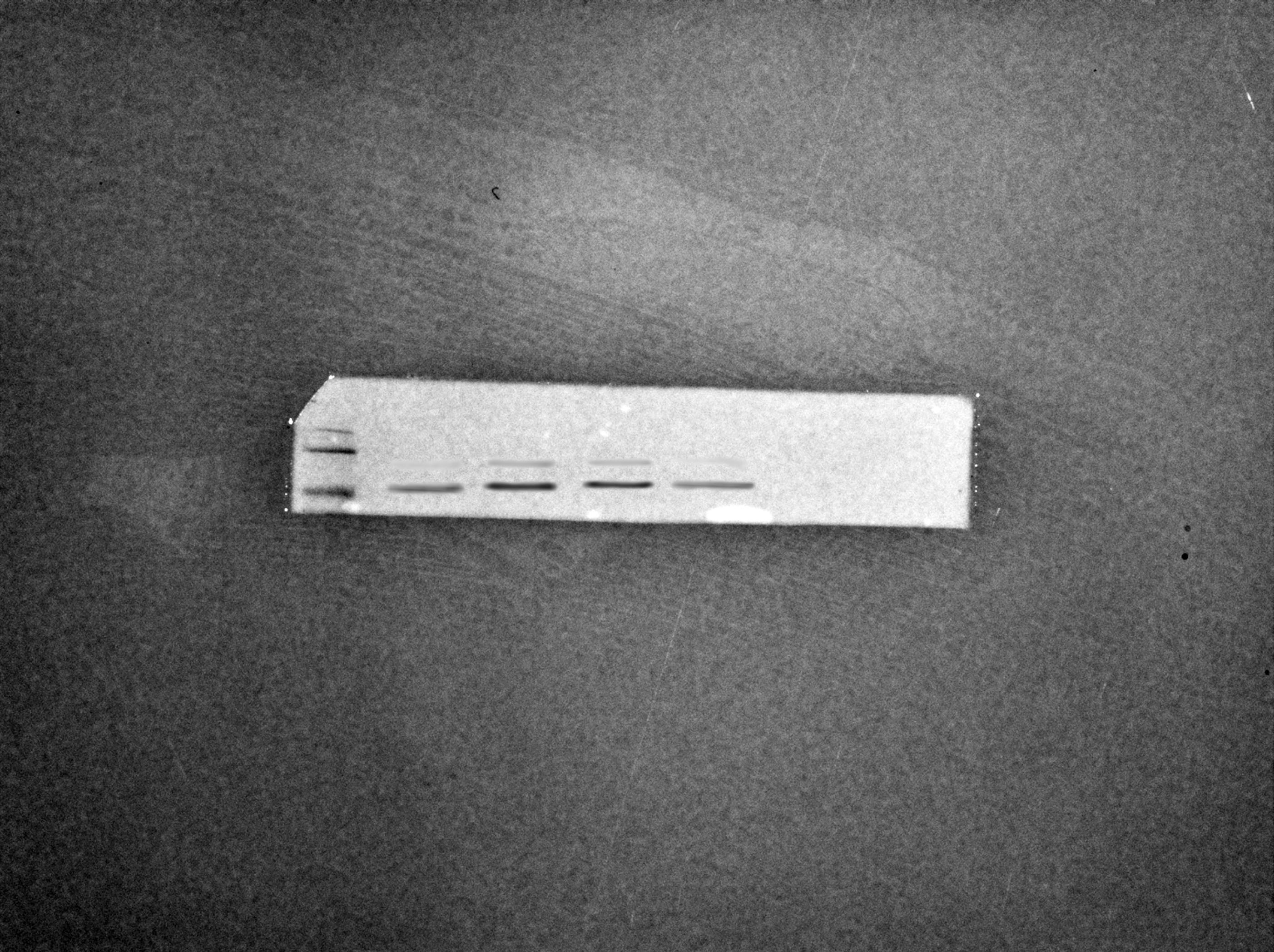

Supplement: Supplementary file 3 [file DataSheet1.zip › WB Supplementary/Supplementary Fig-3 B p-ERK.jpg]

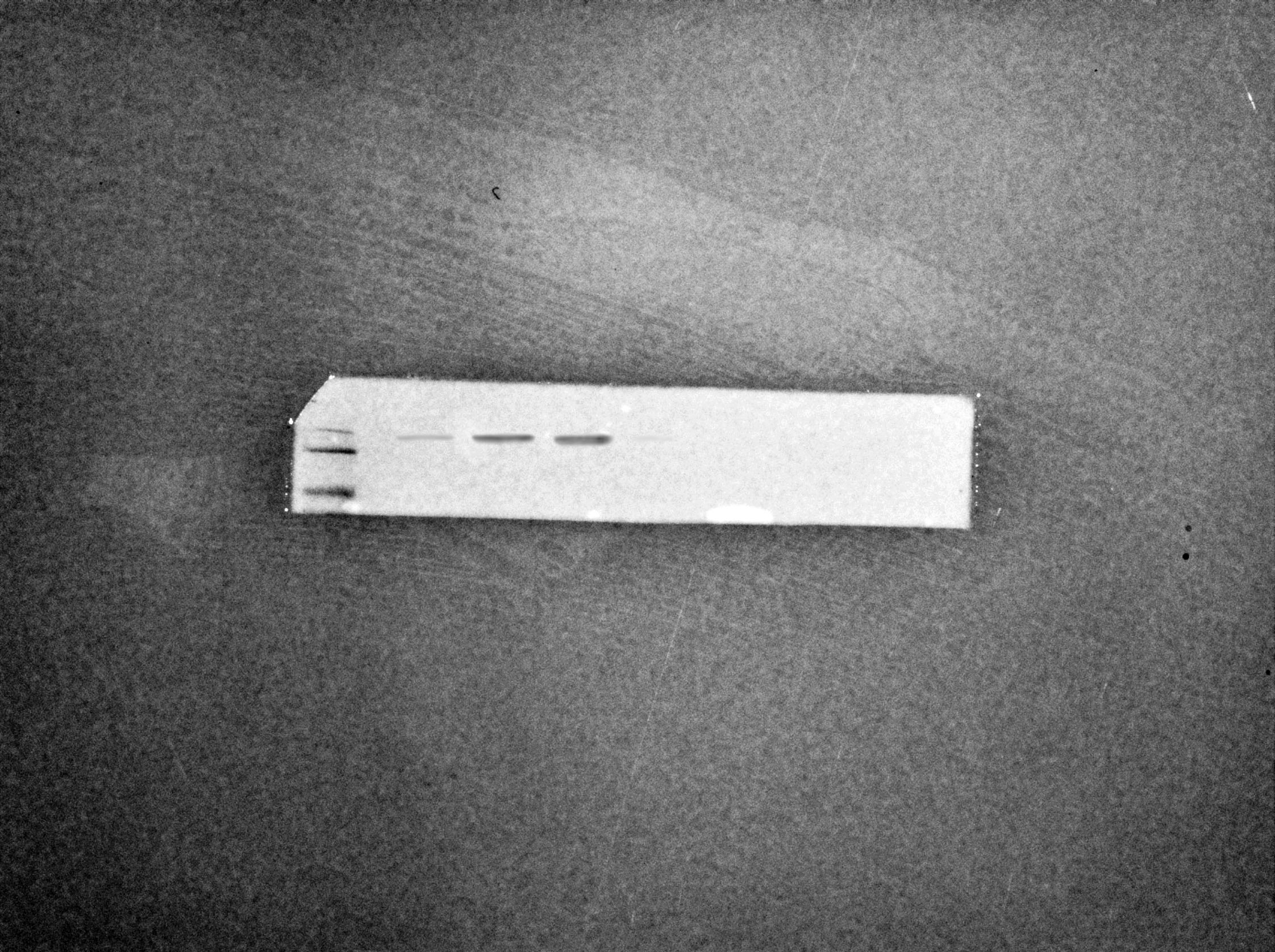

Supplement: Supplementary file 3 [file DataSheet1.zip › WB Supplementary/Supplementary Fig-3 B p-NF-κB.jpg]

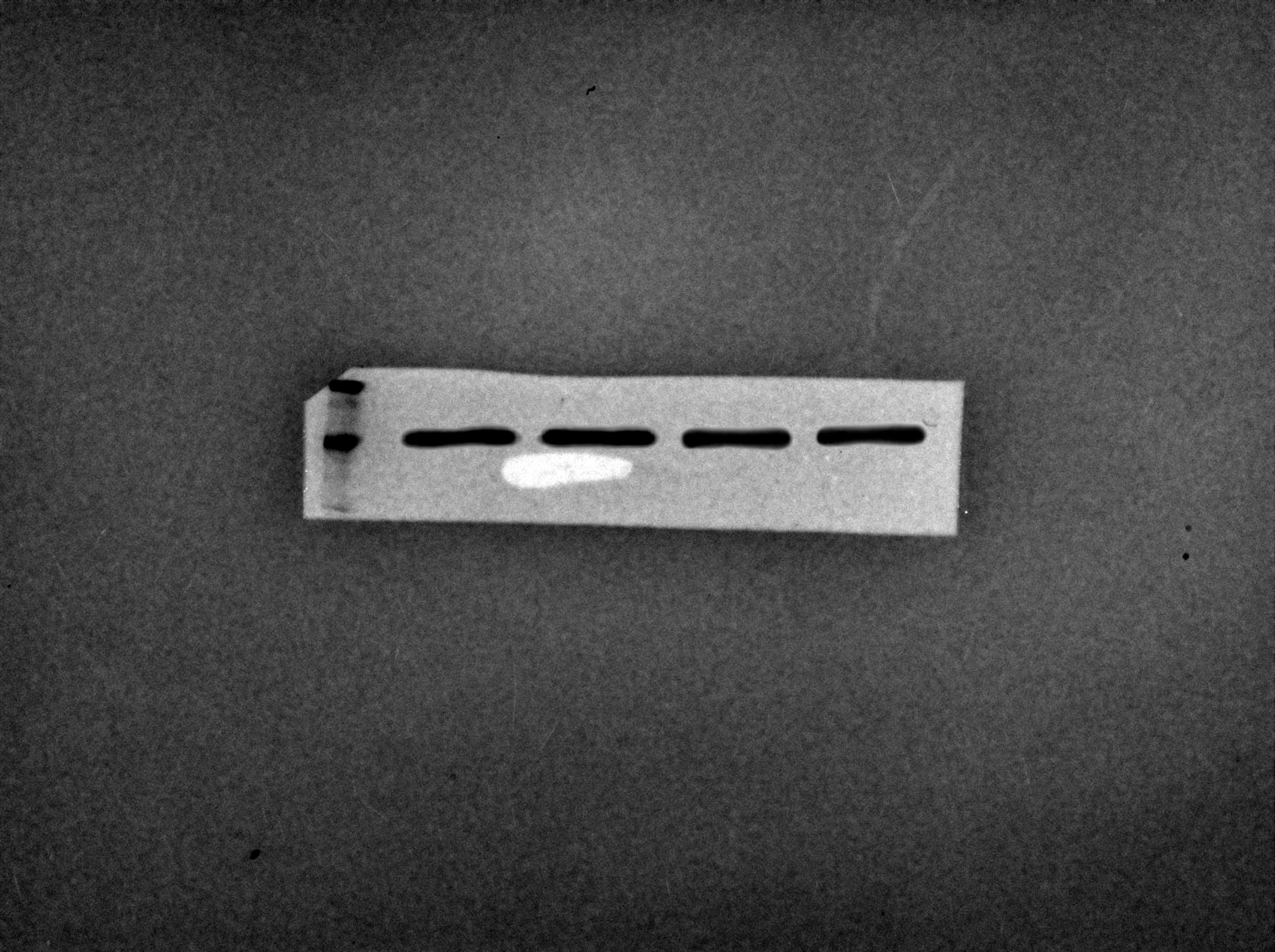

Supplement: Supplementary file 3 [file DataSheet1.zip › WB Supplementary/Supplementary Fig-5 A actin.jpg]

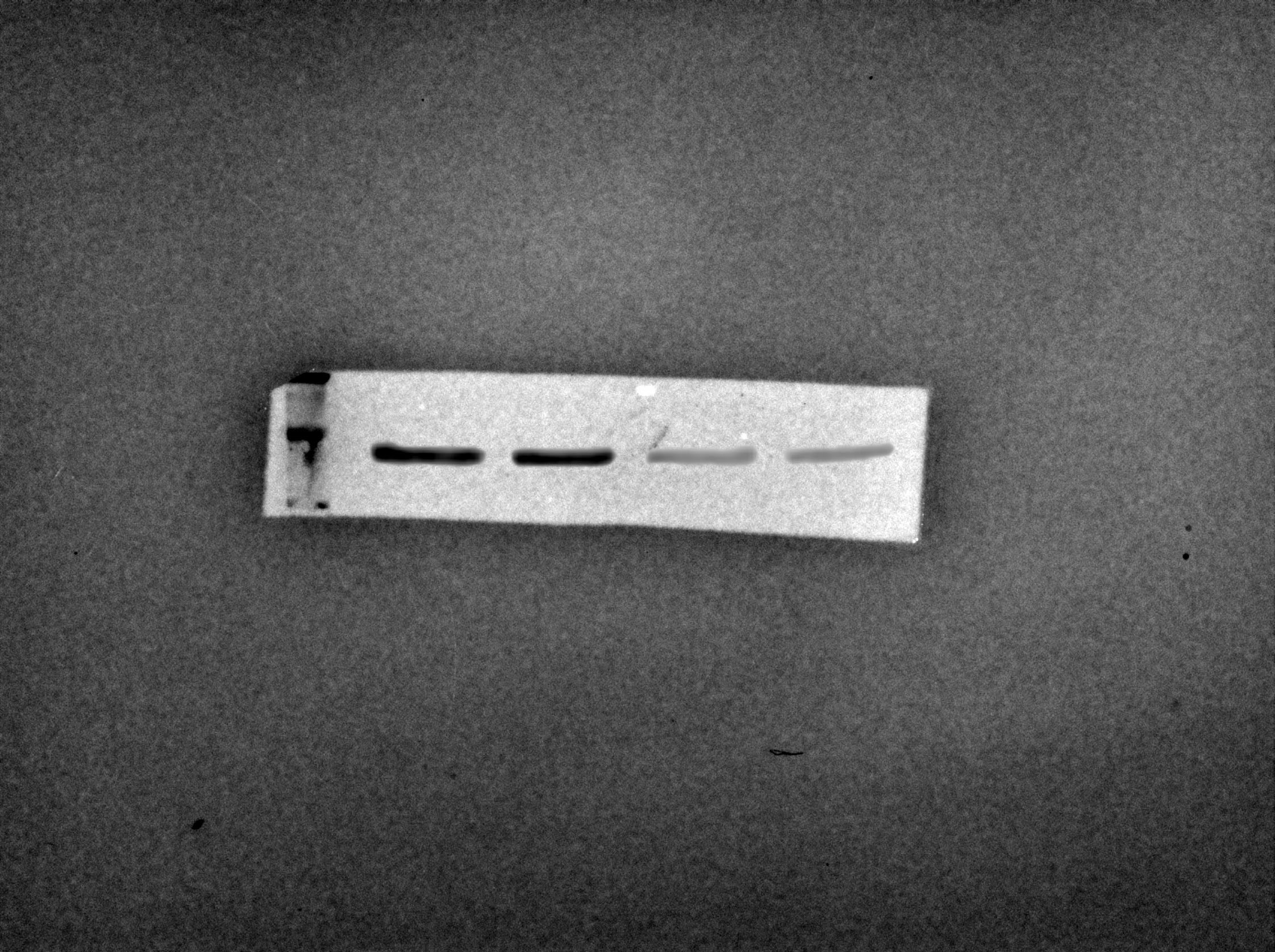

Supplement: Supplementary file 3 [file DataSheet1.zip › WB Supplementary/Supplementary Fig-5 A α-SMA.jpg]

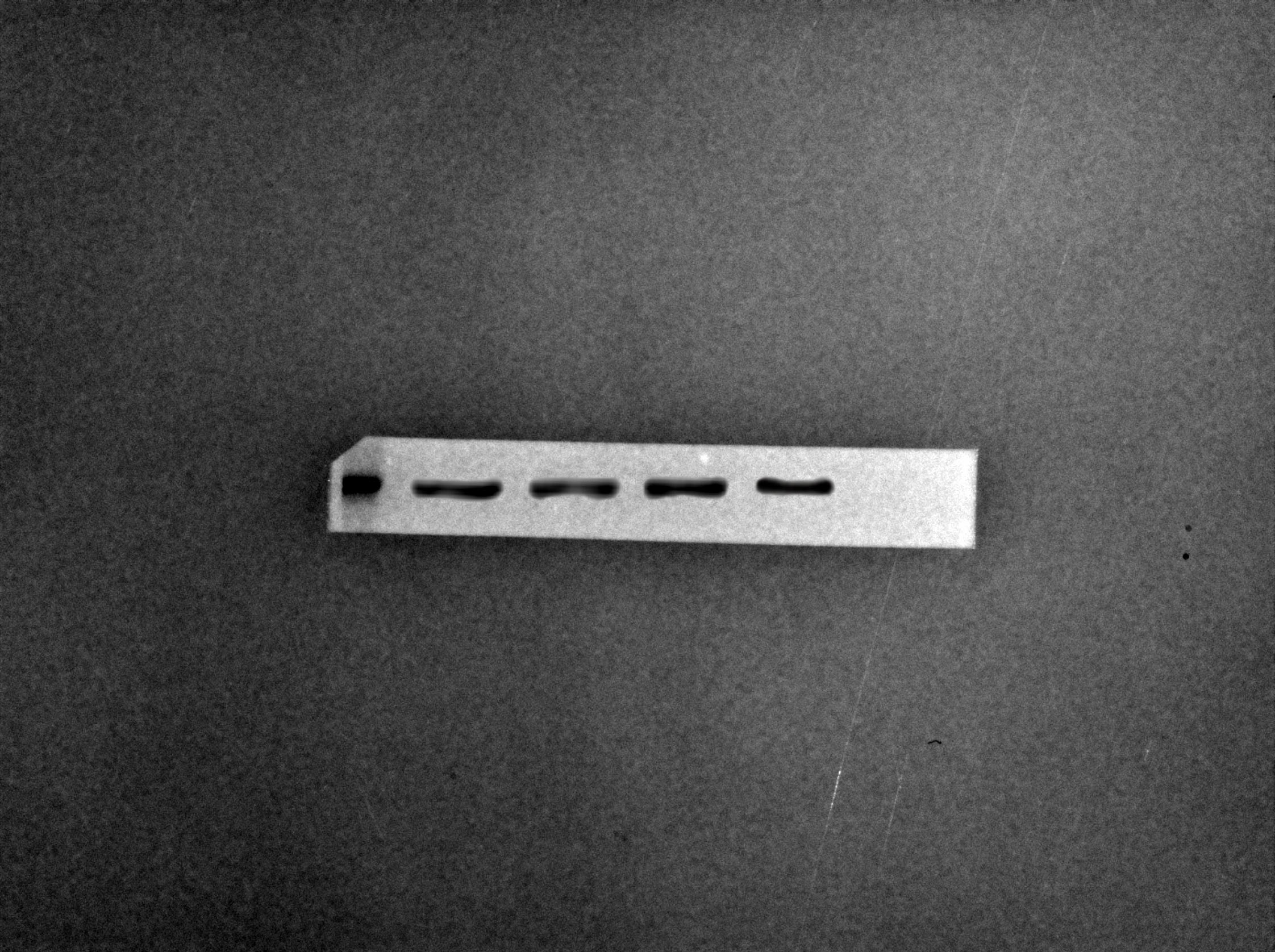

Supplement: Supplementary file 3 [file DataSheet1.zip › WB Supplementary/Supplementary Fig-5 B actin.jpg]

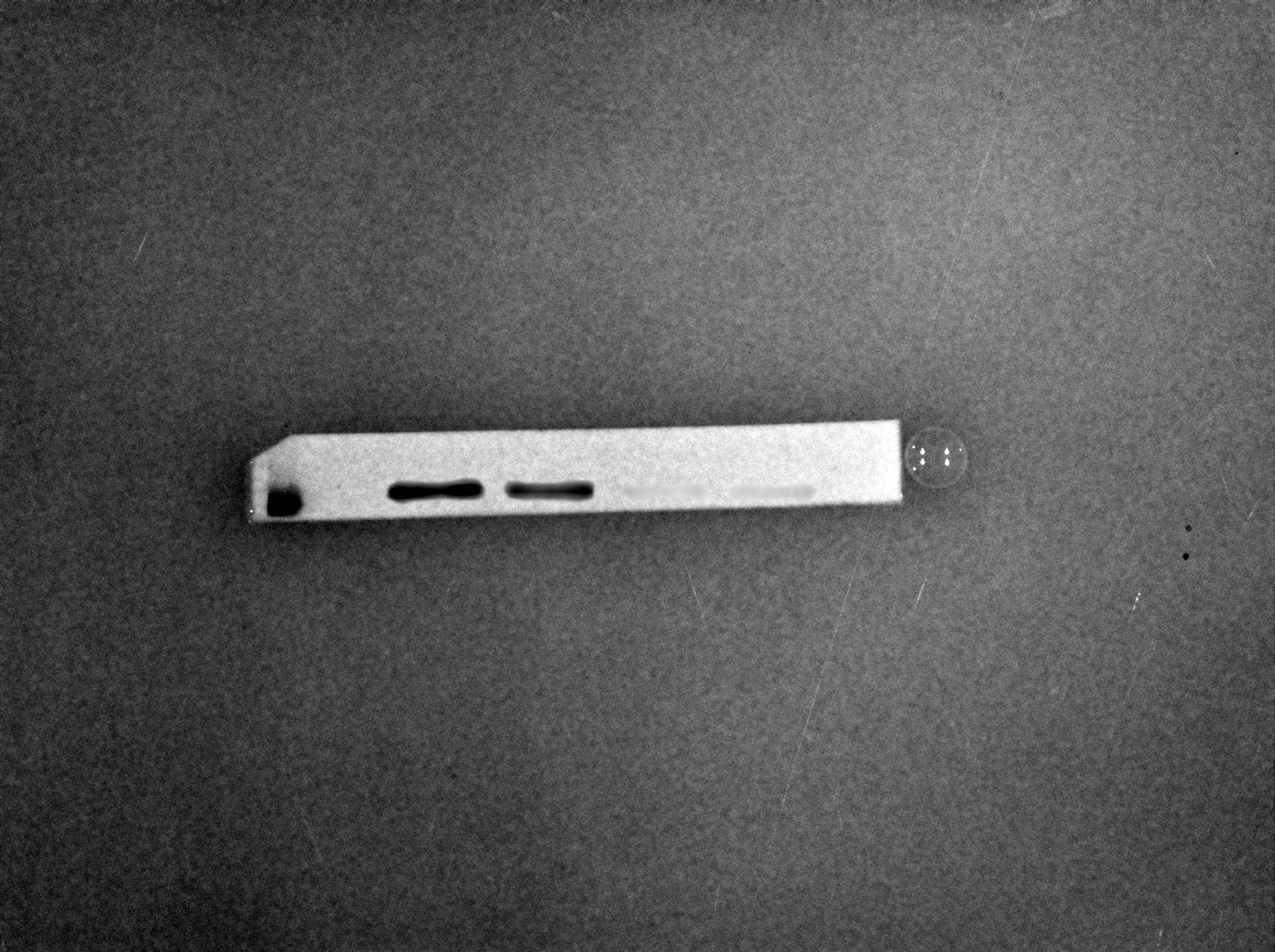

Supplement: Supplementary file 3 [file DataSheet1.zip › WB Supplementary/Supplementary Fig-5 B α-SMA.jpg]

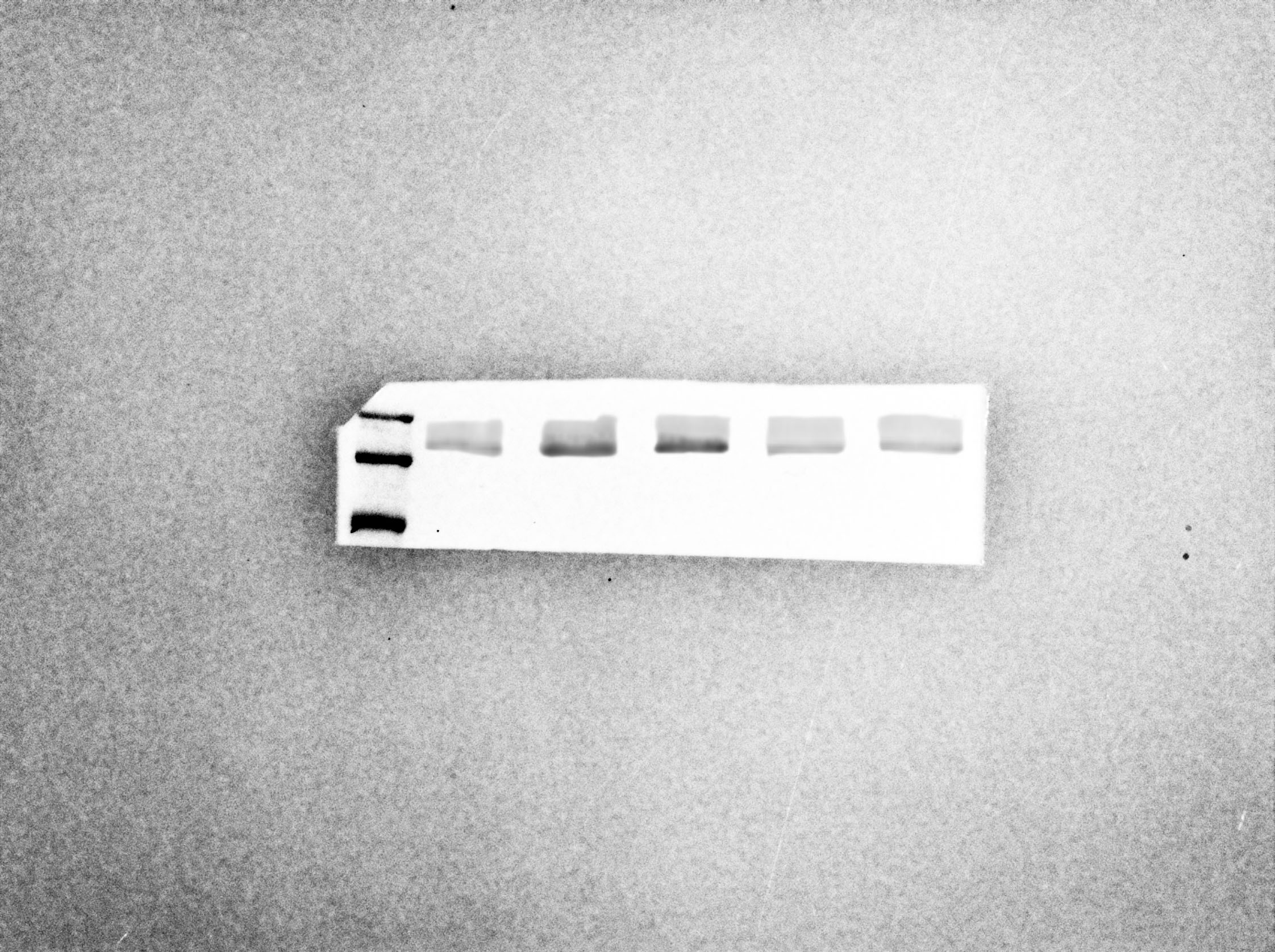

Supplement: Supplementary file 3 [file DataSheet1.zip › WB Supplementary/Supplementary Fig-5 F Collagen I.jpg]

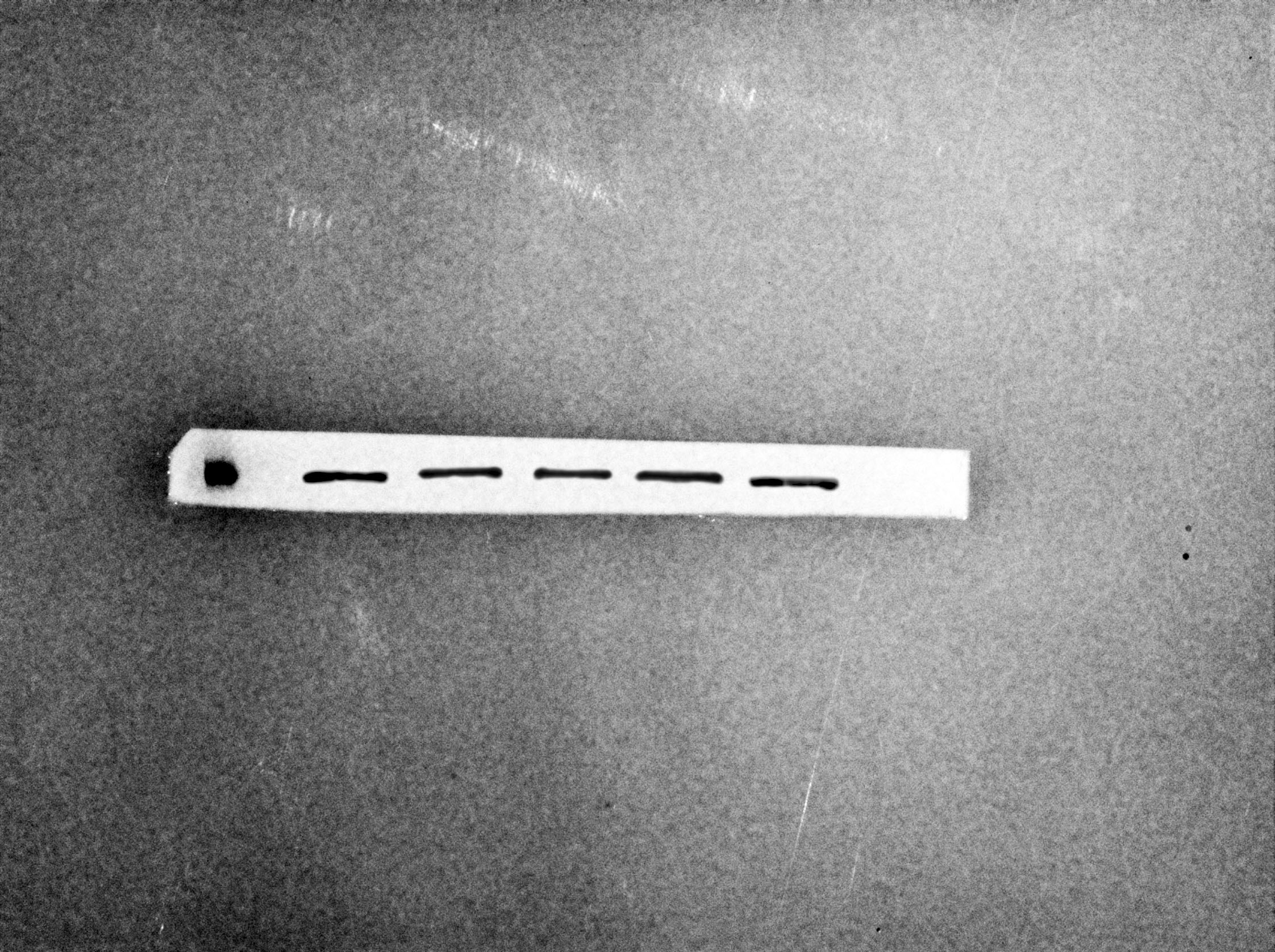

Supplement: Supplementary file 3 [file DataSheet1.zip › WB Supplementary/Supplementary Fig-5 F actin.jpg]

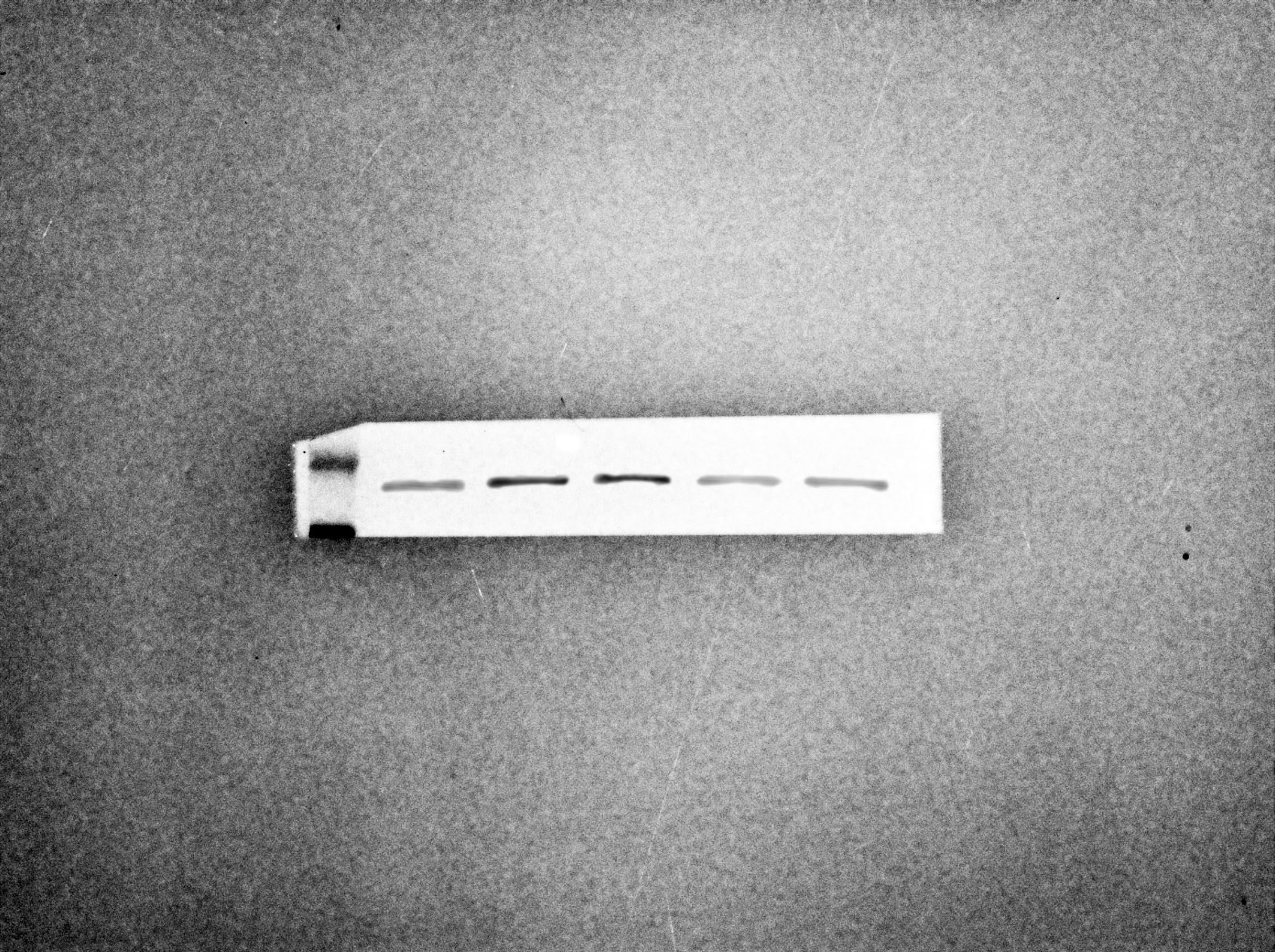

Supplement: Supplementary file 3 [file DataSheet1.zip › WB Supplementary/Supplementary Fig-5 F α-SMA.jpg]

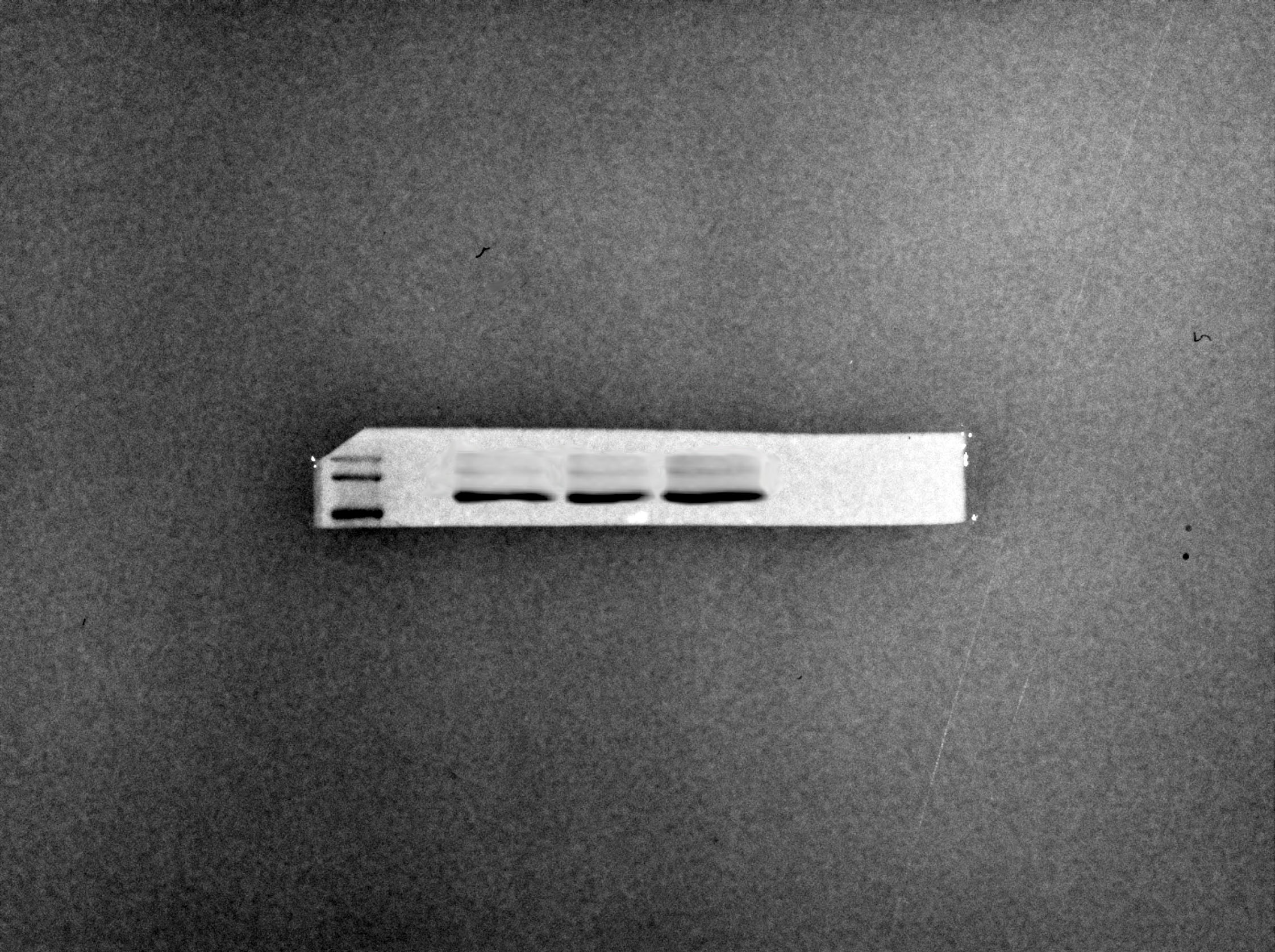

Supplement: Supplementary file 3 [file DataSheet1.zip › WB Supplementary/Supplementary Fig-6 A ERK.jpg]

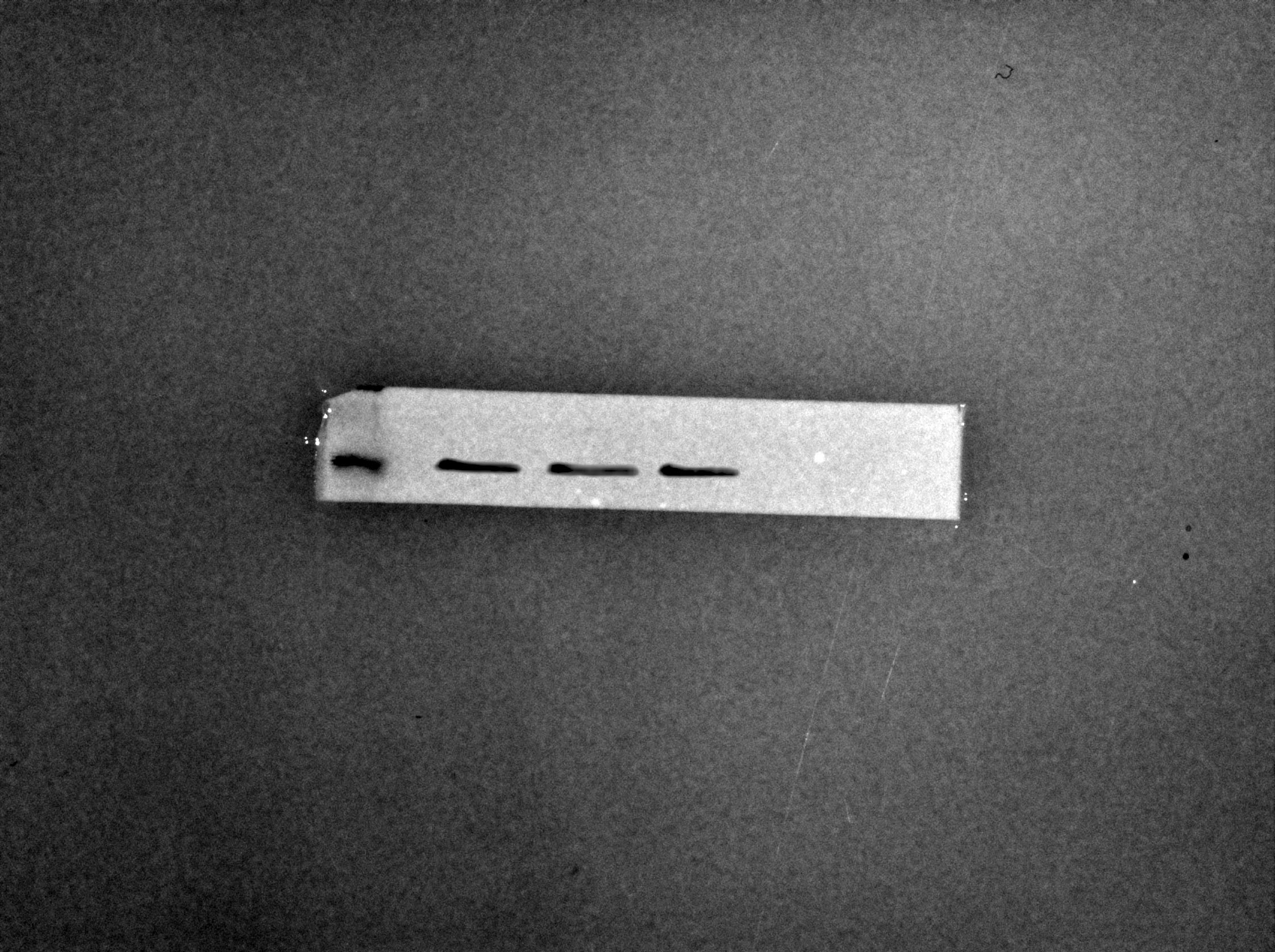

Supplement: Supplementary file 3 [file DataSheet1.zip › WB Supplementary/Supplementary Fig-6 A NF-κB.jpg]

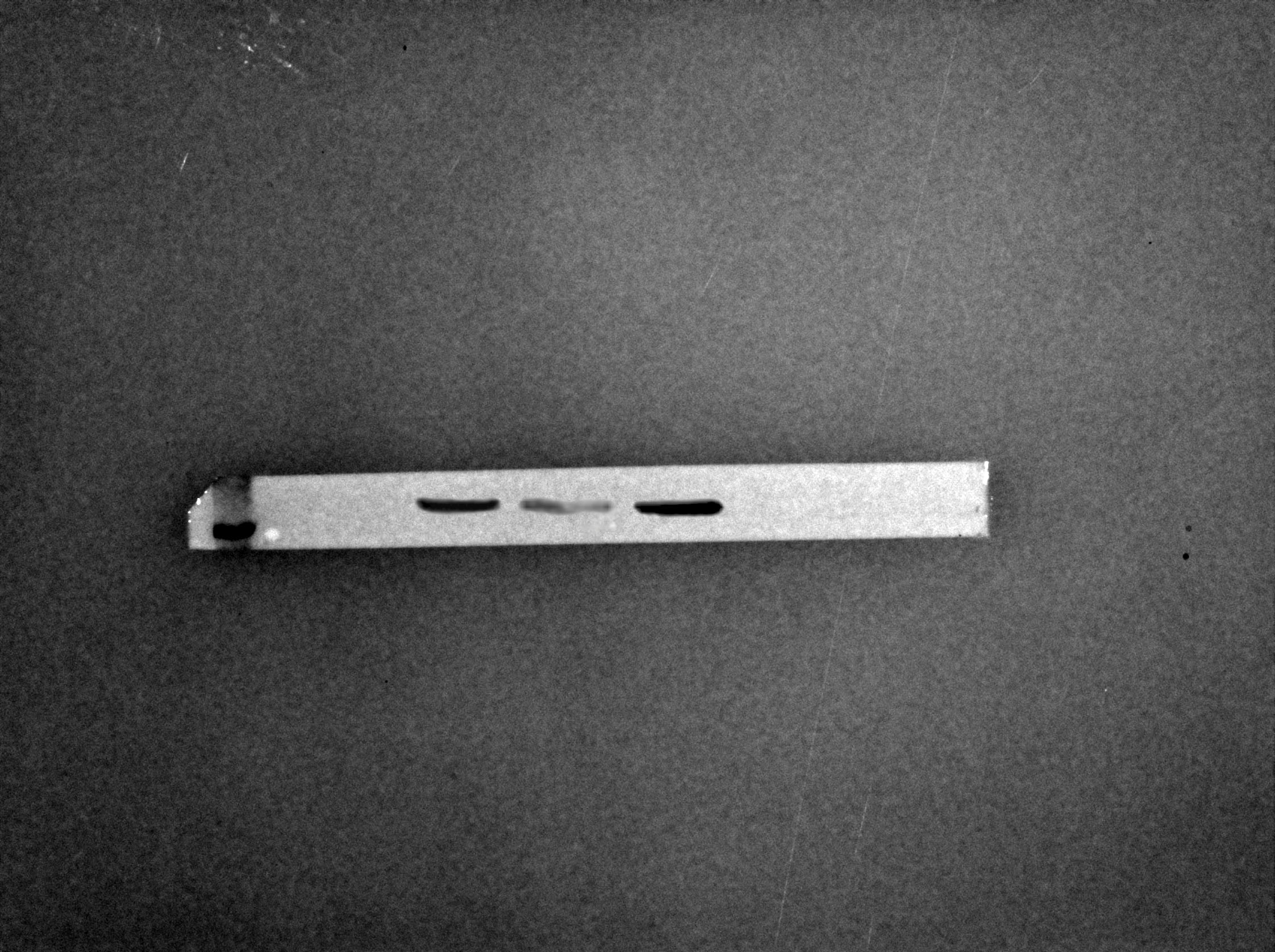

Supplement: Supplementary file 3 [file DataSheet1.zip › WB Supplementary/Supplementary Fig-6 A SPRY1.jpg]

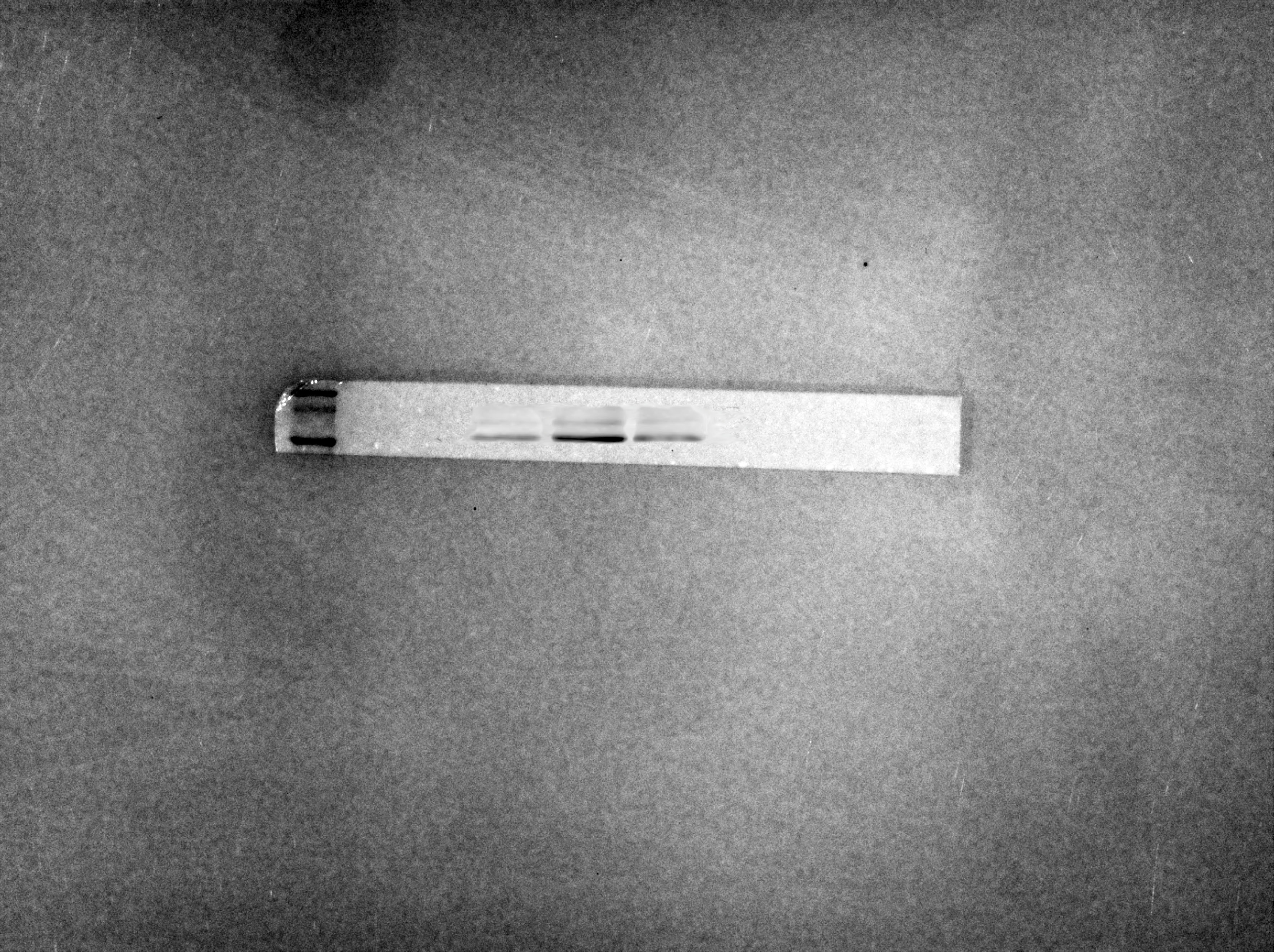

Supplement: Supplementary file 3 [file DataSheet1.zip › WB Supplementary/Supplementary Fig-6 A p-ERK.jpg]

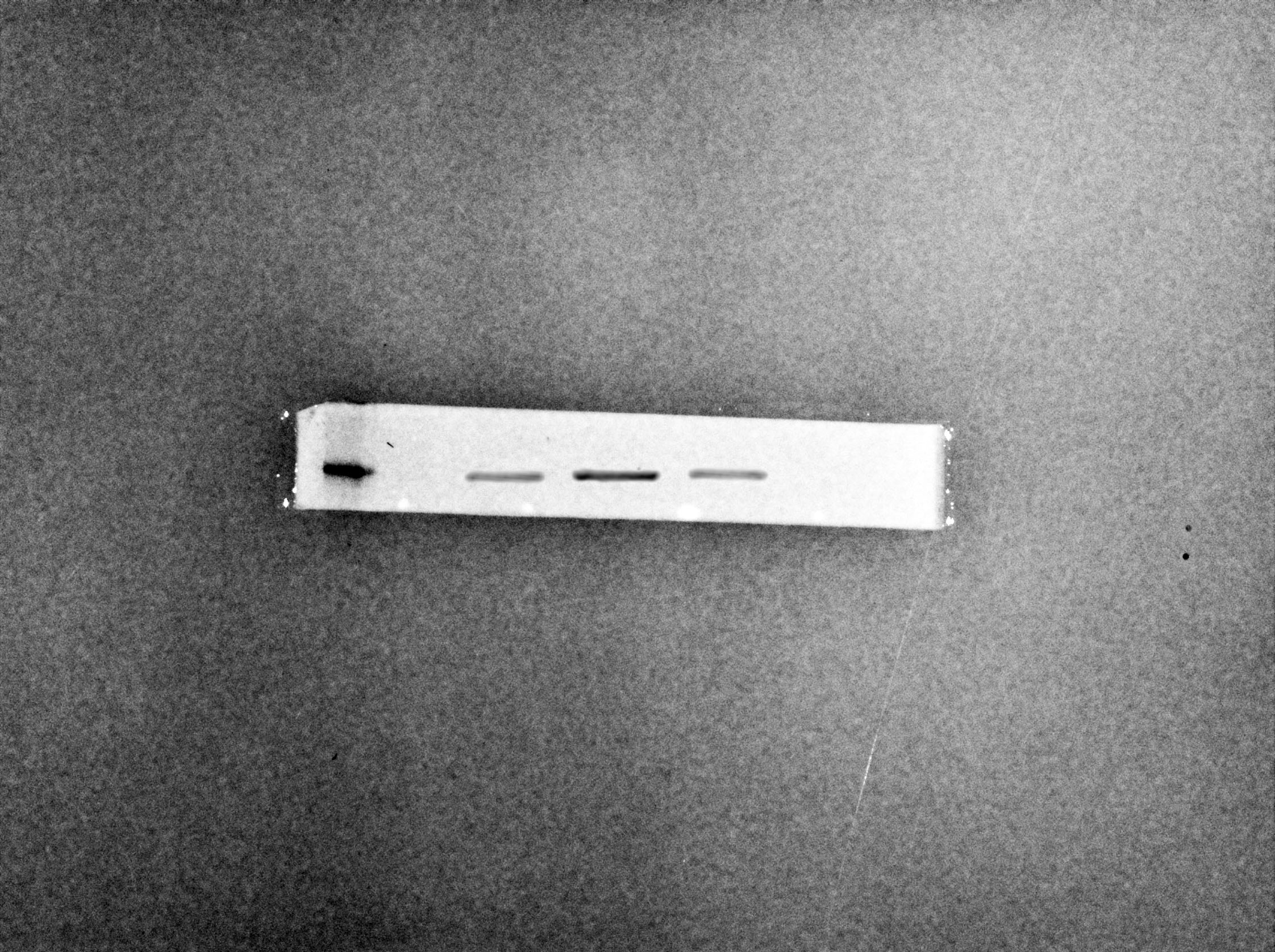

Supplement: Supplementary file 3 [file DataSheet1.zip › WB Supplementary/Supplementary Fig-6 A p-NF-κB.jpg]

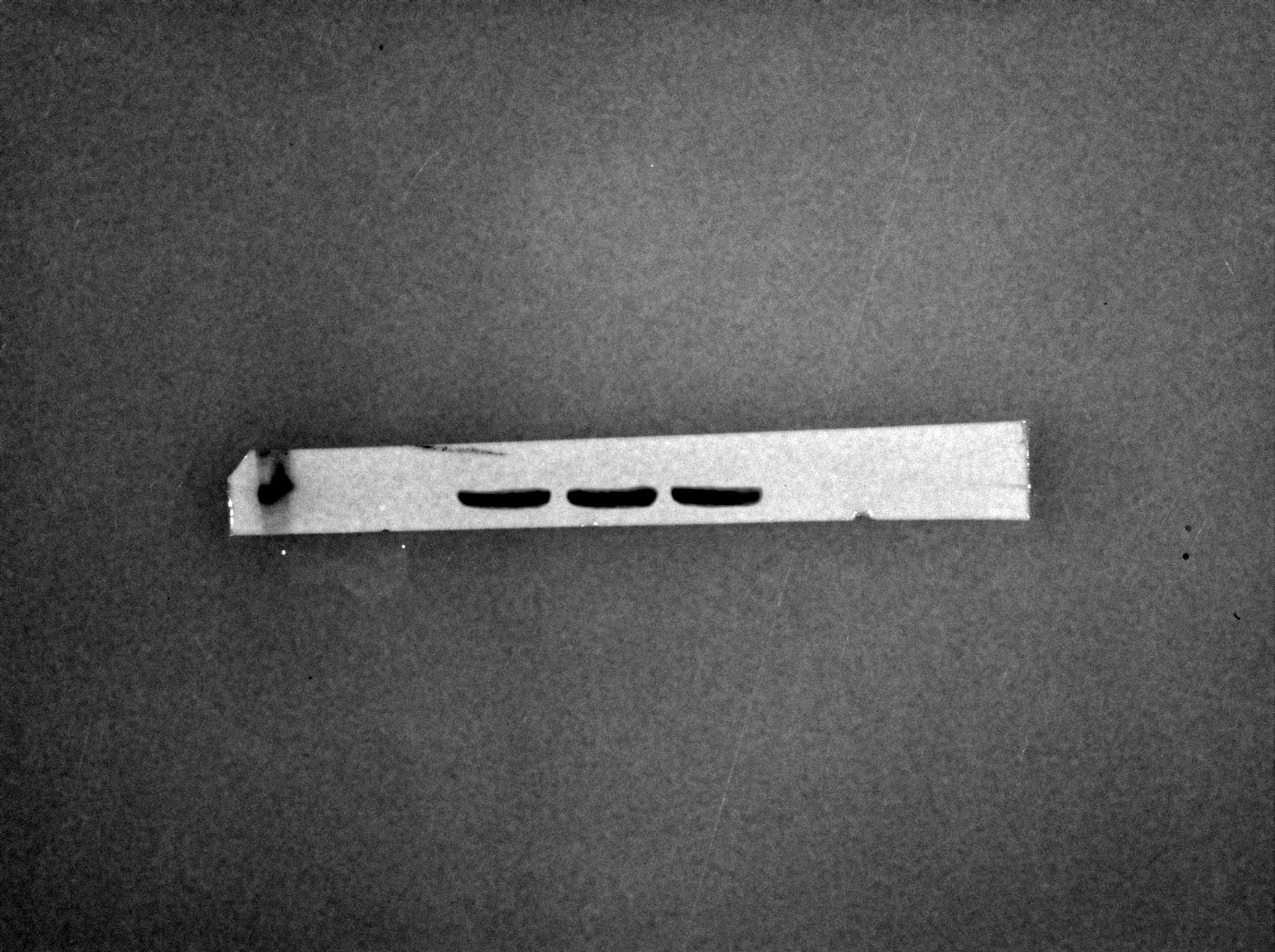

Supplement: Supplementary file 3 [file DataSheet1.zip › WB Supplementary/Supplementary Fig-6 A α-SMA.jpg]

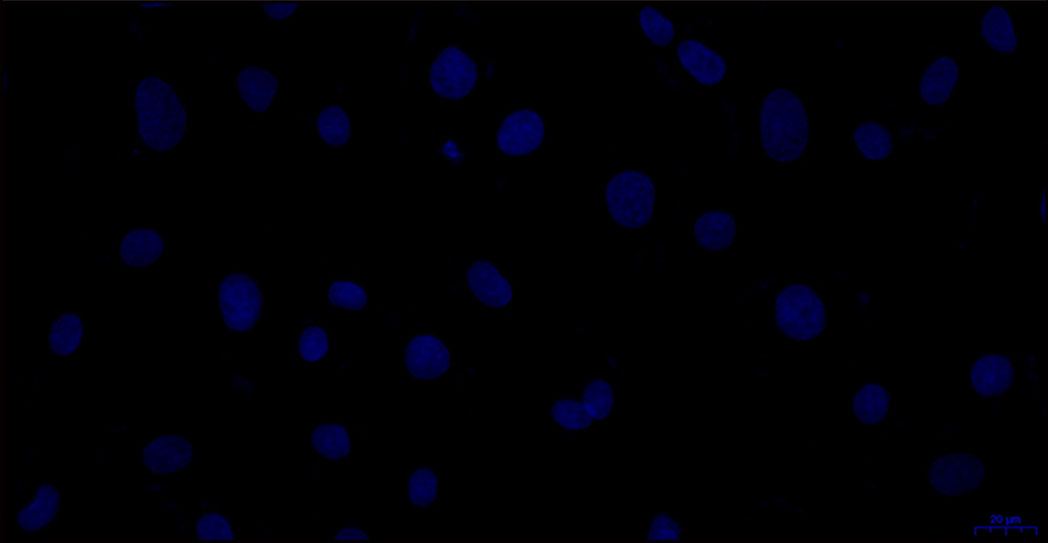

Supplement: Supplementary file 4 [file DataSheet6.zip › Supplementary Figure 6/miR-21-5p mimics +DIAP.jpg]

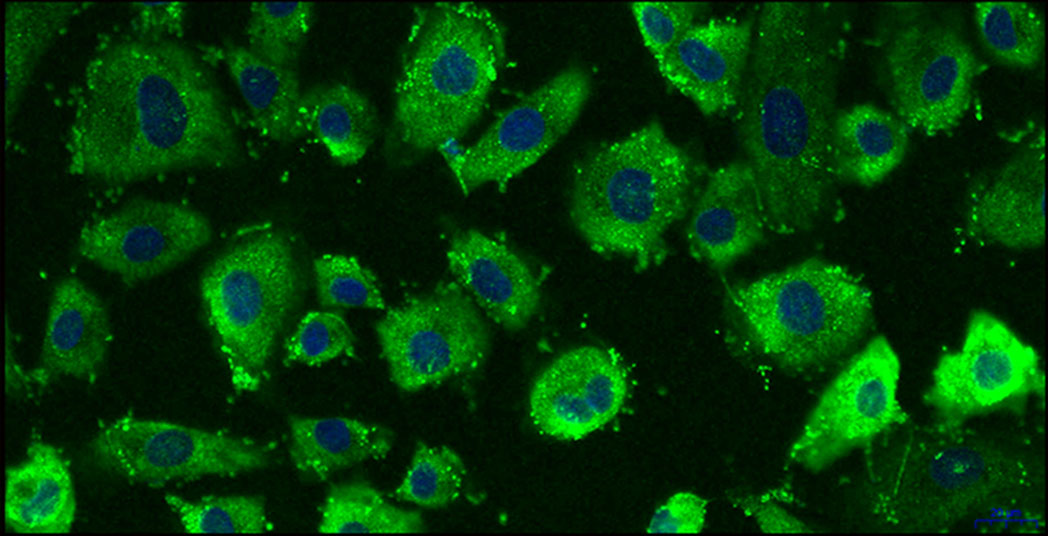

Supplement: Supplementary file 4 [file DataSheet6.zip › Supplementary Figure 6/miR-21-5p mimics +Merge.jpg]

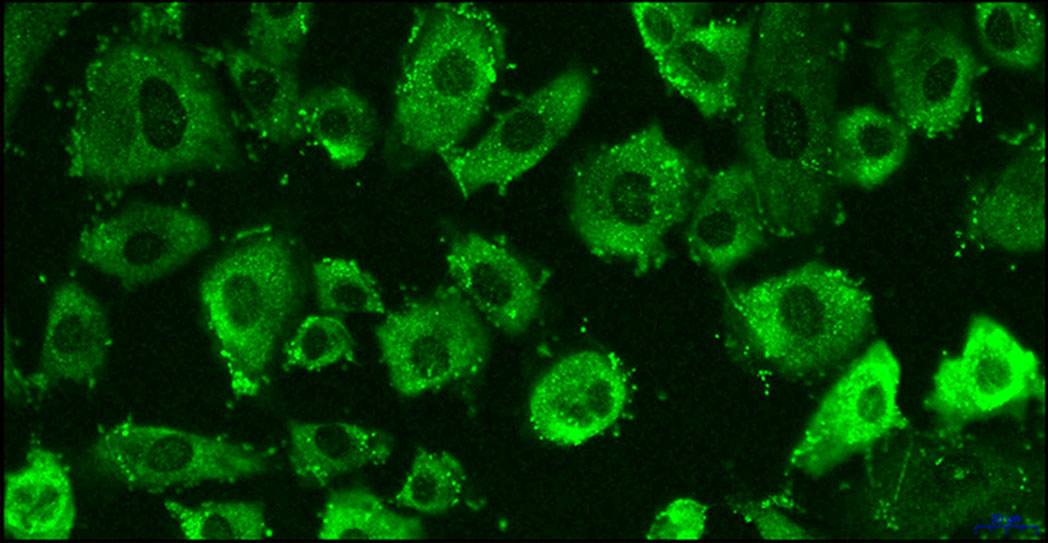

Supplement: Supplementary file 4 [file DataSheet6.zip › Supplementary Figure 6/miR-21-5p mimics +α-SMA.jpg]

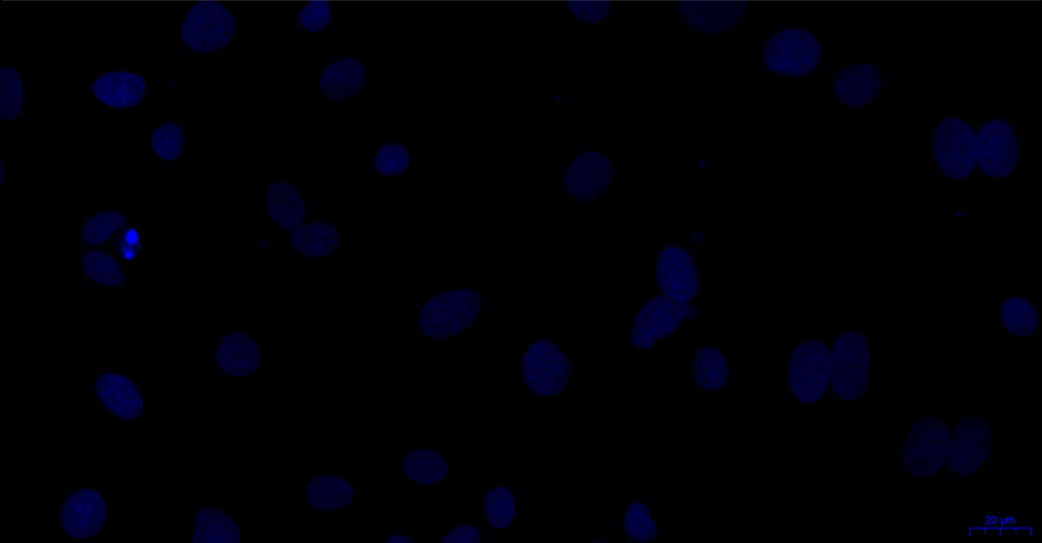

Supplement: Supplementary file 4 [file DataSheet6.zip › Supplementary Figure 6/miR-21-5p mimics+EPO +DIAP.jpg]

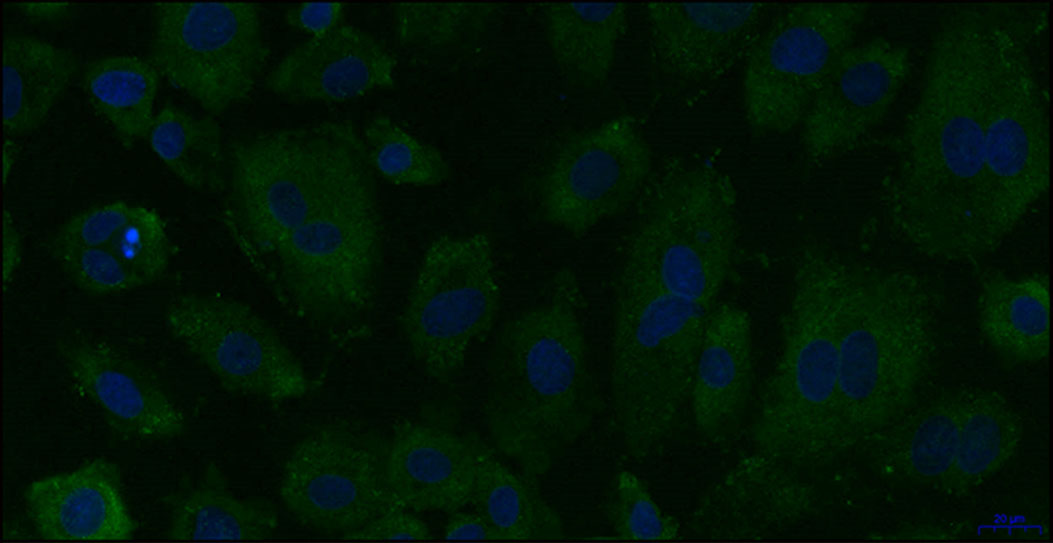

Supplement: Supplementary file 4 [file DataSheet6.zip › Supplementary Figure 6/miR-21-5p mimics+EPO +Merge.jpg]

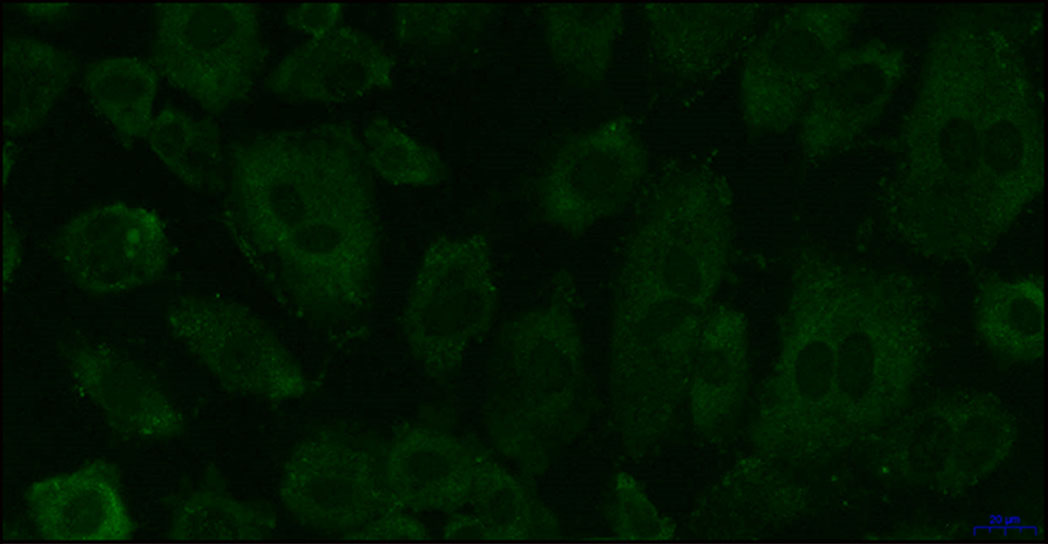

Supplement: Supplementary file 4 [file DataSheet6.zip › Supplementary Figure 6/miR-21-5p mimics+EPO +α-SMA.jpg]

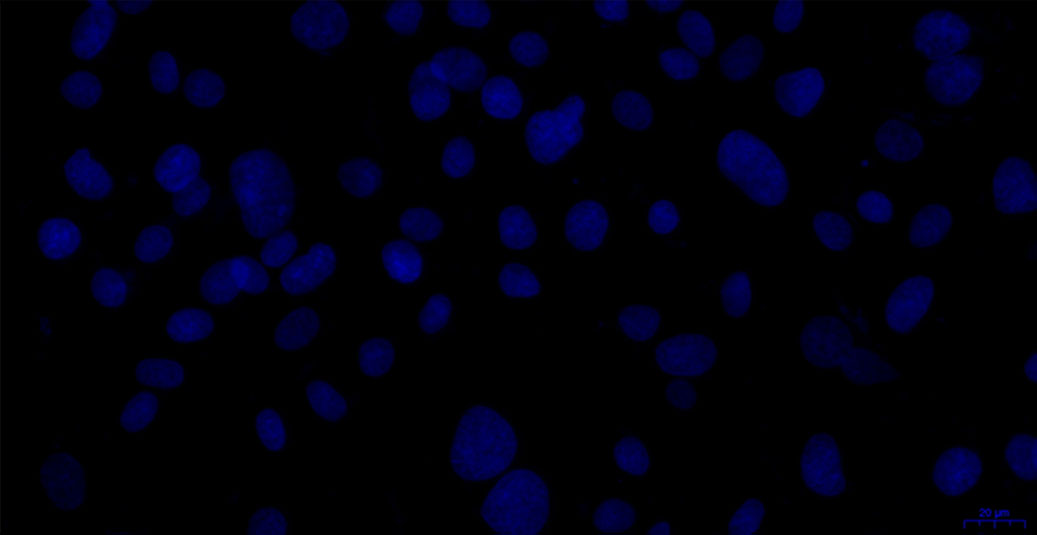

Supplement: Supplementary file 4 [file DataSheet6.zip › Supplementary Figure 6/miR-NC+DIAP.jpg]

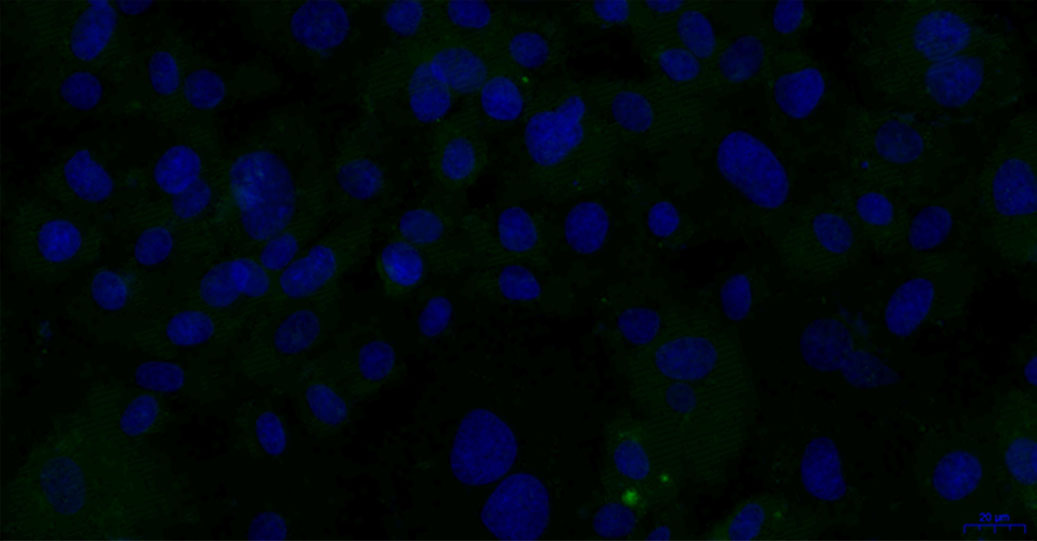

Supplement: Supplementary file 4 [file DataSheet6.zip › Supplementary Figure 6/miR-NC+Merge.jpg]

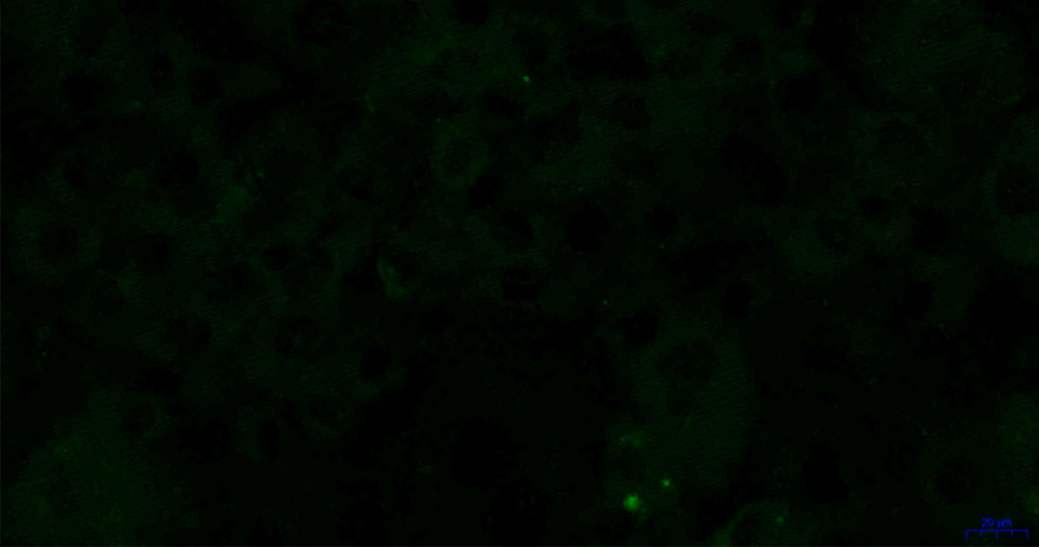

Supplement: Supplementary file 4 [file DataSheet6.zip › Supplementary Figure 6/miR-NC+α-SMA.jpg]

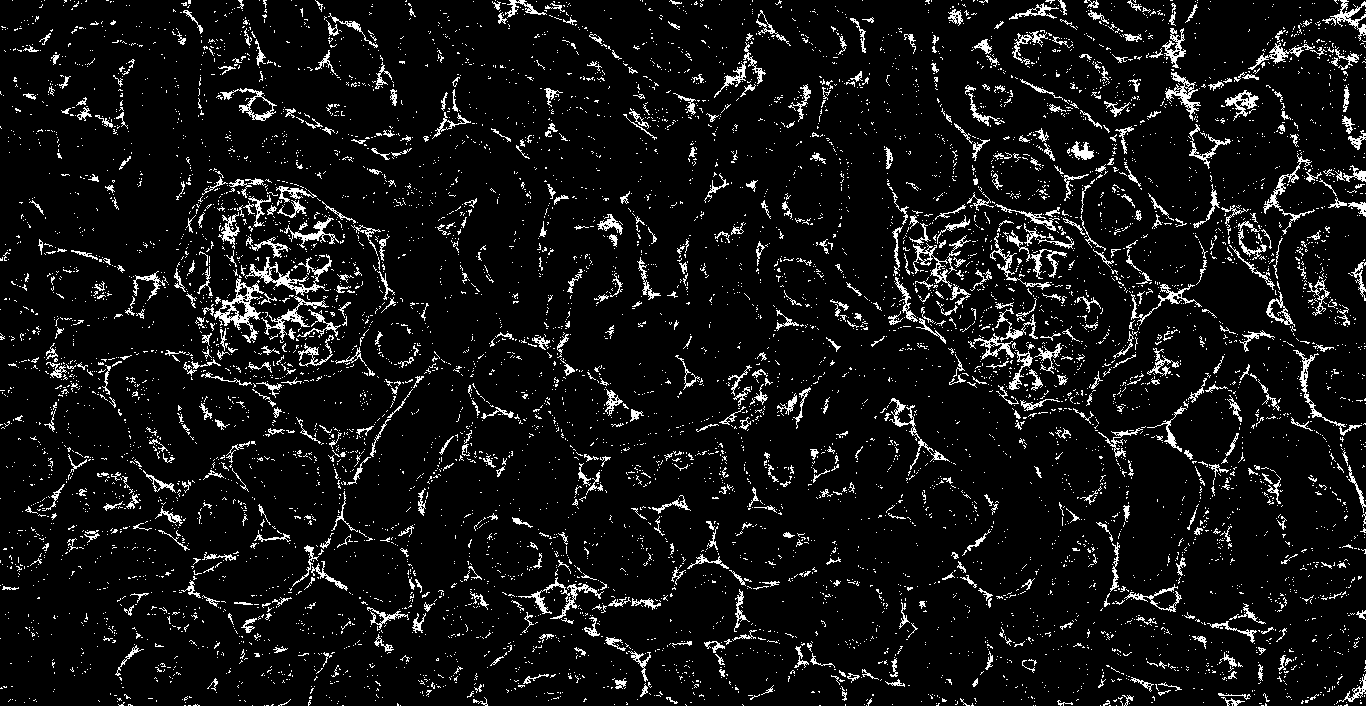

Supplement: Supplementary file 5 [file DataSheet2.zip › Supplementary Figure 1 A/Blue-stained area of MASSON SHAM_20.0x.tif]

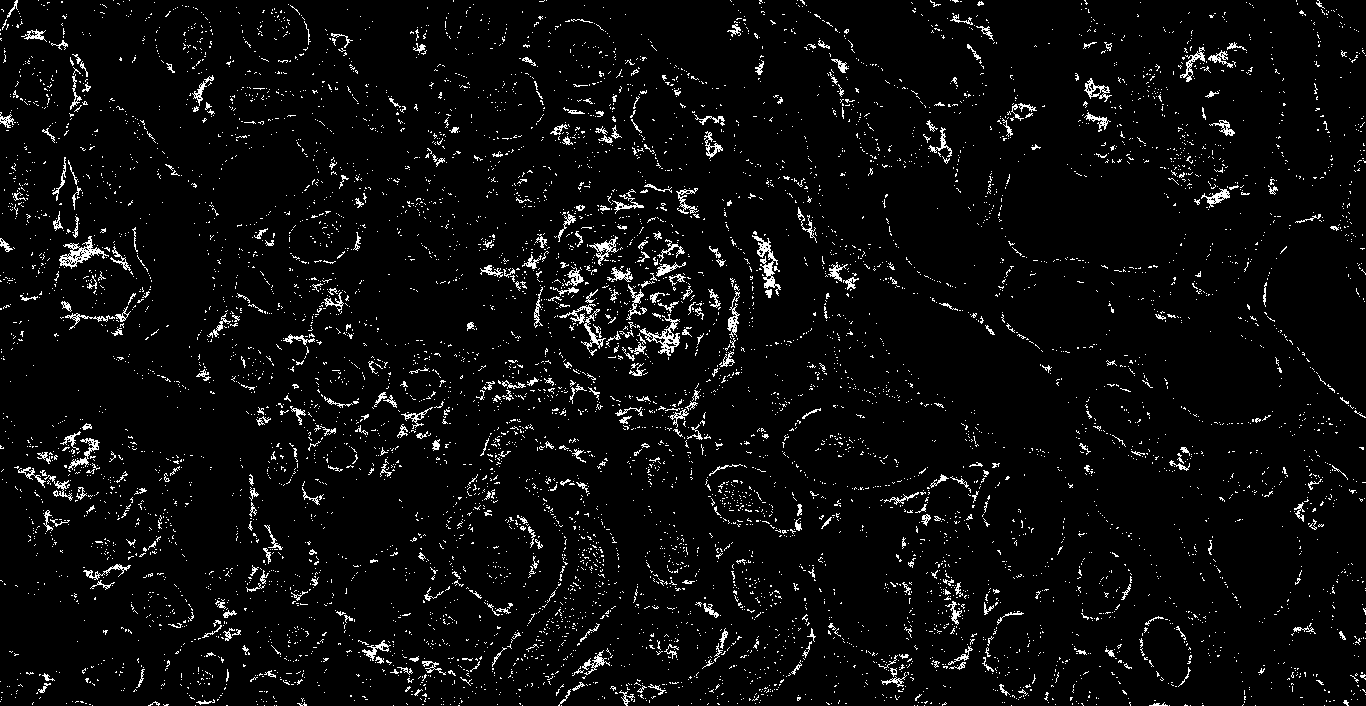

Supplement: Supplementary file 5 [file DataSheet2.zip › Supplementary Figure 1 A/Blue-stained area of MASSON UUO+EPO_20.0x.tif]

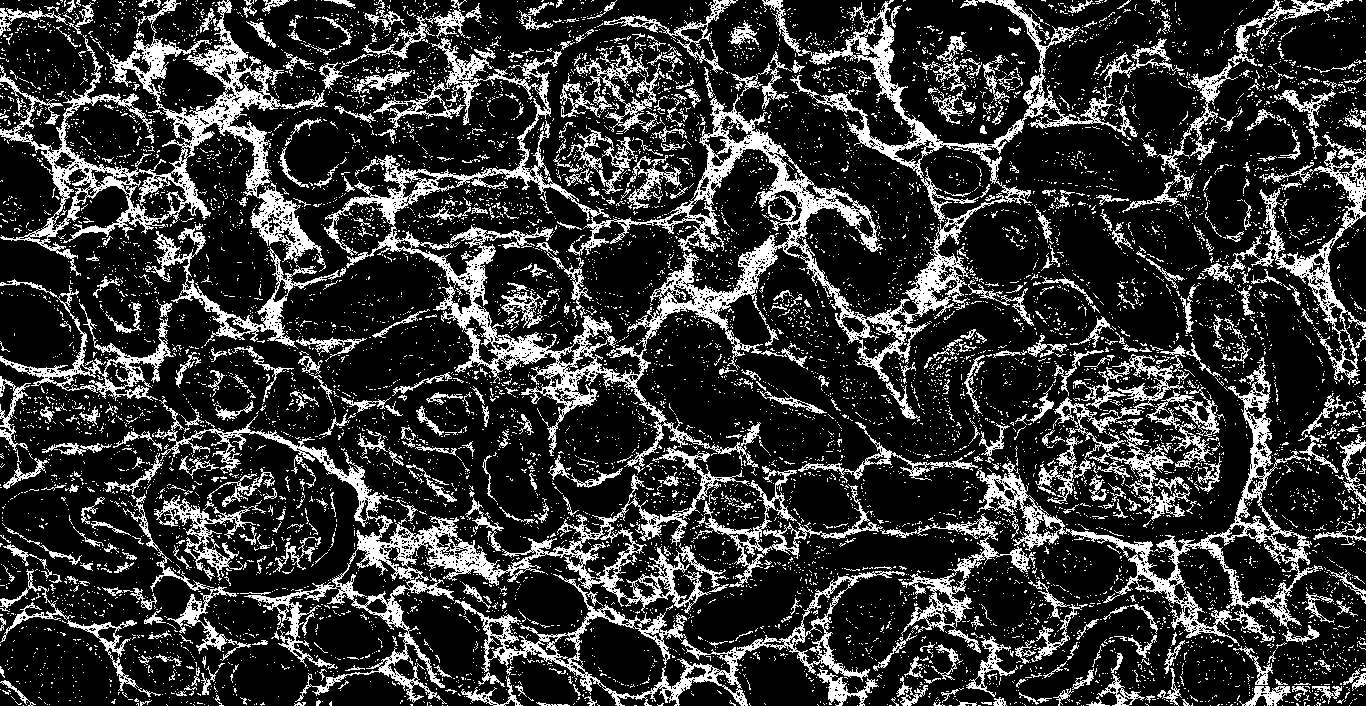

Supplement: Supplementary file 5 [file DataSheet2.zip › Supplementary Figure 1 A/Blue-stained area of MASSON UUO+saline_20.0x.tif]

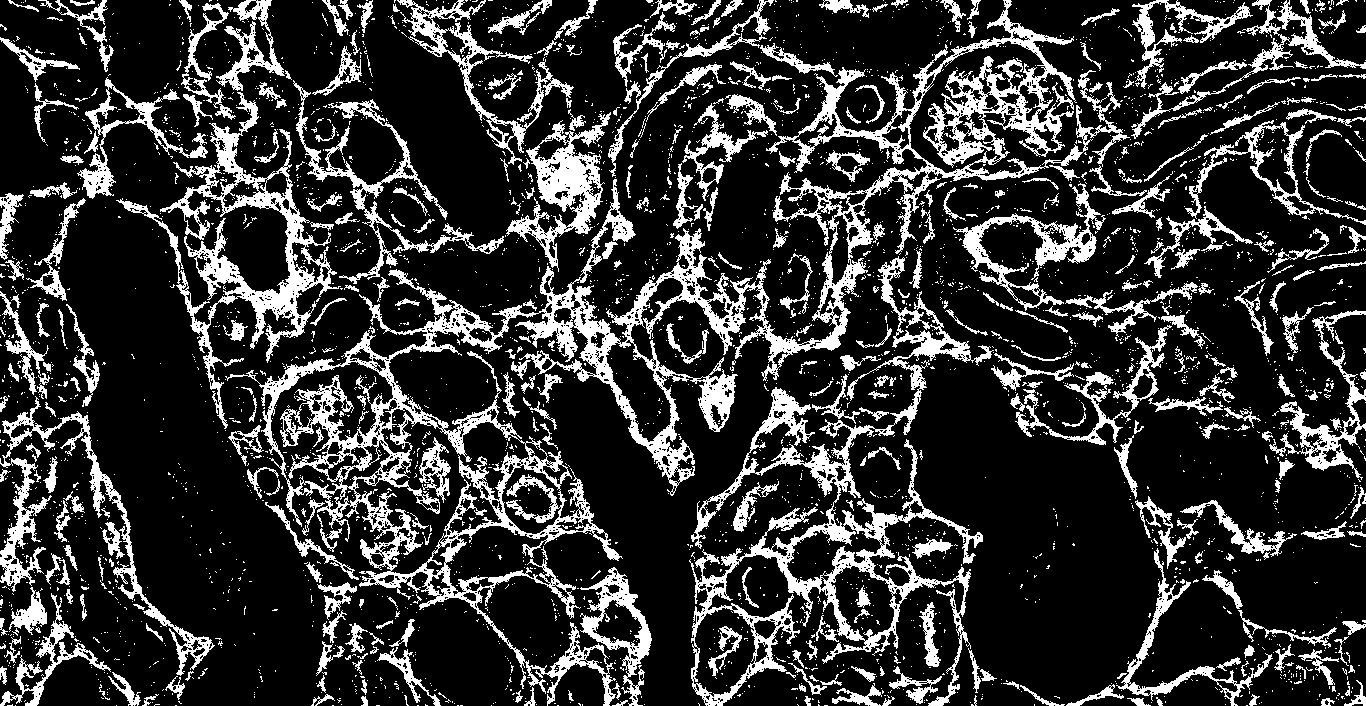

Supplement: Supplementary file 5 [file DataSheet2.zip › Supplementary Figure 1 A/Blue-stained area of MASSON UUO_20.0x.tif]

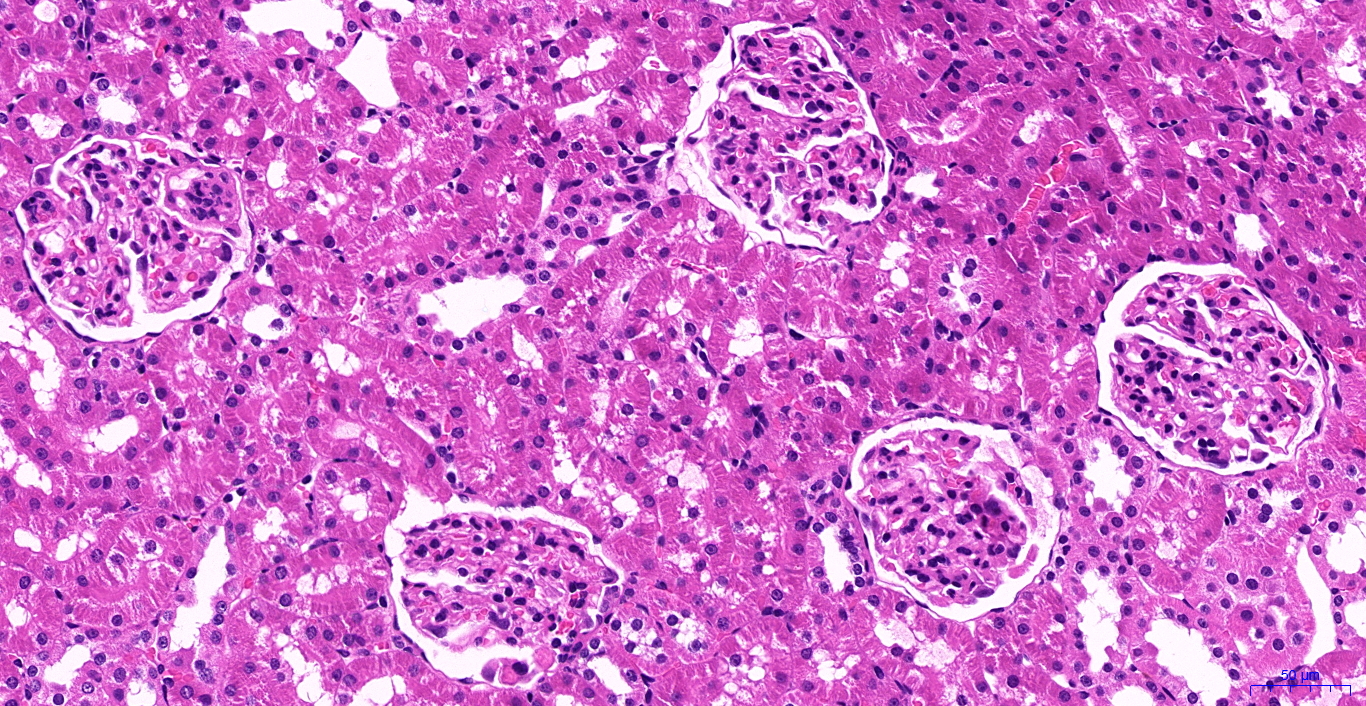

Supplement: Supplementary file 5 [file DataSheet2.zip › Supplementary Figure 1 A/SHAM HE_20.0x.jpg]

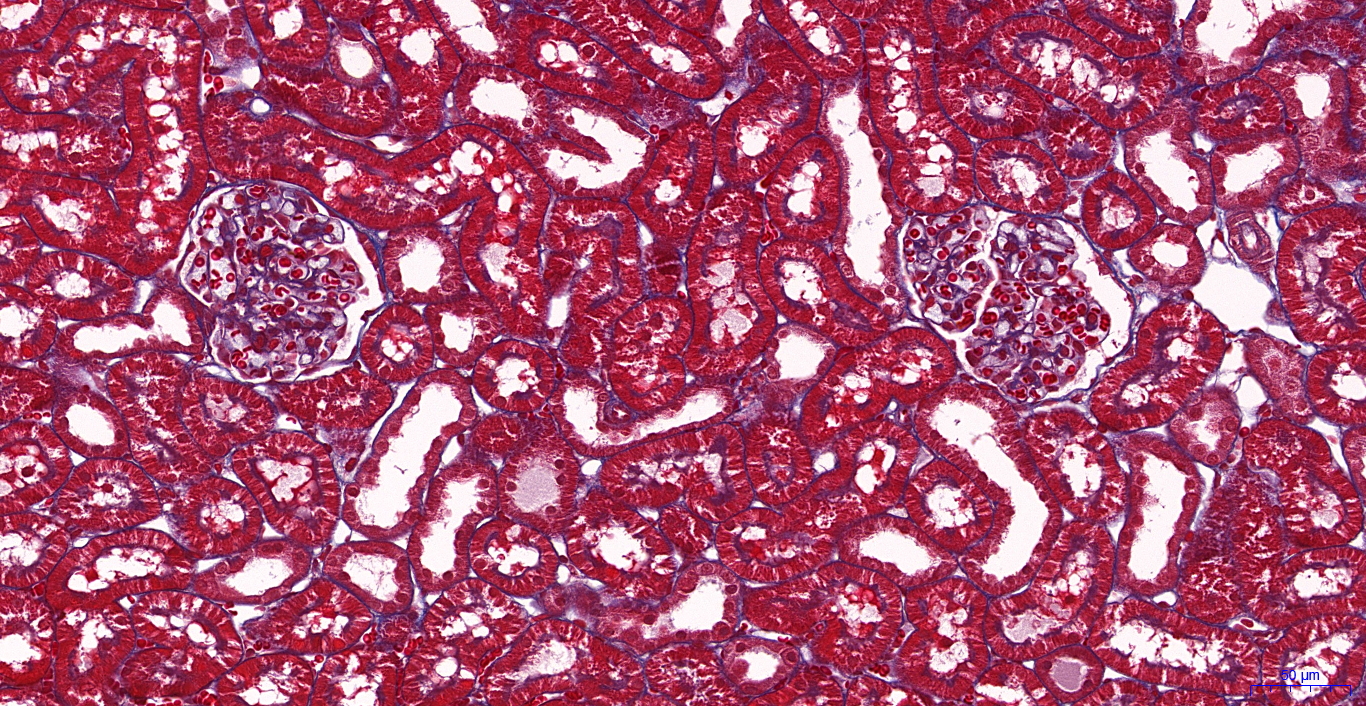

Supplement: Supplementary file 5 [file DataSheet2.zip › Supplementary Figure 1 A/SHAM MASSON_20.0x.jpg]

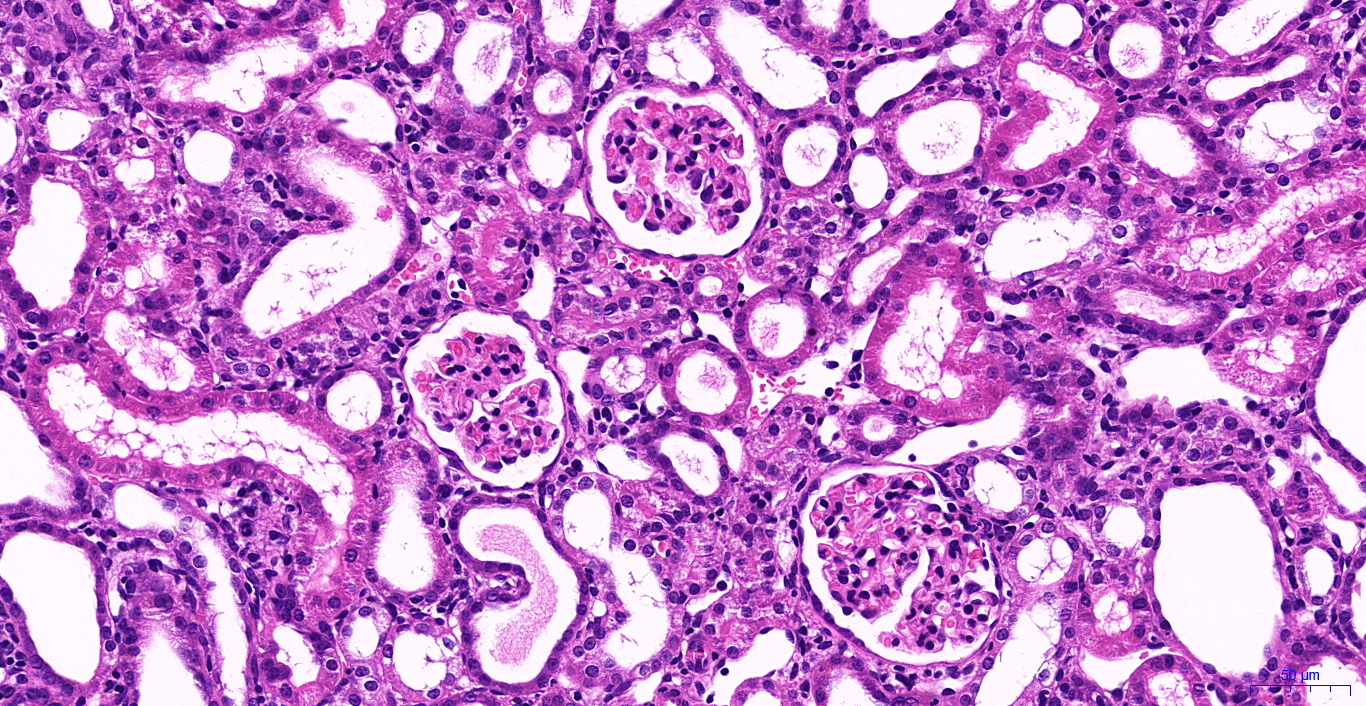

Supplement: Supplementary file 5 [file DataSheet2.zip › Supplementary Figure 1 A/UUO HE_20.0x.jpg]

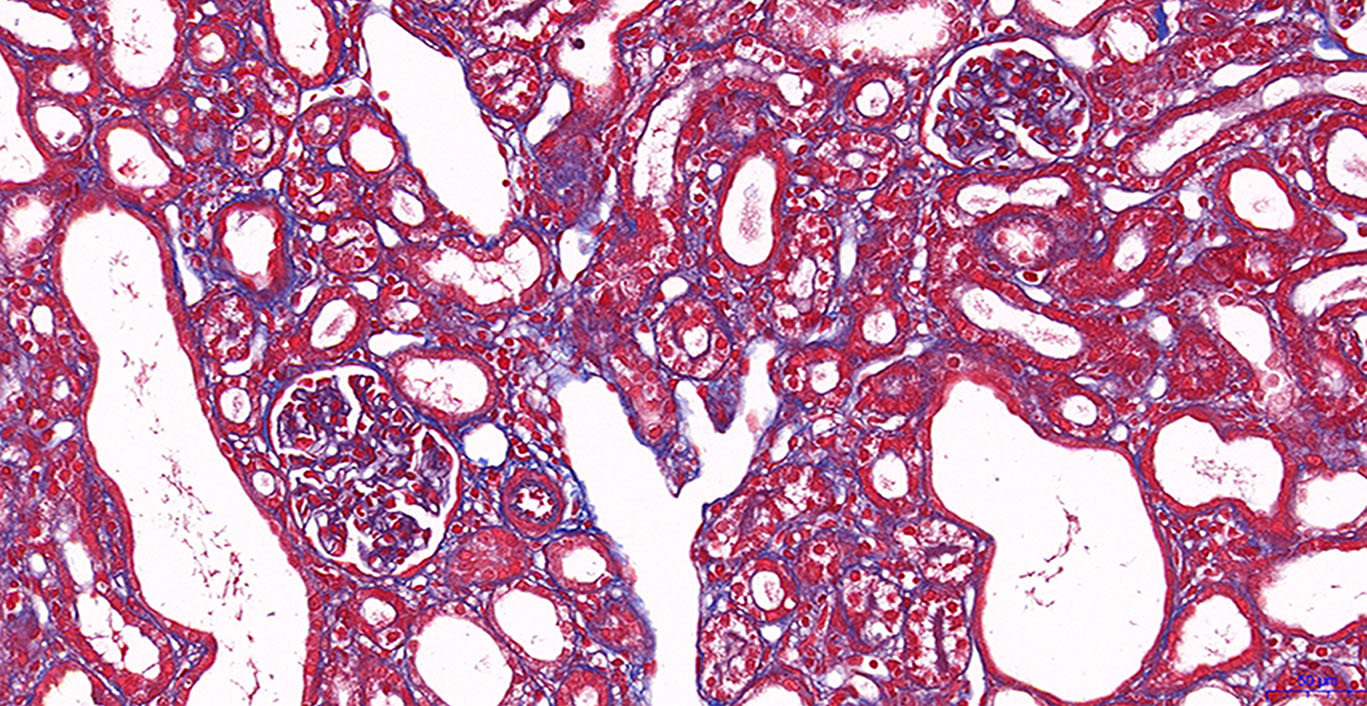

Supplement: Supplementary file 5 [file DataSheet2.zip › Supplementary Figure 1 A/UUO MASSON_20.0x.jpg]

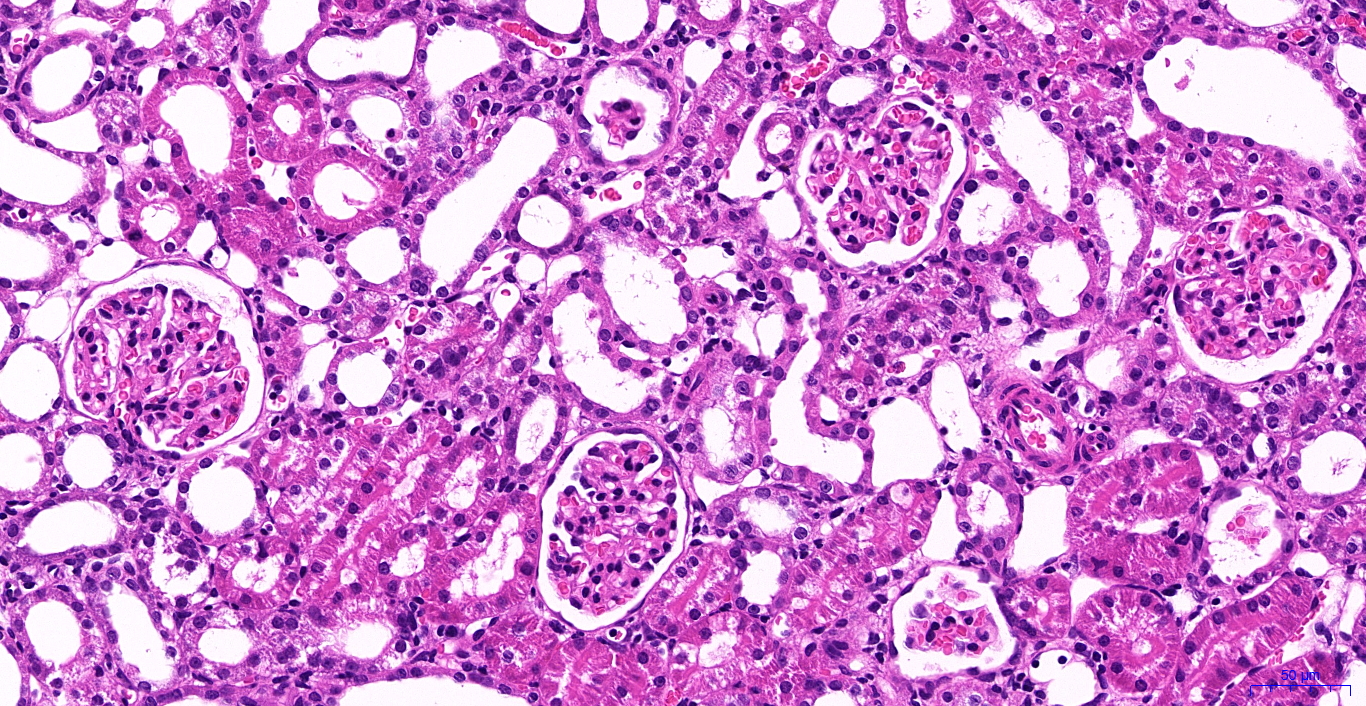

Supplement: Supplementary file 5 [file DataSheet2.zip › Supplementary Figure 1 A/UUO+EPO HE_20.0x.jpg]

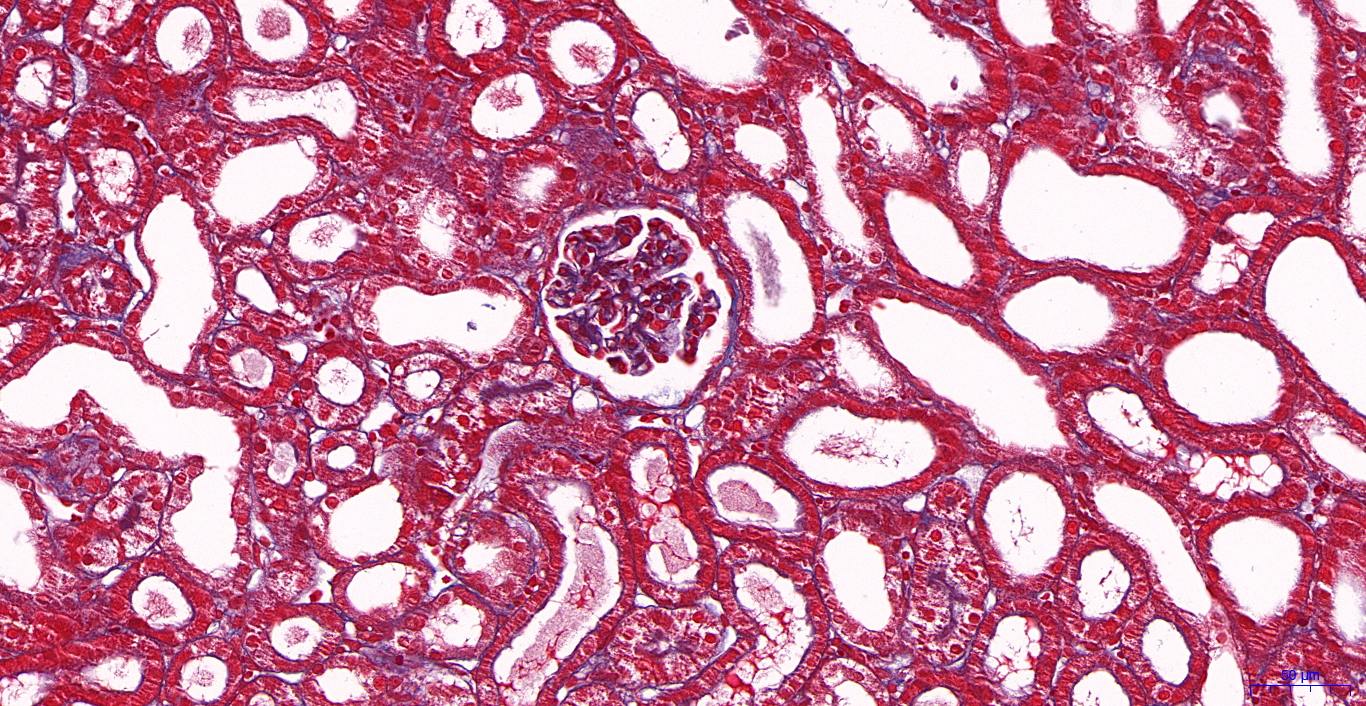

Supplement: Supplementary file 5 [file DataSheet2.zip › Supplementary Figure 1 A/UUO+EPO MASSON_20.0x.jpg]

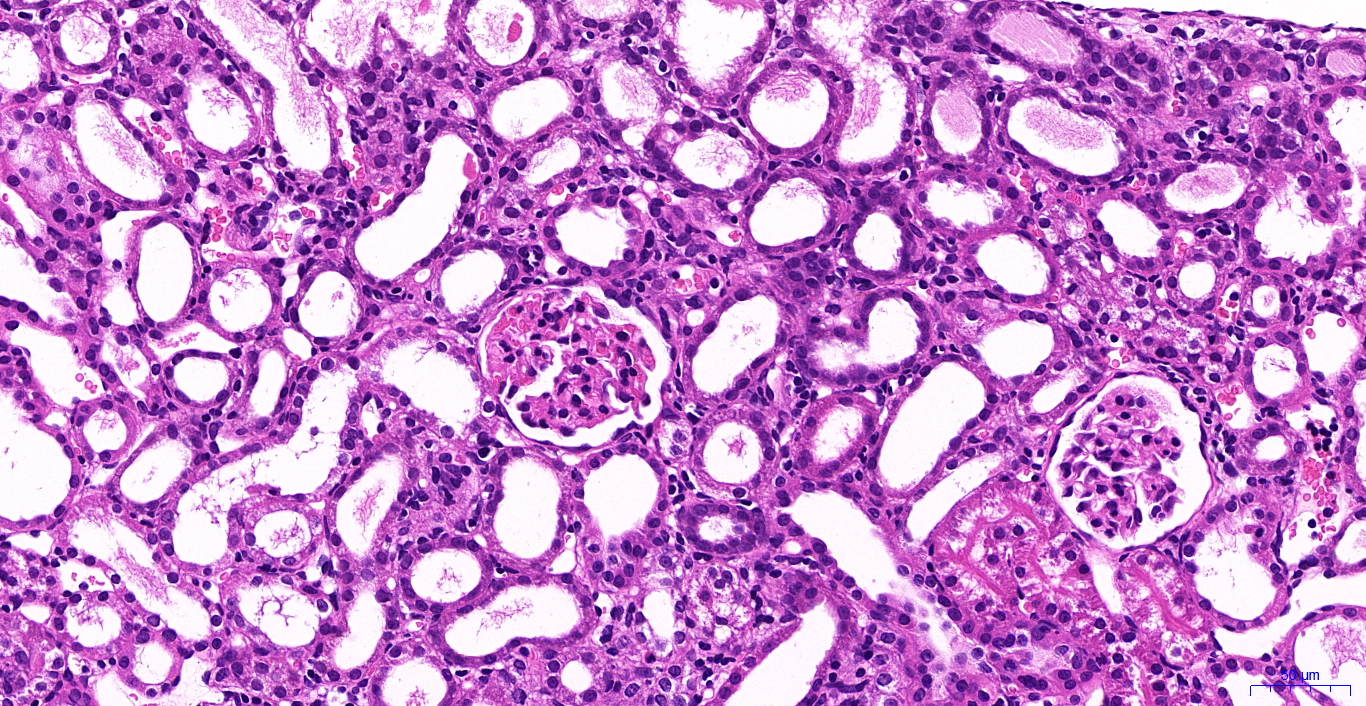

Supplement: Supplementary file 5 [file DataSheet2.zip › Supplementary Figure 1 A/UUO+saline HE_20.0x.jpg]

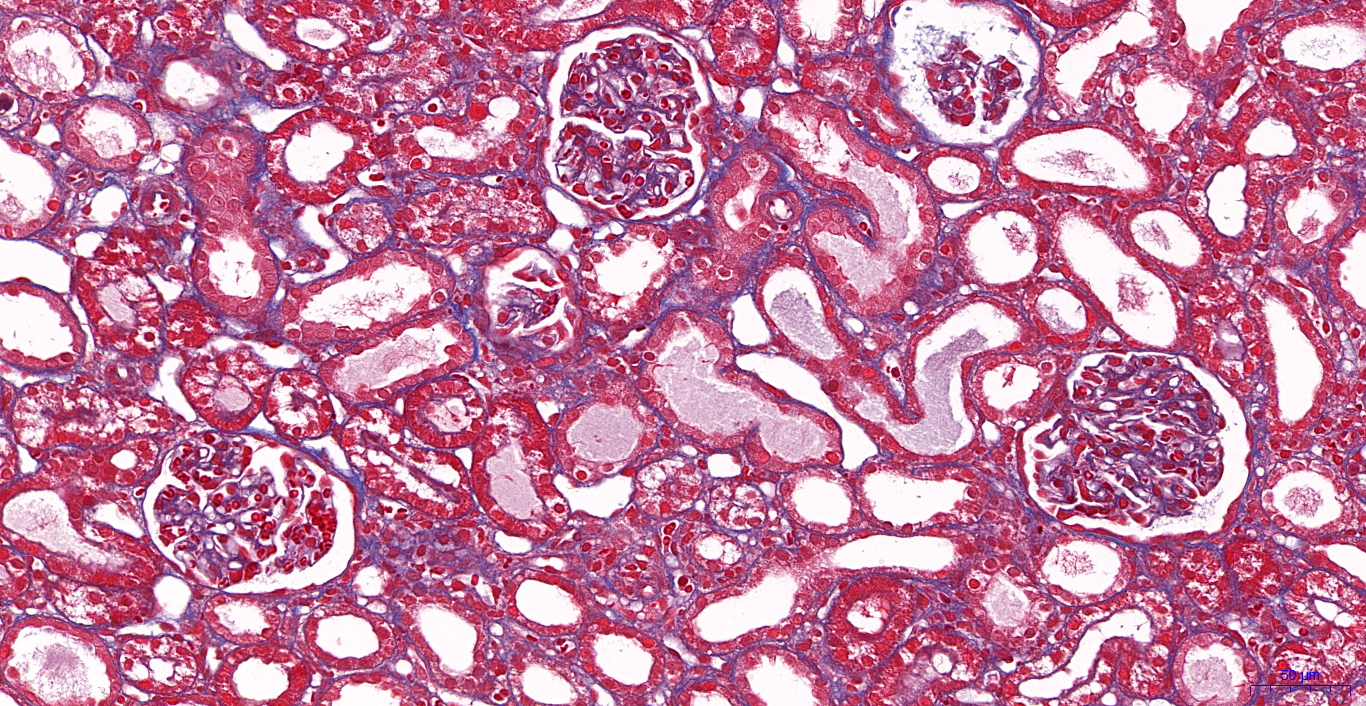

Supplement: Supplementary file 5 [file DataSheet2.zip › Supplementary Figure 1 A/UUO+saline MASSON_20.0x.jpg]

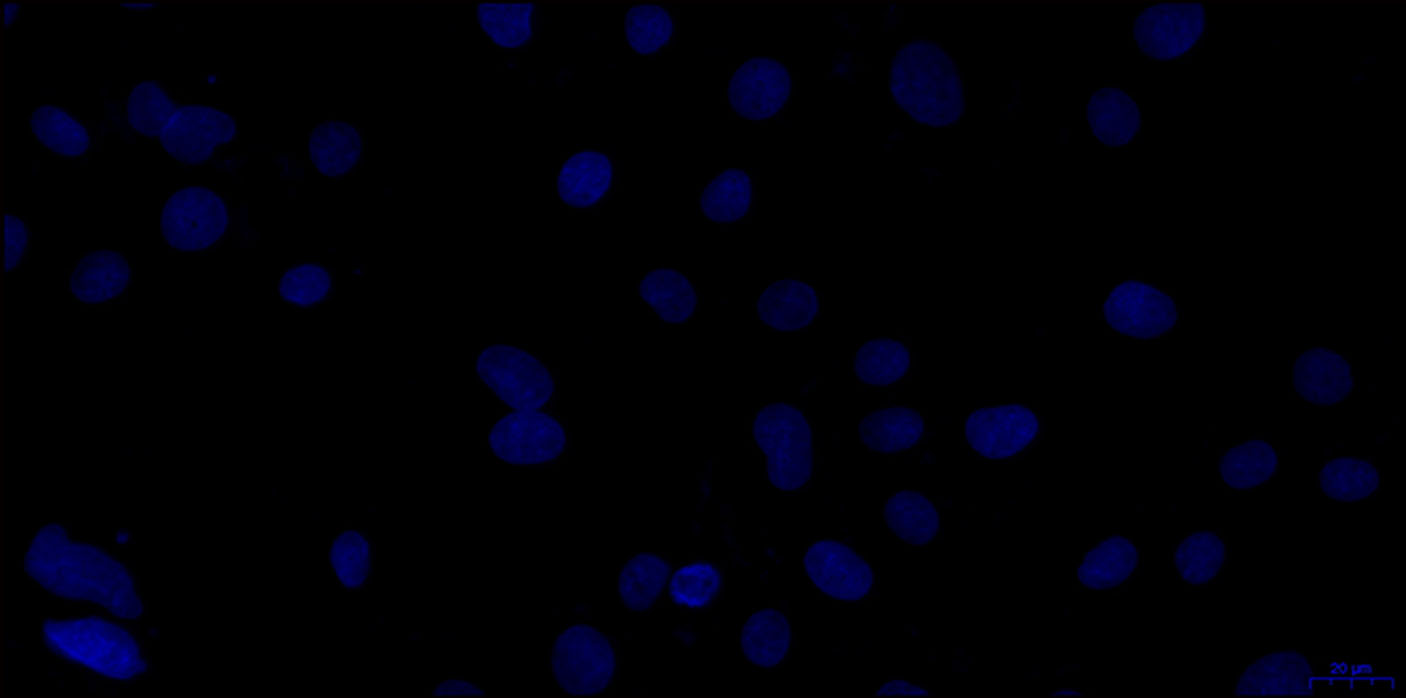

Supplement: Supplementary file 6 [file DataSheet5.zip › Supplementary Figure 5 H/Control DIAP.jpg]

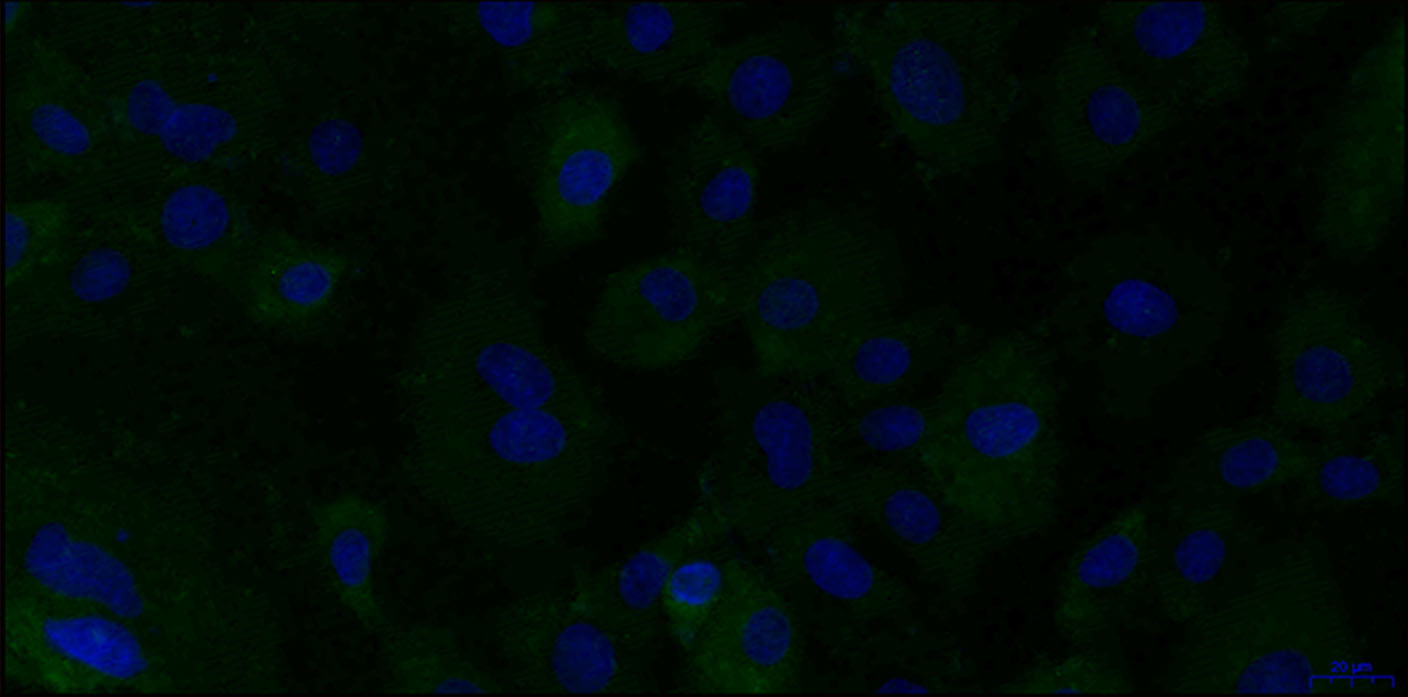

Supplement: Supplementary file 6 [file DataSheet5.zip › Supplementary Figure 5 H/Control Merge.jpg]

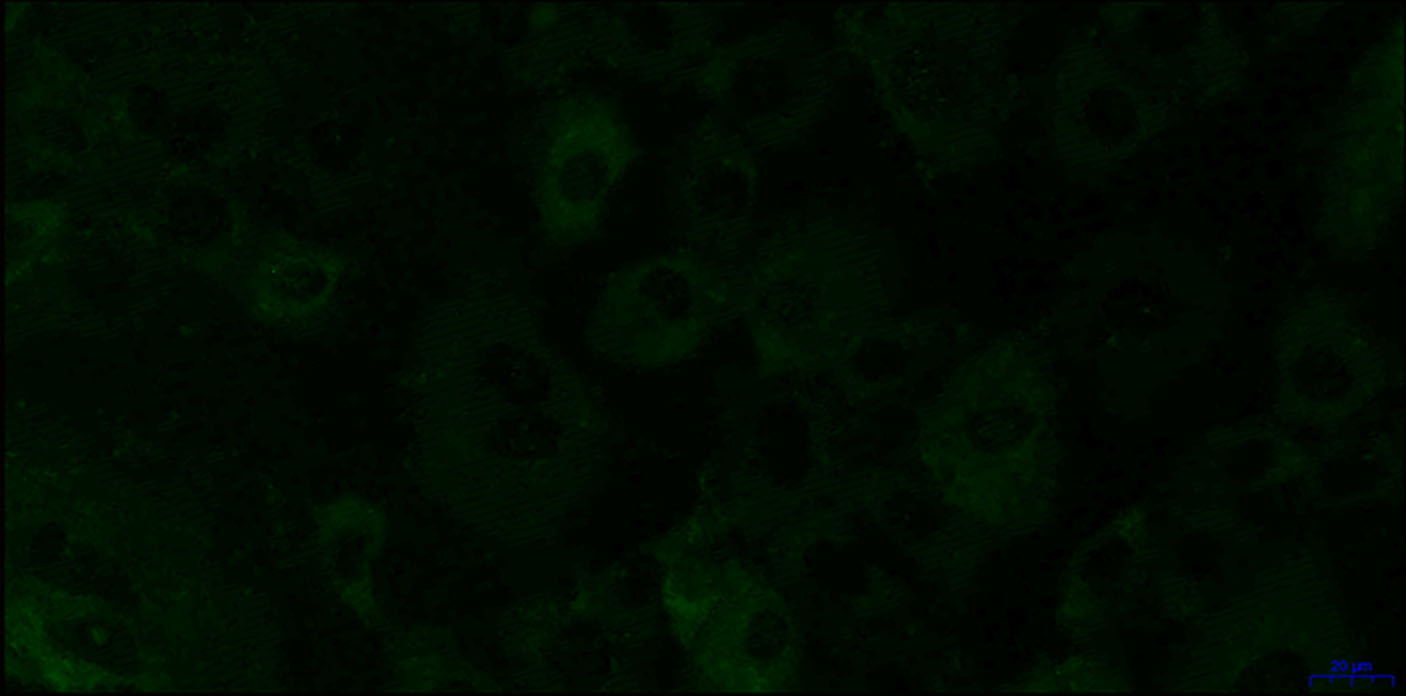

Supplement: Supplementary file 6 [file DataSheet5.zip › Supplementary Figure 5 H/Control α-SMA.jpg]

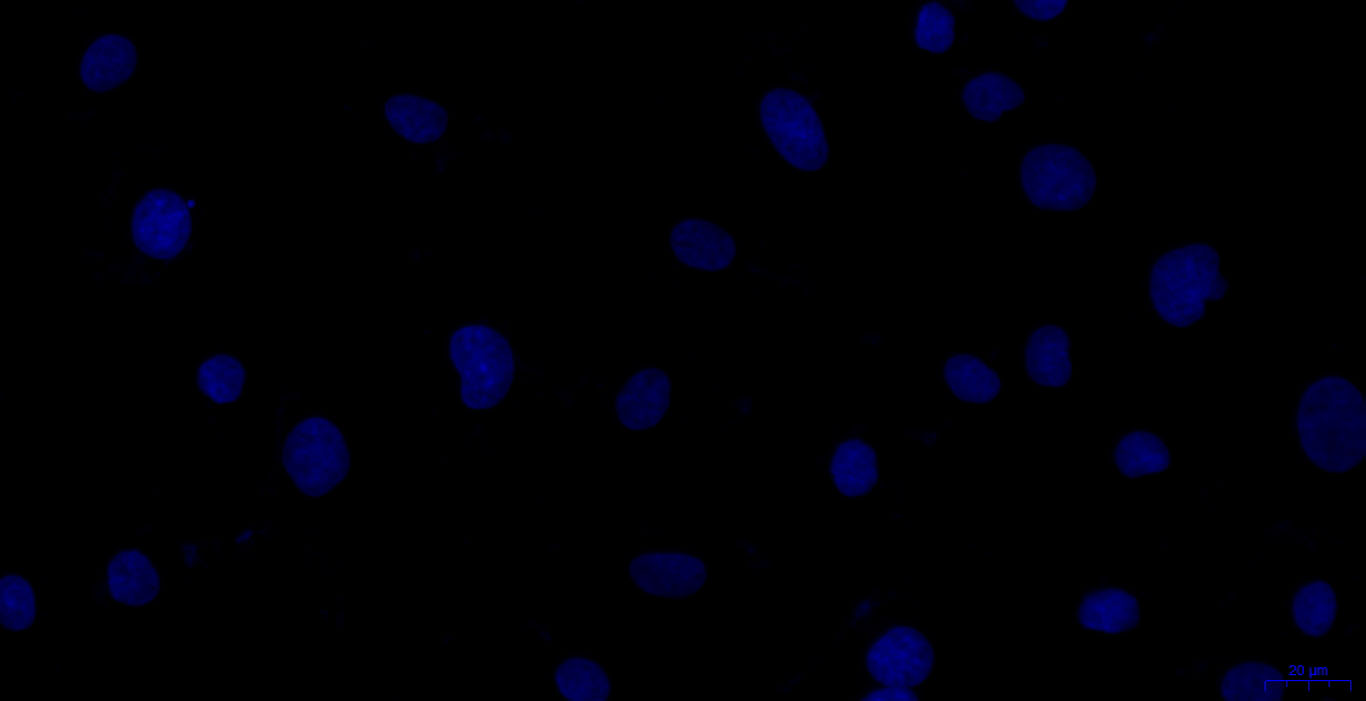

Supplement: Supplementary file 6 [file DataSheet5.zip › Supplementary Figure 5 H/TGFβ1 DIAP.jpg]

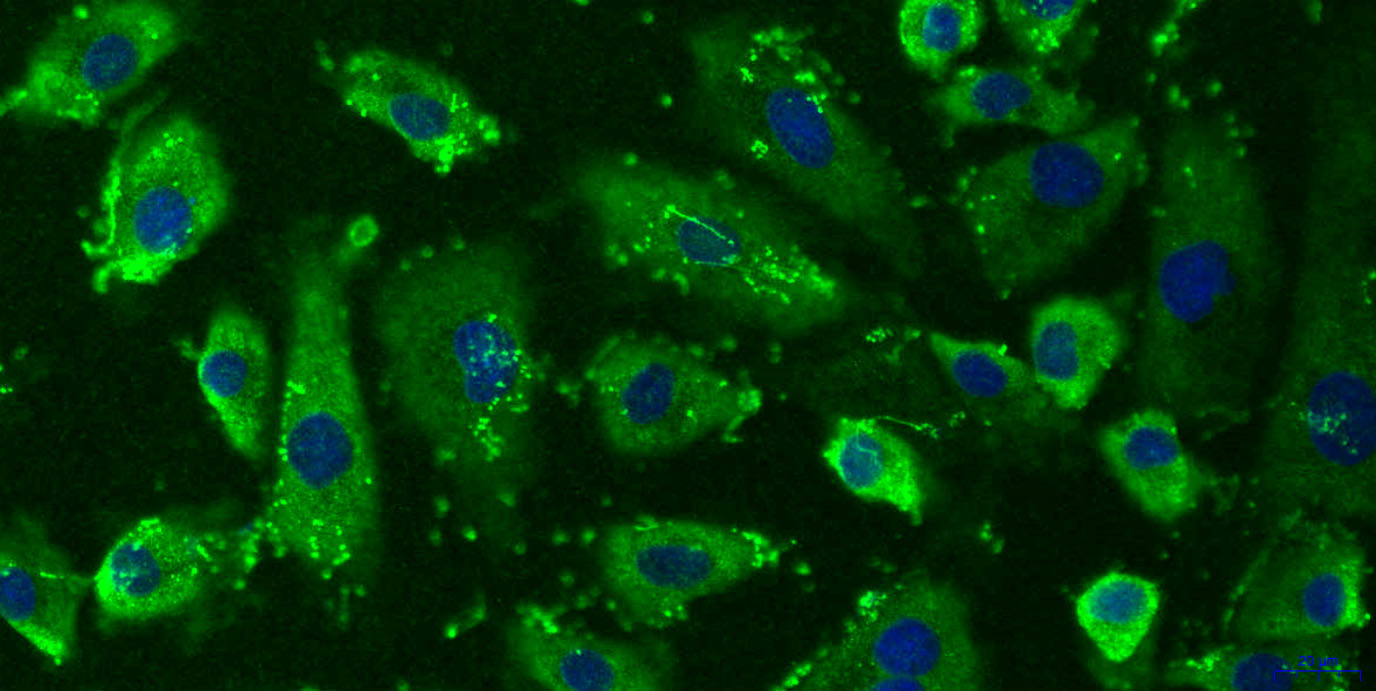

Supplement: Supplementary file 6 [file DataSheet5.zip › Supplementary Figure 5 H/TGFβ1 Merge.jpg]

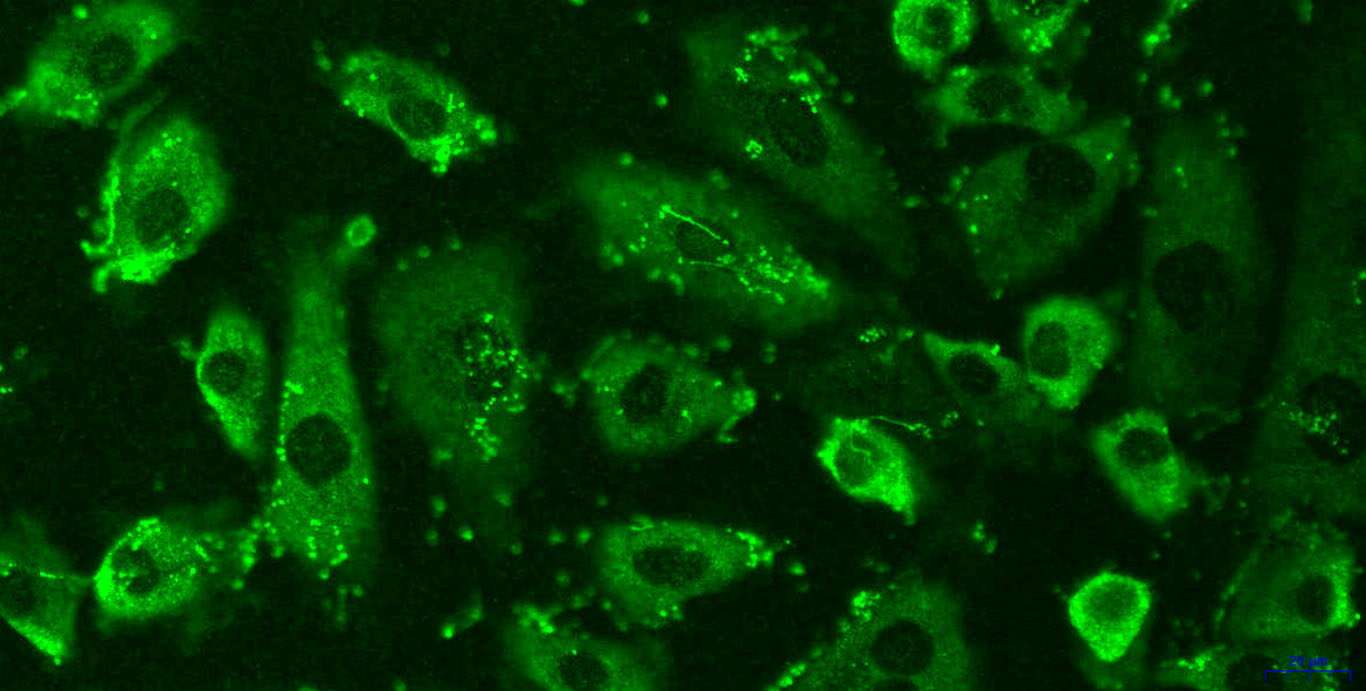

Supplement: Supplementary file 6 [file DataSheet5.zip › Supplementary Figure 5 H/TGFβ1 α-SMA.jpg]

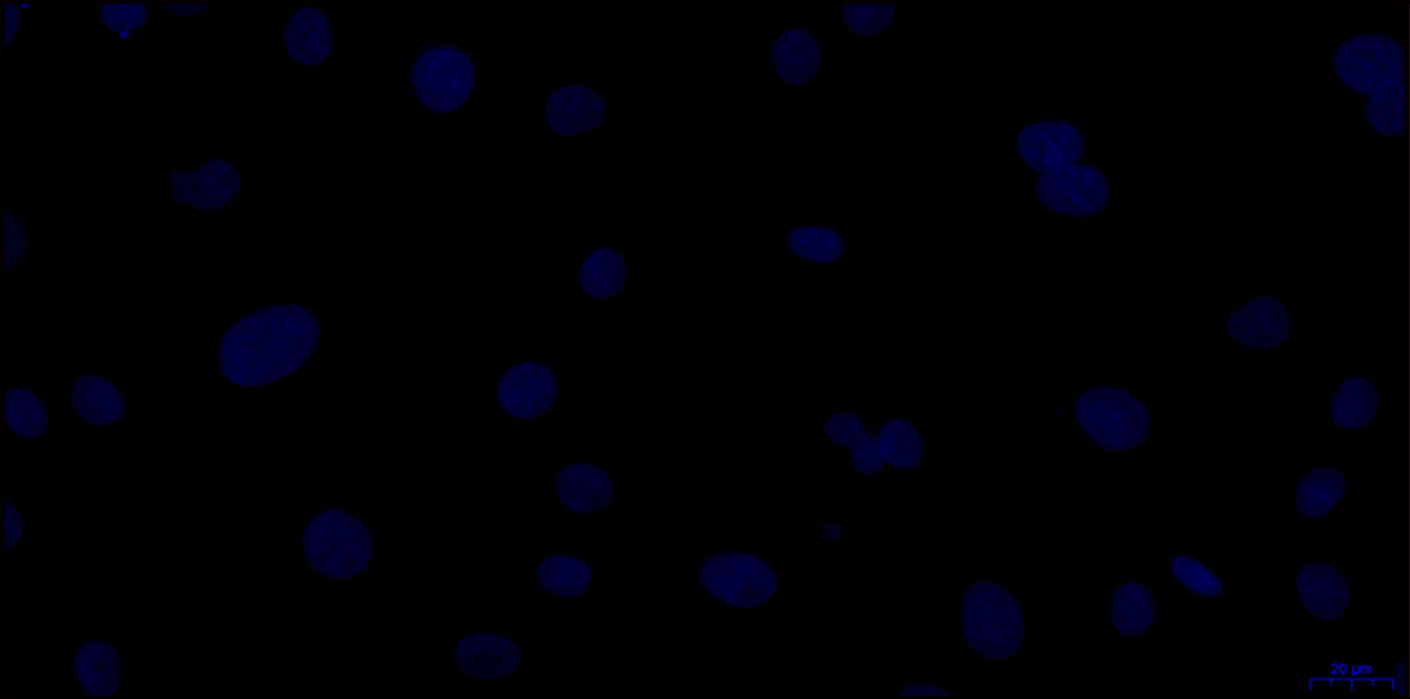

Supplement: Supplementary file 6 [file DataSheet5.zip › Supplementary Figure 5 H/TGFβ1+EPO DIAP.jpg]

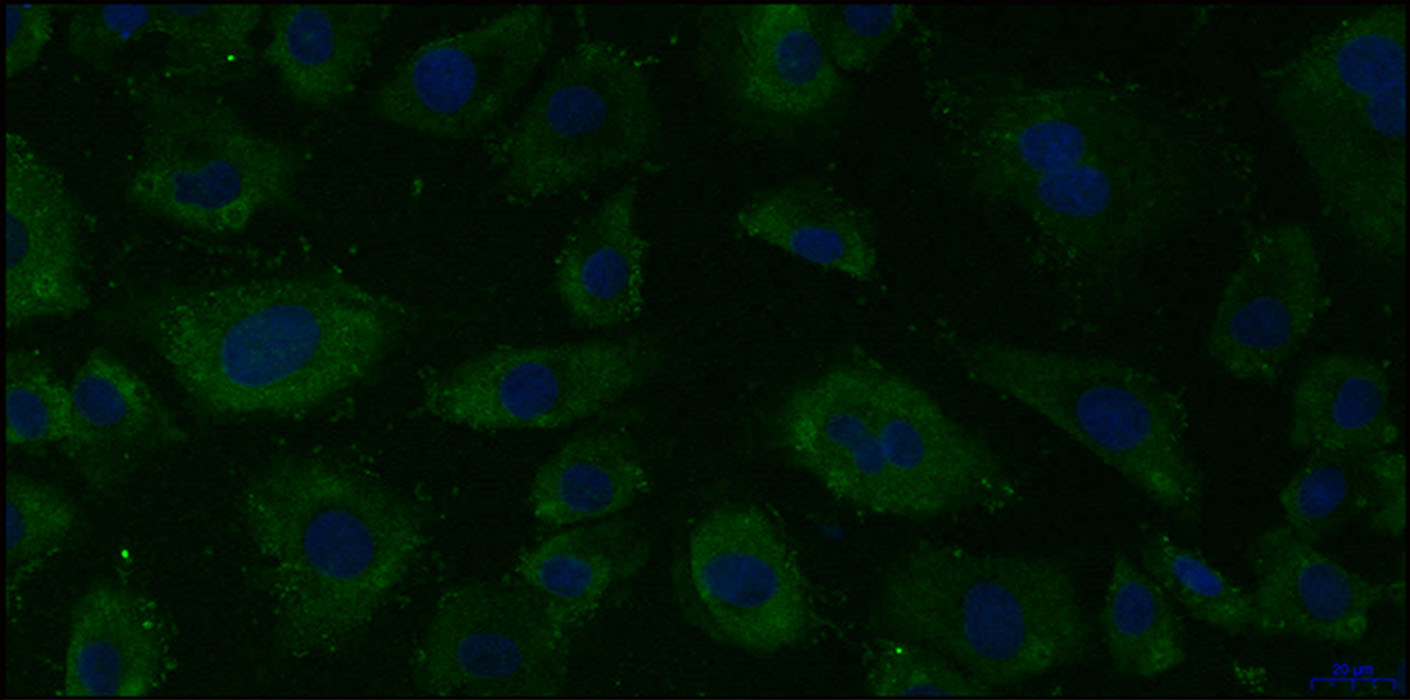

Supplement: Supplementary file 6 [file DataSheet5.zip › Supplementary Figure 5 H/TGFβ1+EPO Merge.jpg]

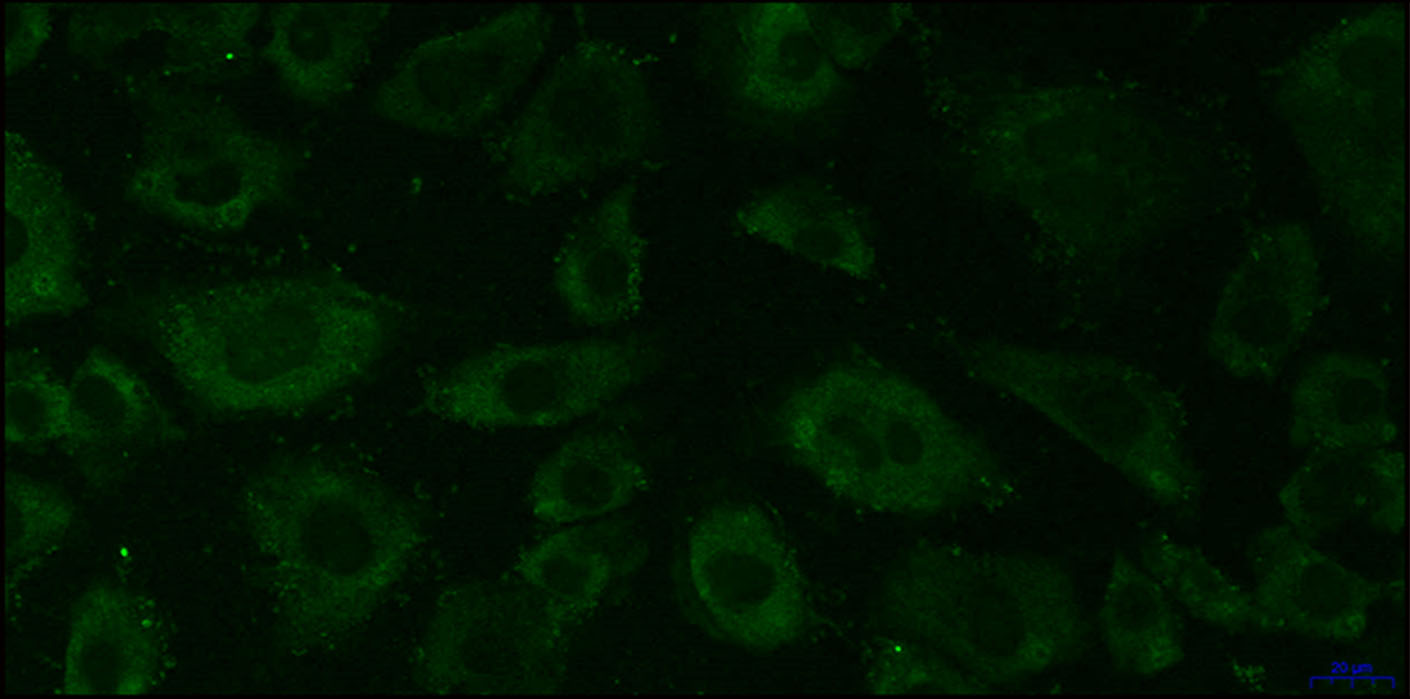

Supplement: Supplementary file 6 [file DataSheet5.zip › Supplementary Figure 5 H/TGFβ1+EPO α-SMA.jpg]
